# Spontaneous coronary artery dissection in regions of non-Western populations: a systematic literature search and scoping review

**DOI:** 10.1093/ehjopen/oeaf022

**Published:** 2025-03-19

**Authors:** Rasha Kaddoura, Ashraf Ahmed, Mohammed Al-Hijji, Fathima Aaysha Cader, Mirvat Alasnag, David Adlam

**Affiliations:** Pharmacy Department, Hamad Medical Corporation, Heart Hospital, P.O. Box 3050, Doha, Qatar; Department of Internal Medicine, Yale New Haven Health, Bridgeport Hospital, 267 Grant Street, Bridgeport, CT 06610, USA; Cardiology Department, Heart Hospital, Hamad Medical Corporation, P.O. Box 3050, Doha, Qatar; Department of Cardiology, Kettering General Hospital, Kettering, Northants NN16 8UZ, UK; Ibrahim Cardiac Hospital & Research Institute, 122 Kazi Nazrul Islam Ave, Dhaka 1000, Bangladesh; Cardiac Center, King Fahd Armed Forces Hospital, P.O. Box 126418, Jeddah 21372, Saudi Arabia; Department of Cardiovascular Sciences, NIHR Leicester Biomedical Research Centre, University of Leicester, Leicester, UK

**Keywords:** False lumen, Intima, MENA, Scaffold, South Asia

## Abstract

Current data on spontaneous coronary artery dissection (SCAD) predominantly originate from Europe or Caucasians with limited information about other ethnicities. This scoping review presents the evidence on SCAD in non-Western countries. The prevalence of SCAD diagnosis was 1.0% [95% confidence interval (95% CI): 0.0–3.0, *I*^2^ = 99%] among patients presenting with myocardial infarction, increasing to 5.0% (95% CI: 1.0–16.0, *I*^2^ = 99%) among females. The mean age was 51.3 years with females accounting for 54.0% of patients. A conservative revascularization management was reported in 48.0% (95% CI: 32.0–65.0, *I*^2^ = 85%) of patients. Antiplatelet therapy was reported as dual (68.0%, 95% CI: 37.0–89.0, *I*^2^ = 90%) in only three studies. A death rate (3.0%) was similar during hospitalization and at mean follow-up of 23.42 months. In conclusion, there is a marked variability in the clinical course of patients with SCAD as well heterogeneity among the included studies. This review identified knowledge gaps in our understanding of SCAD in non-Western countries that should be addressed in future prospective studies.

## Graphical Abstract

**Graphical Abstract:**
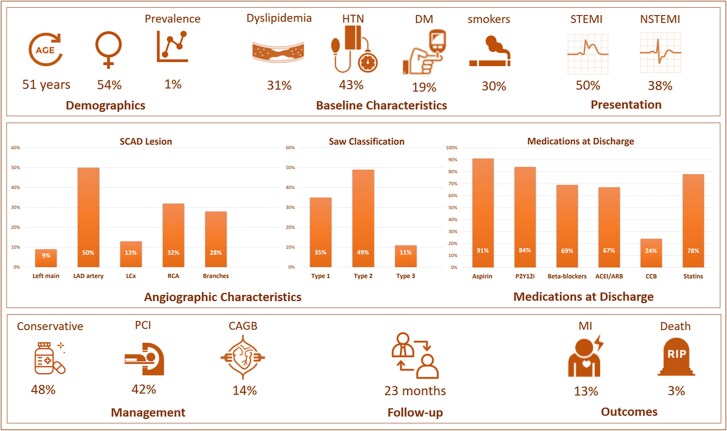

**Learning points**

1. Evidence on spontaneous coronary artery dissection (SCAD) generally originates from the developed continents wherein most patients are Caucasian with a knowledge gap about SCAD in other ethnicities.
2. Available evidence on SCAD in the non-Western population is limited to retrospective observational studies with a noticeable variability in geographical regions, genetic predisposition, cultural and socioeconomic considerations, ethnic diversity, medical services, and clinical practice.
3. Characteristics and clinical outcomes of patients with SCAD in the non-Western countries may differ from their European counterparts.

## Introduction

Spontaneous coronary artery dissection (SCAD) is an important cause of acute myocardial infarction (AMI). It has been an evolving entity since its first description in 1931.^1^ Spontaneous coronary artery dissection is caused by an intramural haematoma, leading to the creation of a false lumen in tunica media. Pathophysiological mechanisms include endothelial dissection that creates a false lumen or intramural haematoma that obstructs the coronary artery lumen.^2^ Spontaneous coronary artery dissection is not iatrogenic or related to trauma or atherosclerosis.^3^ The clinical presentation of SCAD varies from chest pain to sudden cardiac death. Patients may have ST-segment elevated myocardial infarction (STEMI) or non-STEMI (NSTEMI) based on the degree of coronary outflow obstruction caused by the false lumen and haematoma.^4^ Angiographically, diagnosis of SCAD can be challenging and is aided by intracoronary imaging which allows visualization of the false lumen.^5^ The true prevalence of SCAD is not certain as it may be underdiagnosed.^6^ Its incidence is highest in young to middle-aged females.^3^ Classical atherosclerotic cardiovascular risk factors are lower than those of atherosclerotic patients with AMI.^3^

Although our understanding of SCAD has substantially improved, it is still limited with regard to ethnic disparities.^7^ The available leading studies originate from the developed continents,^8^ particularly Europe and North America, wherein the majority of enrolled patients were Caucasian, with underrepresentation of other ethnicities.^3,7^ Ethnic differences in cardiovascular risk factors have been identified across a broad range of clinical, environmental, lifestyle, and sociodemographic risk factors.^9,10^ It is also known that there is an underlying genetic susceptibility to SCAD, but it is unclear whether certain ethnicities have specific genetic predisposition. This scoping review primarily aimed to identify the current literature on SCAD in non-Western countries with an overarching objective to highlight gaps in research knowledge needing further investigation.

## Methods

A scoping review was conducted to address the objective. A scoping review is a systematic approach to knowledge synthesis that maps evidence on a specific topic to recognize essential concepts, theories, evidence sources, and knowledge gaps for research.^11^ It allows a preliminary assessment of the extent of available literature and characterizes the quantity and quality of included studies, in a narrative manner, by their key features without a formal quality assessment.^12^ This scoping review was conducted according to the Preferred Reporting Items for Systematic reviews and Meta-Analyses extension for Scoping Reviews (PRISMA-ScR) statement and checklist,^11^ as well as the steps proposed by Arskey and O’Malley.^13^

### Eligibility criteria

The inclusion criteria allowed any study design, including case series of more than four cases,^14^ that reported SCAD’s incidence or prevalence, epidemiology, comorbidities, risk, and precipitating factors, genetics, management, and clinical outcomes in adult patients. The studies were required to originate from the countries that belong to the pre-specified regions which include the World Health Organization (WHO)-defined Eastern Mediterranean region, South-East Asia region, and Western Pacific Region,^15^ plus Turkey. Abstracts, conference proceedings, study protocols, unpublished studies, reviews of any type, animal models, case reports, or case series of less than five cases were excluded.

### Search strategy

A comprehensive literature search using MEDLINE and EMBASE (OVID® interface) was conducted on 28 December 2023 and updated on 24 April 2024. Broad keywords, Medical Subject Headings, and Emtree terms were combined with Boolean terms ‘AND’ and ‘OR’. The search terms and their combination included: ‘SCAD’, ‘spontaneous coronary artery dissection’, ‘Asia’, ‘Mediterranean’, ‘Pacific’, and the individual country of each region (see Supplementary material online, *Tables S1* and *S2*). The references’ lists of the retrieved studies, reviews, meta-analyses, and international registries were manually screened to identify additional studies.

### Study selection and data extraction

The search records were examined at title and abstract levels. Potentially eligible abstracts were retrieved in full text and reviewed in duplicate to ascertain eligibility. The data were extracted and compiled in supplementary tables. The data stems included study objective(s), design, recruitment period, conclusion, study characteristics, eligibility criteria, diagnosis, patient characteristics, interventions, and clinical outcomes.

### Data synthesis and analysis

Due to the heterogeneity between the studies and the wide range of the reported variables, we opted to pool the proportions and means of categorical and continuous variables, respectively, with 95% confidence interval (95% CI). A continuous variable reported as median (interquartile range) was converted into a mean (standard deviation). Sensitivity analyses were performed according to study size by removing the studies of sample size of less than 50 patients and according to confirmed SCAD diagnosis by removing the studies that did not clearly include coronary angiographic assessment. Statistical heterogeneity was investigated by the inconsistency factor (*I*^2^). The *I*² values >50% represent high heterogeneity.^16,17^ The analysis was performed by R software (RStudio 2023.06.0 + 421).

## Results

### Search results and study characteristics

**Figure 1:**
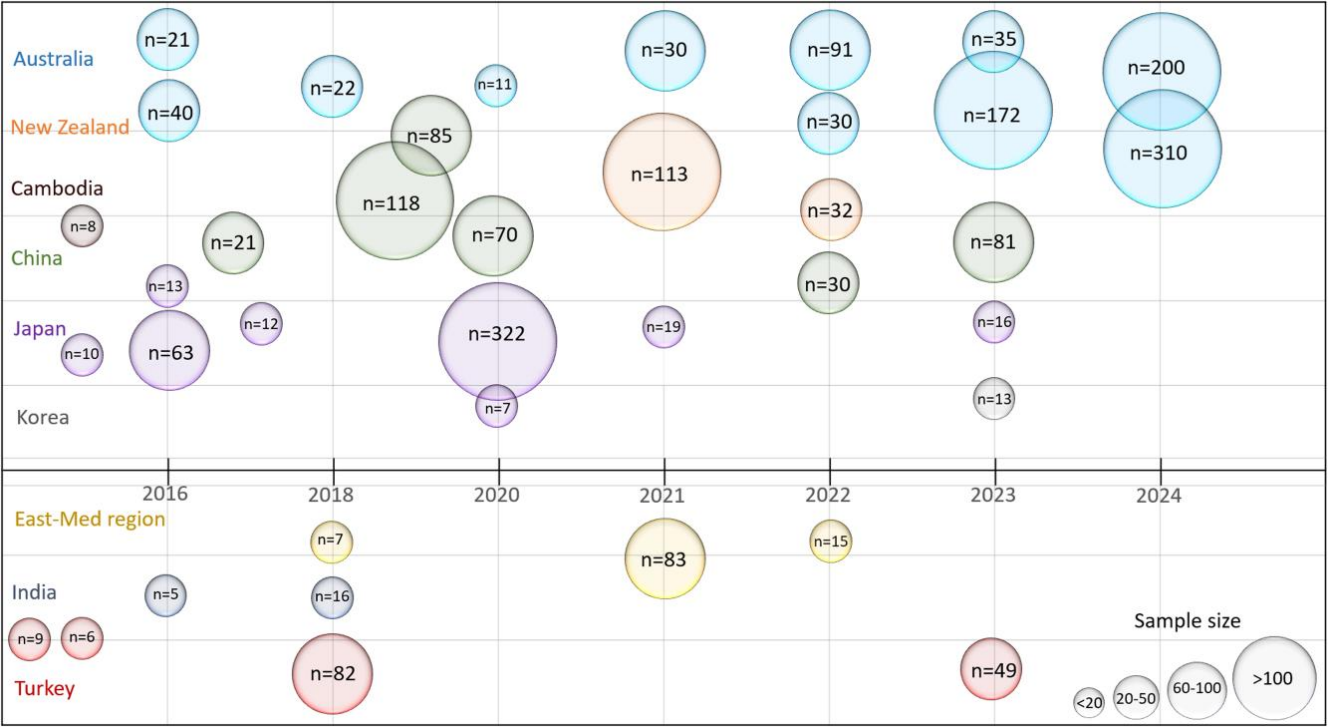
Timeline of the included studies.

After eliminating duplicates, including 11 articles that were identified from manually searching the reference lists of the excluded review articles, 881 records were screened at title and abstract levels. After excluding ineligible records (i.e. reviews, conference proceedings, case reports, studies from other regions, animal models, and non-SCAD topics), 65 articles were retrieved in full text. Of these, 27 articles were excluded (see Supplementary material online, *Table S3*) and 38 studies met the inclusion criteria (see Supplementary material online, *Figure S1*). The largest proportion of the 38 studies were conducted in Australia and New Zealand (13 studies), China (6 studies), and Japan (8 studies) (*Figure 1*). Although Australia and New Zealand are within the geopolitical location of the specified WHO regions, the main ethnic group in these countries is the Europeans (> 70.0%). Caucasian ethnicity reported in these two countries ranged from 81.0 to 92.0% of patients in three of the retrieved studies that reported ethnicity information (see Supplementary material online, *Table S4*). Thus, the data of the 13 studies from these two countries are only presented in the supplementary tables for completeness and were not discussed or pooled with other studies. Similarly, a case series of eight Cambodian patients was not discussed as six patients (75.0%) were of Caucasian ethnicity (see Supplementary material online, *Table S4*). The remaining 24 studies recruited 1092 patients with SCAD from at least 73 centres in 12 countries between 1994 and 2022 with an individual sample size ranging from 5 to 322 patients.^18–41^ Only two studies included more than 100 patients.^25,34^ The included studies were of retrospective cross sectional or descriptive, cohort, case–control, or case series (>four cases) design. The general aspects of the studies are detailed in *Table 1* and Supplementary material online, *Tables S5–S7*.

**Table 1:** General study characteristics

Variable	Eastern Mediterranean region	South-East Asia region	Western Pacific Region		Turkey
	Gulf, Iran, and Morocco	India	China, Japan, and South Korea	Australia, New Zealand, and Cambodia	
Number of studies	3	2	15	14	4
Publication date					
Before 2014	0	0	0	0	2
2014 onwards	3	2	15	14	2
Study design					
Case–control	0	0	2	1	0
Case series (>four cases)	1	1	3	1	2
Cohort	1	0	1	1	1
Descriptive	1	1	9	5	1
Survey	0	0	0	3	0
Whole-genome sequencing	0	0	0	2	0
Qualitative	0	0	0	1	0
Recruitment					
Period	2011–2020	1994–2017	2000–2020	2000–2023	2001–2022
Duration (year)	3–7	4–11.5	4–14	0.5–16	2–6
Sample size (SCAD)	7–83	5–16	7–322	8–310	6–82
Total patients with SCAD	105	21	880	1170	86

SCAD, spontaneous coronary artery dissection.

### Prevalence of SCAD

The pooled prevalence of SCAD from all studies was 1.0% (95% CI: 0.0; 3.0, *I*^2^ = 99%) of patients with AMI or those who underwent coronary angiography (see Supplementary material online, *Table S7*). Prevalence did not change when removing the studies that enrolled only females (1.0%, 95% CI: 0.0; 2.0, *I*^2^ = 99%), neither did the high heterogeneity.^20,23,34,35,37,41^ However, when the studies that included females only were pooled separately, the prevalence of SCAD was 5.0% (95% CI: 1.0; 16.0, *I*^2^ = 99%) (see Supplementary material online, *Figures S2*–*S4*) with high heterogeneity. One study recruited only patients with spontaneous coronary intramural haematoma with prevalence rate of 0.4% of those presented with acute coronary syndrome.^27^

### Patient characteristics

The pooled mean age was 51.3 years (95% CI: 48.79; 53.82, *I*^2^ = 94%). Females accounted for 54.0% (95% CI: 38.0; 70.0, *I*^2^ = 89%) of patients, with six studies recruiting only females (100%) with an age ranged between 44.0 and 55.0 years.^20,23,34,35,37,41^ Traditional cardiovascular risk factors and comorbidities included dyslipidaemia (31.0%, 95% CI: 26.0; 37.0, *I*^2^ = 58%), hypertension (43.0%, 95% CI: 36.0; 51.0, *I*^2^ = 74%), diabetes (19.0%, 95% CI: 13.0; 27.0, *I*^2^ = 80%), smoking status (30.0%, 95% CI: 23.0; 39.0, *I*^2^ = 79%), and prior myocardial infarction (11.0%, 95% CI: 5.0; 24.0, *I*^2^ = 90%). History of fibromuscular dysplasia (FMD) was reported in 22.0% (95% CI: 7.0; 50.0, *I*^2^ = 88%) of patients (see Supplementary material online, *Figures S5*–*S12*). Stress as a precipitating factor was reported in 17.0% (95% CI: 7.0; 34.0, *I*^2^ = 66%) and 29.0% (95% CI: 16.0; 46.0, *I*^2^ = 66%) of patients for physical and emotional stress, respectively (see Supplementary material online, *Figures S13* and *S14*). Heterogeneity was high for all aforementioned pooled variables. Among females with SCAD, 25.0% (95% CI: 12.0; 44.0, *I*^2^ = 79%) were in menopause,^19,22,23,26,30,37^ with one study reporting a lower proportion of females in early menopause (5.0%) due to the inclusion of only young females (≤50 years) with AMI.^30^ Sensitivity analysis by removing the latter study yielded a rate of 34.0% (95% CI: 23.0; 47.0, *I*^2^ = 53%) with a relatively improved heterogeneity (see Supplementary material online, *Figures S15* and *S16*). Detailed demographics, baseline characteristics, and comorbidities are outlined in Supplementary material online, *Tables S8*–*S11*.

### Management of SCAD

#### Clinical presentation

At presentation, symptom frequency was reported in only two studies [chest pain (92.0–93.0%)^26,37^ and dyspnoea (8.0%)^37^]. ST-segment elevated myocardial infarction was diagnosed in 50.0% (95% CI: 38.0; 62.0, *I*^2^ = 79%) of patients. Non-STEMI and unstable angina were diagnosed in 38.0% (95% CI: 25.0; 52.0, *I*^2^ = 86%) and 18.0% (95% CI: 8.0; 33.0, *I*^2^ = 87%), respectively (see Supplementary material online, *Figures S17*–*S19*). Two studies recruited only patients with STEMI (100%).^20,21^ Three studies reported cardiac arrest/ventricular arrhythmia (4.0–16.0%)^19,24,30^ and another two reported heart failure (8.5–11.0%)^25,38^ upon hospital presentation. Only four studies reported serum cardiac troponin values^19,26,28,37^ (see Supplementary material online, *Tables S12* and *S13*). The pooled mean of left ventricular ejection fraction was 51.4% (95% CI: 47.44; 55.36, *I*^2^ = 97%) (see Supplementary material online, *Table S14* and *Figure S20*). High heterogeneity dominates all pooled variables.

#### Management strategy

A conservative revascularization management strategy was reported in almost half of the patients (48.0%, 95% CI: 32.0; 65.0, *I*^2^ = 85%) (see Supplementary material online, *Figure S21*) with high heterogeneity among studies. The pooled proportion for revascularization by percutaneous coronary intervention (PCI) was 42.0% (95% CI: 31.0; 53.0, *I*^2^ = 83%). Among patients who underwent coronary angiography, balloon angioplasty was used in 19.0% (95% CI: 11.0; 33.0, *I*^2^ = 43%) of patients with low heterogeneity, intravascular ultrasound (IVUS) was used in 64.0% (95% CI: 46.0; 79.0, *I*^2^ = 69%), and optical coherence tomography (OCT) was used in 53.0% (95% CI: 4.0; 97.0, *I*^2^ = 88%) of patients with high heterogeneity among studies. Two Japanese studies only recruited patients who underwent OCT-guided PCI (100%) (see Supplementary material online, *Figures S22*–*S25* and *Table S15*).^31,32^ There were insufficient data reported about the percutaneous procedure characteristics (see Supplementary material online, *Table S16*). Pooled proportion for revascularization by coronary artery bypass grafting (CABG) was 14.0% (95% CI: 4.0; 37.0, *I*^2^ = 88%) (see Supplementary material online, *Figure S26*). A higher rate of 78.0% for patients who were revascularized by CABG was reported from a case series of six patients, of whom five patients had lesions in their left main artery.^39^ Fibromuscular dysplasia screening was reported in one study with positive screening results in 20.0% of patients (see Supplementary material online, *Table S14*).^30^ Cardiac rehabilitation and genetic testing were not reported in any study.

#### Coronary angiographic characteristics

Most of SCAD lesions involved a single coronary artery (89.0%, 95% CI: 76.0; 95.0, *I*^2^ = 41%). Two-vessel or multivessel involvement was found in 12.0% (95% CI: 7.0; 20.0, *I*^2^ = 32%) or 10.0% (95% CI: 5.0; 21.0, *I*^2^ = 64%) of patients, respectively. The most frequently affected artery or territory was left anterior descending artery (LAD; 50.0%, 95% CI: 43.0; 57.0, *I*^2^ = 67%), followed by right coronary artery (RCA; 32.0%, 95% CI: 24.0; 42.0, *I*^2^ = 82%), left circumflex artery (LCx; 13.0%, 95% CI: 9.0; 19.0, *I*^2^ = 66%), and left main artery (9.0%, 95% CI: 4.0; 20.0, *I*^2^ = 72%). The latter was observed in five out of six patients (83.3%) in a case series of six patients.^39^ Branch vessels involvement affected 28.0% (95% CI: 19.0; 38.0, *I*^2^ = 59%) of patients. The affected coronary segments were distal in 41.0% (95% CI: 21.0; 65.0, *I*^2^ = 79%), middle in 34.0% (95% CI: 28.0; 40.0, *I*^2^ = 27%), and proximal segment in 31.0% (95% CI: 16.0; 51.0, *I*^2^ = 71%) of patients. With regard to the Saw classification, Type 1 SCAD was present in 35.0% (95% CI: 22.0; 51.0, *I*^2^ = 81%), Type 2 in 49.0% (95% CI: 38.0; 60.0, *I*^2^ = 75%), and Type 3 in 11.0% (95% CI: 6.0; 19.0, *I*^2^ = 70%) of patients. Type 4 was found in three patients (20.0%) in one study.^20^ Details are demonstrated in Supplementary material online, *Table S17* and *Figures S27*–*S44*. High heterogeneity dominates all pooled variables.

#### Medications upon discharge

Aspirin was used in 91.0% (95% CI: 77.0; 97.0, *I*^2^ = 74%) and P_2_Y_12_ inhibitors in 84.0% (95% CI: 74.0; 91.0, *I*^2^ = 80%) of patients. Antiplatelet therapy was reported as single (36.0%, 95% CI: 19.0; 58.0, *I*^2^ = 54%) and dual (68.0%, 95% CI: 37.0; 89.0, *I*^2^ = 90%) therapy in two^27,35^ and three^25,27,35,37^ studies, respectively. The duration of dual therapy was not specified. Beta-blockers were prescribed in 69.0% (95% CI: 55.0; 79.0, *I*^2^ = 75%), angiotensin-converting enzyme inhibitors (ACEI)/angiotensin receptor blockers (ARB) in 67.0% (95% CI: 56.0; 76.0, *I*^2^ = 77%), calcium channel blockers in 24.0% (95% CI: 14.0; 37.0, *I*^2^ = 57%), and statin in 78.0% (95% CI: 64.0; 87.0, *I*^2^ = 91%) of patients (see Supplementary material online, *Table S18* and *Figures S45*–*S52*). High heterogeneity dominates all pooled variables.

### Clinical outcomes

#### In-hospital outcomes

Of the patients who were managed with PCI, 7.0% (95% CI: 2.0; 18.0, *I*^2^ = 9%) experienced PCI-related complications (i.e. stent thrombosis and iatrogenic dissection) reported in three studies.^29,30,32^ In-hospital death occurred in 3.0% (95% CI: 2.0; 5.0, *I*^2^ = 35%) of patients enrolled in four studies.^19,20,25,34^ A case series of six patients reported the death of one of them (17.0%) due to sepsis on Day 30 post-CABG.^39^ The rate of in-hospital any cardiovascular event was 9.0% (95% CI: 5.0; 16.0, *I*^2^ = 0%) reported in two studies.^19,40^ The extension of SCAD lesion occurred in 7.0% (95% CI: 2.0; 23.0, *I*^2^ = 73%) of patients in two studies.^19,30^  Supplementary material online, *Table S19* and *Figures S53*–*S56* detail in-hospital outcomes.

#### Long-term outcomes

The pooled mean of the follow-up period was 23.42 months (95% CI: 15.72; 31.11, *I*^2^ = 100%). All-cause death was pooled from five studies (3.0%, 95% CI: 1.0; 7.0, *I*^2^ = 37%)^19,25,26,28,30^ and myocardial infarction from seven studies (13.0%, 95% CI: 7.0; 23.0, *I*^2^ = 79%).^19–21,25,26,28,30^ Composite adverse clinical events were reported in 16.0% (95% CI: 8.0; 28.0, *I*^2^ = 81%) of patients enrolled in five studies.^19,20,26,28,30^ Any SCAD event (i.e. *de novo*, recurrent, or progressive) was observed in 10.0% (95% CI: 4.0; 22.0, *I*^2^ = 81%) of patients.^19,26,30,32,35^ One study reported a 70.0% healing rate of a SCAD lesion as shown on a planned follow-up coronary angiography.^28^  Supplementary material online, *Table S20* and *Figures S57*–*S61* present clinical outcomes at follow-up. High heterogeneity dominates all pooled variables.

### Sensitivity analyses

Sensitivity analysis according to the study size by including studies of sample size of more than 50 patients^19,24–26,28,30,34^ showed the proportions of patients with hypertension, smoker status, and emotional stress precipitating factors were higher in the studies with large sample size. There lower proportions of patients presented with NSTEMI and who underwent CABG, but more proportions who underwent IVUS and diagnosed with SCAD Type 1 without a difference in the clinical outcomes. Details on the sensitivity analysis and comparison with the overall analysis are presented in Supplementary material online, *Table S21*. Sensitivity analysis according to confirmed SCAD diagnosis by removing the largest study (*n* = 322) that did not clearly include coronary angiographic assessment [34] did not yield different findings compared with the overall analysis (see Supplementary material online, *Table S21*).

## Discussion

This is the first scoping review that mapped studies on SCAD in populations from non-Western countries. It presents the characteristics, management, and outcomes of patients with SCAD (Graphical abstract). This review demonstrates considerable variations between the studies probably due to variations in study design, population, and recruitment period.

The accurate prevalence of SCAD remains uncertain.^42^ The overall pooled prevalence in this review was 1.0% of patients with AMI or those who underwent coronary angiography. The prevalence of SCAD in other international studies from Europe and North America was lower (<1.0; Supplementary material online, *Table S22*)^6,7,9^ than that in most of the studies in this review, which was probably due to their smaller sample size. Mughal *et al*. showed that temporal trends of SCAD incidence were increasing in all the US census regions. The overall crude incidence rose from 4.95 to 14.18 per 1 000 000 discharges per year from 2010 to 2017, respectively.^43^ A national population-based cohort study of 66 360 patients diagnosed with SCAD recorded an increased trend of SCAD diagnosis over time (2004–2015).^44^

When the findings of this review are viewed in the context of international studies and registries from Europe and North America, similarities and differences are anticipated (see Supplementary material online, *Table S28*). More specifically, in comparison with the largest prospective study of 750 patients by Saw *et al*. (see Supplementary material online, *Table S22*),^8,45^ the mean age was similar (52.0 vs. 51.3 years) but more females were reported in the Saw *et al*. study (88.5 vs. 54.0%). A lower proportion of females in studies from non-Western countries might be due to diagnostic inaccuracy with a greater inclusion of atherosclerotic cases (where male patients predominate). Alternatively, this may reflect a regional gender bias in the presentation and diagnosis of SCAD and/or AMI in female patients. Acute myocardial infarction presentation in females may be lower in some countries of the studied regions where females tend to delay seeking medical attention for various reasons including cultural and social factors leading to underdiagnosis and undertreatment.^46–50^ For example, Hadid *et al*. reported the factors that influenced the prehospital delay among women and men who were newly diagnosed with acute coronary syndrome in Jordan. The reasons for the delay included lack of knowledge about the symptoms of the condition; women’s concern about caring for family if they are away; and using alternative therapy to delaying hospitalization, hence, reducing expenses.^46^ Similarly, Wang *et al*.^47^ argued that women in the Chinese culture give a priority for the family over seeking medical treatment for AMI. Chinese women with AMI took longer time to seek medical attention (240 vs. 120 min, *P* = 0.001) and were less likely to use emergency medical service than men. Furthermore, there are other examples of gender bias in access to health care, particularly in patients from lower socioeconomic groups. Rodgers *et al*. investigated the factors preventing older individuals in South-East Asia, namely Cambodia, the Philippines, and Viet Nam, from seeking treatment when sick. The analysis showed that in comparison with men, women in the Philippines and Cambodia were more likely to seek treatment but not in Viet Nam due the discrimination and stigma associated with being sick. However, in the Philippines, although the social health insurance covers 90% of the people, there is a low utilization rate (4.0%), especially among the poor because of other expenses of hospitalization regardless of the insurance coverage. But, in Cambodia, only 20% of the people are covered by the new governmental initiative to provide universal health coverage.^48^ On the other extreme side, Chhabra *et al*. found a gender-based bias among paediatric cardiac patients in treatment-seeking behaviour in North India despite the available free treatment. Only 37.6% of patients were females, and the bias was across all ages. The prevalence of this bias, in both rural and urban areas, reveals a deep-rooted social issue regardless of the financial status. The authors argued that women in North India are subjected to discrimination compared with those in South India.^49^ Ramakrishnan *et al*. found that being a female and of lower socioeconomic classes increased the likelihood to not undergoing paediatric cardiac surgery in India. The surgical scar on a female’s chest, future marital prospects, and the wish to hide the child’s illness from friends and relatives were among the reasons.^50^ Moreover, gender bias has also been reported in terms of nutrition and immunization in India not only cardiac surgery.^49^

The rates of baseline characteristics have varied in terms of dyslipidaemia (20.3 vs. 31.0%), hypertension (32.1 vs. 43.0%), diabetes (4.7 vs. 19.0%), smoking status (11.6 vs. 30.0%), prior myocardial infarction (8.4 vs. 11.0%), and FMD (32.9 vs. 22.0%) between Saw *et al*. prospective study and this review. Physical (38.7 vs. 17.0%) and emotional (50.3 vs. 29.0%) stressors were higher in the study by Saw *et al*.,^8,45^ probably due to their underreporting in this review’s studies. A meta-analysis of 19 studies (*n* = 2172) reported a prevalence of <30.0% for smoking, dyslipidaemia, and diabetes each, but that for hypertension and FMD was as high as 45.0% (95% CI: 35.0; 54.0) and 68.0% (95% CI: 61.0; 74.0), respectively.^51^ Similarly, there were substantial variations in hospital presentation and subsequent management. Saw *et al*. reported STEMI at a lower (29.7 vs. 50.0%) and NSTEMI at higher (69.9 vs. 38.0%) rate than in this review. This may suggest that the patients with SCAD presenting in the non-European regions are at the higher end of the spectrum of disease severity for this condition. This may be because patients with less severe NSTEMI presentations are less likely to seek medical help which was driven by the severity of pain and partially by the cost.^47^ The main documented reasons to delay or ignore seeking medical attending included limited awareness or knowledge about symptoms of AMI^46,52–56^ and living in remote areas.^46,57^ Furthermore, Yu *et al*. reported other reasons such as being young and the related self-confidence that this is not an illness of the young; underestimating the warning signs of AMI; and pressure from social roles.^58^

In the study by Saw *et al*.^45^ the rate of conservative strategy was much higher (84.3 vs. 48.0%), and the rates of PCI (14.1 vs. 42.0%), IVUS use (2.1 vs. 64.0%), and OCT use (5.5 vs. 53.0%) were much lower than in this review. The included studies that reported these variables were small especially those that reported data on IVUS and OCT. Four studies reported OCT use, two of them recruited only patients with OCT use^31,32^ which may have an overestimated the results. It is also acknowledged that SCAD diagnosis is still challenging for many cardiologists.^59^ There were less variations in terms of the distribution of the involved coronary artery and the Saw classification types. The medication distribution upon discharge in the Saw *et al*. study compares well to that reported in the present review. In the DISCO registry (*n* = 314), dual antiplatelet therapy was associated with higher cardiovascular adverse events than single therapy (18.9 vs. 6.0%; hazard ratio 2.62, 95% CI: 1.22; 5.61, *P* = 0.013).^60^ The respondents to Buccheri’s survey considered aspirin, P_2_Y_12_ inhibitors, beta-blockers, and statins as the basic therapy, followed by ACEI, nitrates, calcium channel blockers, and ARB.^61^ There are no randomized trials that delineate optimal SCAD management; hence, it is not possible to assess over- or undertreatment from available literature.^62^ Treatment decision may sometime be individualized according to clinical and angiographic factors.^63^ The ongoing Australian New Zealand Spontaneous Coronary Artery Dissection (ANZ-SCAD) registry has proposed a protocol which comprises five steps: (i) coronary angiography to confirm SCAD diagnosis with additional modalities as appropriate; (ii) conservative management for most cases and intervention (PCI/CABG) for selected patients; (iii) medical therapy includes beta-blockers combined with ACEI/ARB for left ventricular systolic dysfunction and antiplatelets according to SCAD subtype and intervention; (iv) screening for FMD and extracoronary vascular abnormalities with head-to-pelvis non-invasive angiography; and (v) cardiac rehabilitation programme.^64^

Saw *et al*. in their prospective study reported lower all-cause death (0.8 vs. 3.0%), myocardial infarction (9.9 vs. 13.0%), and composite adverse clinical events (14.0 vs. 16.0%) than in the present review at 36 vs. 23 months, respectively.^45^ The outcomes in this review may have been influenced by the delay in seeking medical attention, leading to more severe presentations as discussed above. A meta-analysis reported MACE incidence of 7.80 per 100 person-years (95% CI: 4.50; 13.54).^51^ A pooled analysis found that following a SCAD event, most MACE (67.0%) occurred during the index hospital stay, followed by the discharge to 6-month period (17.0%), and then the 6—12-month period (16.0%) (*P* < 0.0001 for overall comparison), irrespective of management strategy.^65^ Overall, comparing the clinical course of SCAD between the included studies in this review and that reported in studies from Western countries is greatly limited by the high heterogeneity between the included studies.

This scoping review identified gaps in reporting SCAD aspects in non-Western countries. Scoping reviews have limitations because they focus on the breadth at the expense of the information depth. Consequently, although in-depth data analysis is not usually required in scoping reviews,^12^ a systematic inquiry was conducted with data synthesis into various themes. However, all the identified studies were of observational design which is prone to bias and confounding, including selection, attrition, and ascertainment bias. The studies were of small sample size and short follow-up. The retrospective analysis and small sample size may have affected the preciseness in estimating SCAD prevalence and outcomes. There was high heterogeneity among the studies as indicated by the inconsistency factor (i.e. *I*^2^ > 50%) of the supplementary forest plots, due to differences in geographical regions, genetic predisposition, cultural and socioeconomic considerations, ethnic diversity, medical services, clinical practice, income levels, and so forth. Apart from Australia, China, and Japan, evidence is inadequate or lacking in other pre-specified countries. Thus, more homogenous studies of prospective data are needed to further characterize patients with SCAD, management, long-term outcomes, prognosis, ethnic and racial difference, genetics, and economic aspects. Each of the aforementioned point can be a good hypothesis-generating question for future research. Currently, several registries are ongoing such as the ANZ-SCAD (ACTRN12621000824864),^64^ the EURObservational Research Programme SCAD,^66^ the International Spontaneous Coronary Artery Dissection (iSCAD; NCT04496687), the ‘Virtual’ Multicenter SCAD (NCT01429727), the Spontaneous Coronary Artery Dissection National Swiss (SwissSCAD; NCT04457544), and the Spontaneous Coronary Artery Dissection anaLysIs of the Brazilian Updated Registry (NCT03398850). The iSCAD registry (NCT04496687) has published several preliminary analyses.^67–72^ Lastly, the BA-SCAD (beta-blockers and antiplatelet agents in patients with SCAD; NCT04850417) randomized trial aims to assess beta-blockers and antiplatelet therapy in SCAD,^73^ while the Statin and Angiotensin-converting Enzyme Inhibitor on Symptoms in Patients with SCAD (SAFER-SCAD; NCT02008786) randomized trial was terminated due to slow enrolment.

## Conclusions

Available evidence on SCAD from the non-Western countries is limited to observational studies. There is noticeable variability and heterogeneity in the clinical course of SCAD between the included studies and when compared with that reported from Western countries. This review identified knowledge gaps about the global burden of SCAD in non-Western countries that should be addressed in future prospective population-based studies.

## Supplementary Material

oeaf022_Supplementary_Data

## Data Availability

No new data were generated or analysed in support of this research.
