# Supplementary material for: Spontaneous coronary artery dissection in regions of non-Western populations: a systematic literature search and scoping review
Source: Eur Heart J Open. 2025 Mar 19;5(2):oeaf022. doi: 10.1093/ehjopen/oeaf022 (PMC11986324; doi:10.1093/ehjopen/oeaf022)
Supplement: oeaf022_Supplementary_Data [file oeaf022_supplementary_data.pdf]

Spontaneous coronary artery dissection in regions of non-Western populations: a systematic literature search and scoping review

Table of content

Supplementary tables

Table S1. Literature search strategy and results (Set 1)
Table S2. Literature search update and results (Set 2)
Table S3. Excluded studies
Table S4. Studies from Australia, New Zealand, and Cambodia
Table S5. Study objective, conclusion, diagnosis, and exclusion criteria
Table S6. SCAD classification by included studies
Table S7. General study characteristics
Table S8. Demographics and risk factors
Table S9. Co-morbidities at baseline
Table S10. Predisposing conditions and precipitating factors or preceding events
Tables S11. Female sex related characteristics
Table S12. Clinical manifestation and presentation upon hospital admission
Table S13. Laboratory findings
Table S14. Echocardiography parameters and FMD screening
Table S15. In-hospital SCAD management
Table S16. Percutaneous procedure characteristics
Table S17. Angiographic characteristics of SCAD lesion(s) – 3 Parts
Table S18. Medications at discharge
Table S19. Post-procedure or in-hospital outcomes
Table S20. Clinical outcomes at follow-up
Table S21. Sensitivity analyses according to study size and confirmed SCAD diagnosis (Tables S21.1. – S21.13.)
Table S22. Patient characteristics and outcomes in international registries and nationwide studies

Supplementary figures

Figure S1. PRISMA flow diagram

Single proportion meta-analysis

Figure S2. Pooled proportions of prevalence for all studies that reported it
Figure S3. Pooled proportions of prevalence without studies recruited only females
Figure S4. Pooled proportions of prevalence for studies that recruited only females
Figure S5. Pooled means of age
Figure S6. Pooled proportions of female variable (excluding studies that recruited only females)
Figure S7. Pooled proportions of dyslipidemia
Figure S8. Pooled proportions of hypertension
Figure S9. Pooled proportions of diabetes
Figure S10. Pooled proportions of smokers
Figure S11. Pooled proportions of history of prior myocardial infarction
Figure S12. Pooled proportions of history of fibromuscular dysplasia
Figure S13. Pooled proportions of exposure to physical stress
Figure S14. Pooled proportions of exposure to emotional stress
Figure S15. Pooled proportions of females in menopause
Figure S16. Pooled proportions of females in menopause without Nakashima et al study (early menopause)
Figure S17. Pooled proportions of ST-segment elevation myocardial infarction
Figure S18. Pooled proportions of non-ST-segment elevation myocardial infarction
Figure S19. Pooled proportions of unstable angina
Figure S20. Pooled means of left ventricular ejection fraction
Figure S21. Pooled proportions of conservative revascularization management
Figure S22. Pooled proportions of undergoing percutaneous coronary artery intervention
Figure S23. Pooled proportions of undergoing balloon angioplasty

Figure S24. Pooled proportions of undergoing intravascular ultrasound
Figure S25. Pooled proportions of undergoing optical coherence tomography
Figure S26. Pooled proportions of undergoing coronary artery bypass grafting
Figure S27. Pooled proportions of one-lesion SCAD
Figure S28. Pooled proportions of two-lesion SCAD
Figure S29. Pooled proportions of multivessel SCAD
Figure S30. Pooled proportions of left anterior descending artery involvement
Figure S31. Pooled proportions of right coronary artery involvement
Figure S32. Pooled proportions of left circumflex artery involvement
Figure S33. Pooled proportions of left main coronary artery involvement
Figure S34. Pooled proportions of coronary arteries branches involvement
Figure S35. Pooled proportions of distal segments involvement
Figure S36. Pooled proportions of middle segments involvement
Figure S37. Pooled proportions of proximal segments involvement
Figure S38. Pooled proportions of Type 1 SCAD
Figure S39. Pooled proportions of Type 2 SCAD
Figure S40. Pooled proportions of Type 3 SCAD
Figure S41. Pooled proportions of initial TIMI flow grade 0 or I
Figure S42. Pooled proportions of initial TIMI flow grade II or III
Figure S43. Pooled means of percentage of stenosis severity
Figure S44. Pooled means SCAD lesion length (mm)
Figure S45. Pooled proportions of aspirin use
Figure S46. Pooled proportions of P_2Y_{12} inhibitors use
Figure S47. Pooled proportions of single antiplatelet therapy use
Figure S48. Pooled proportions of dual antiplatelet use
Figure S49. Pooled proportions of beta-blockers use
Figure S50. Pooled proportions of renin-angiotensin-aldosterone system inhibitors use
Figure S51. Pooled proportions of calcium channel blockers use
Figure S52. Pooled proportions of statin therapy use
Figure S53. Pooled proportions of PCI-related complications (e.g., stent thrombosis, iatrogenic dissection)
Figure S54. Pooled proportions of in-hospital death
Figure S55. Pooled proportions of any in-hospital cardiovascular event
Figure S56. Pooled proportions of extension of dissection

Figure S57. Pooled means of follow-up durations
Figure S58. Pooled proportions of all-cause death
Figure S59. Pooled proportions of myocardial infarction events
Figure S60. Pooled proportions of any cardiovascular event
Figure S61. Pooled proportions of any SCAD event (de novo, recurrent, progressive)

Tables

Table S1. Literature search strategy and results (Set 1)

	Literature search strategy	
Regions	WHO regions	
	Eastern Mediterranean region<br>Afghanistan, Bahrain, Djibouti, Egypt, Iran, Iraq, Jordan, Kuwait, Lebanon, Libya, Morocco, Oman, Pakistan, Palestine, Qatar, Saudi Arabia, Somalia, Sudan, Syria, Tunisia, United Arab Emirates, and Yemen.	
	South-East Asia region<br>Bangladesh, Bhutan, India, Indonesia, Maldives, Myanmar, Nepal, North Korea, Sri Lanka, Thailand, Timor, and Timor-Leste.	
	Western Pacific Region<br>Australia, Brunei, Cambodia, China, Cook Islands, Fiji, Japan, Kiribati, Laos, Malaysia, Marshall Islands, Micronesia, Mongolia, Nauru, New Zealand, Niue, Palau, Papua New Guinea, Philippines, Samoa, Singapore, Solomon Islands, South Korea, Taiwan, Tonga, Tuvalu, Vanuatu, and Vietnam.	
Limits	No search limits	
EMBASE	Searches and terms - December 28, 2023 (OVID® interface)	
1	scad.ab,dm,dv,fx,hw,kf,mf,ot,ti,tn,dq.	2860
2	spontaneous coronary artery dissection.ab,dm,dv,fx,hw,kf,mf,ot,ti,tn,dq.	2559
3	(scad or spontaneous coronary artery dissection).ab,dm,dv,fx,hw,kf,mf,ot,ti,tn,dq.	3953
4	asia.ab,dm,dv,fx,hw,kf,mf,ot,ti,tn,dq.	170419
5	bangladesh.ab,dm,dv,fx,hw,kf,mf,ot,ti,tn,dq.	27499
6	bhutan.ab,dm,dv,fx,hw,kf,mf,ot,ti,tn,dq.	1523
7	india.ab,dm,dv,fx,hw,kf,mf,ot,ti,tn,dq.	280824
8	indonesia.ab,dm,dv,fx,hw,kf,mf,ot,ti,tn,dq.	35814
9	maldives.ab,dm,dv,fx,hw,kf,mf,ot,ti,tn,dq.	725
10	myanmar.ab,dm,dv,fx,hw,kf,mf,ot,ti,tn,dq.	6662
11	nepal.ab,dm,dv,fx,hw,kf,mf,ot,ti,tn,dq.	19344
12	sri lanka.ab,dm,dv,fx,hw,kf,mf,ot,ti,tn,dq.	13172
13	thailand.ab,dm,dv,fx,hw,kf,mf,ot,ti,tn,dq.	52863

14	timor.ab,dm,dv,fx,hw,kf,mf,ot,ti,tn,dq.	1178
15	timor-leste.ab,dm,dv,fx,hw,kf,mf,ot,ti,tn,dq.	926
16	north korea.ab,dm,dv,fx,hw,kf,mf,ot,ti,tn,dq.	2224
17	(asia or bangladesh or bhutan or india or indonesia or maldives or myanmar or nepal or sri lanka or thailand or timor or timor-leste or north korea).ab,dm,dv,fx,hw,kf,mf,ot,ti,tn,dq.	563718
18	Mediterranean.ab,dm,dv,fx,hw,kf,mf,ot,ti,tn,dq.	66839
19	afghanistan.ab,dm,dv,fx,hw,kf,mf,ot,ti,tn,dq.	10628
20	bahrain.ab,dm,dv,fx,hw,kf,mf,ot,ti,tn,dq.	2552
21	djibouti.ab,dm,dv,fx,hw,kf,mf,ot,ti,tn,dq.	665
22	egypt.ab,dm,dv,fx,hw,kf,mf,ot,ti,tn,dq.	37519
23	iran.ab,dm,dv,fx,hw,kf,mf,ot,ti,tn,dq.	92604
24	iraq.ab,dm,dv,fx,hw,kf,mf,ot,ti,tn,dq.	18591
25	jordan.ab,dm,dv,fx,hw,kf,mf,ot,ti,tn,dq.	13670
26	kuwait.ab,dm,dv,fx,hw,kf,mf,ot,ti,tn,dq.	7475
27	lebanon.ab,dm,dv,fx,hw,kf,mf,ot,ti,tn,dq.	9457
28	libya.ab,dm,dv,fx,hw,kf,mf,ot,ti,tn,dq.	1927
29	morocco.ab,dm,dv,fx,hw,kf,mf,ot,ti,tn,dq.	12430
30	oman.ab,dm,dv,fx,hw,kf,mf,ot,ti,tn,dq.	5318
31	pakistan.ab,dm,dv,fx,hw,kf,mf,ot,ti,tn,dq.	48623
32	palestine.ab,dm,dv,fx,hw,kf,mf,ot,ti,tn,dq.	3243
33	qatar.ab,dm,dv,fx,hw,kf,mf,ot,ti,tn,dq.	5785
34	saudi arabia.ab,dm,dv,fx,hw,kf,mf,ot,ti,tn,dq.	37524
35	somalia.ab,dm,dv,fx,hw,kf,mf,ot,ti,tn,dq.	3138
36	sudan.ab,dm,dv,fx,hw,kf,mf,ot,ti,tn,dq.	13237
37	syria.ab,dm,dv,fx,hw,kf,mf,ot,ti,tn,dq.	3485
38	tunisia.ab,dm,dv,fx,hw,kf,mf,ot,ti,tn,dq.	15429
39	united arab emirates.ab,dm,dv,fx,hw,kf,mf,ot,ti,tn,dq.	6468
40	yemen.ab,dm,dv,fx,hw,kf,mf,ot,ti,tn,dq.	3237
41	(Mediterranean or afghanistan or bahrain or djibouti or egypt or iran or iraq or jordan or kuwait or lebanon or libya or morocco or oman or pakistan or palestine or qatar or saudi arabia or somalia or sudan or syria or tunisia or united arab emirates or yemen).ab,dm,dv,fx,hw,kf,mf,ot,ti,tn,dq.	384371
42	Pacific.ab,dm,dv,fx,hw,kf,mf,ot,ti,tn,dq.	67995

43	australia.ab,dm,dv,fx,hw,kf,mf,ot,ti,tn,dq.	270242
44	brunei.ab,dm,dv,fx,hw,kf,mf,ot,ti,tn,dq.	923
45	cambodia.ab,dm,dv,fx,hw,kf,mf,ot,ti,tn,dq.	7465
46	china.ab,dm,dv,fx,hw,kf,mf,ot,ti,tn,dq.	519372
47	cook islands.ab,dm,dv,fx,hw,kf,mf,ot,ti,tn,dq.	301
48	fiji.ab,dm,dv,fx,hw,kf,mf,ot,ti,tn,dq.	3776
49	japan.ab,dm,dv,fx,hw,kf,mf,ot,ti,tn,dq.	494978
50	kiribati.ab,dm,dv,fx,hw,kf,mf,ot,ti,tn,dq.	315
51	laos.ab,dm,dv,fx,hw,kf,mf,ot,ti,tn,dq.	3932
52	malaysia.ab,dm,dv,fx,hw,kf,mf,ot,ti,tn,dq.	37453
53	marshall islands.ab,dm,dv,fx,hw,kf,mf,ot,ti,tn,dq.	431
54	micronesia.ab,dm,dv,fx,hw,kf,mf,ot,ti,tn,dq.	1393
55	mongolia.ab,dm,dv,fx,hw,kf,mf,ot,ti,tn,dq.	7891
56	nauru.ab,dm,dv,fx,hw,kf,mf,ot,ti,tn,dq.	231
57	new zealand.ab,dm,dv,fx,hw,kf,mf,ot,ti,tn,dq.	129014
58	niue.ab,dm,dv,fx,hw,kf,mf,ot,ti,tn,dq.	115
59	palau.ab,dm,dv,fx,hw,kf,mf,ot,ti,tn,dq.	618
60	papua new guinea.ab,dm,dv,fx,hw,kf,mf,ot,ti,tn,dq.	7443
61	philippines.ab,dm,dv,fx,hw,kf,mf,ot,ti,tn,dq.	17690
62	samoa.ab,dm,dv,fx,hw,kf,mf,ot,ti,tn,dq.	1488
63	singapore.ab,dm,dv,fx,hw,kf,mf,ot,ti,tn,dq.	38090
64	solomon islands.ab,dm,dv,fx,hw,kf,mf,ot,ti,tn,dq.	1159
65	south korea.ab,dm,dv,fx,hw,kf,mf,ot,ti,tn,dq.	61649
66	taiwan.ab,dm,dv,fx,hw,kf,mf,ot,ti,tn,dq.	81940
67	tonga.ab,dm,dv,fx,hw,kf,mf,ot,ti,tn,dq.	722
68	tuvalu.ab,dm,dv,fx,hw,kf,mf,ot,ti,tn,dq.	119
69	vanuatu.ab,dm,dv,fx,hw,kf,mf,ot,ti,tn,dq.	947
70	vietnam.ab,dm,dv,fx,hw,kf,mf,ot,ti,tn,dq.	22499
71	(Pacific or australia or brunei or cambodia or china or cook islands or fiji or japan or kiribati or laos or malaysia or marshall islands or micronesia or mongolia or nauru or new zealand or niue or palau or papua new guinea or philippines or samoa or singapore or solomon islands or south korea or taiwan or tonga or tuvalu or vanuatu or vietnam).ab,dm,dv,fx,hw,kf,mf,ot,ti,tn,dq.	1625688

72	((scad or spontaneous coronary artery dissection) and (asia or bangladesh or bhutan or india or indonesia or maldives or myanmar or nepal or sri lanka or thailand or timor or timor-leste or north korea)).ab,dm,dv,fx,hw,kf,mf,ot,ti,tn,dq.	43
73	((scad or spontaneous coronary artery dissection) and (Mediterranean or afghanistan or bahrain or djibouti or egypt or iran or iraq or jordan or kuwait or lebanon or libya or morocco or oman or pakistan or palestine or qatar or saudi arabia or somalia or sudan or syria or tunisia or united arab emirates or yemen)).ab,dm,dv,fx,hw,kf,mf,ot,ti,tn,dq.	32
74	((scad or spontaneous coronary artery dissection) and (Pacific or australia or brunei or cambodia or china or cook islands or fiji or japan or kiribati or laos or malaysia or marshall islands or micronesia or mongolia or nauru or new zealand or niue or palau or papua new guinea or philippines or samoa or singapore or solomon islands or south korea or taiwan or tonga or tuvalu or vanuatu or vietnam)).ab,dm,dv,fx,hw,kf,mf,ot,ti,tn,dq.	150
75	(((scad or spontaneous coronary artery dissection) and (asia or bangladesh or bhutan or india or indonesia or maldives or myanmar or nepal or sri lanka or thailand or timor or timor-leste or north korea)) or ((scad or spontaneous coronary artery dissection) and (Mediterranean or afghanistan or bahrain or djibouti or egypt or iran or iraq or jordan or kuwait or lebanon or libya or morocco or oman or pakistan or palestine or qatar or saudi arabia or somalia or sudan or syria or tunisia or united arab emirates or yemen)) or ((scad or spontaneous coronary artery dissection) and (Pacific or australia or brunei or cambodia or china or cook islands or fiji or japan or kiribati or laos or malaysia or marshall islands or micronesia or mongolia or nauru or new zealand or niue or palau or papua new guinea or philippines or samoa or singapore or solomon islands or south korea or taiwan or tonga or tuvalu or vanuatu or vietnam))).ab,dm,dv,fx,hw,kf,mf,ot,ti,tn,dq.	**217**

Medline	Searches and terms - December 28, 2023 (OVID® interface)	Results
1	scad.af.	1717
2	spontaneous coronary artery dissection.af.	1686
3	(scad or spontaneous coronary artery dissection).ab,bt,hw,kf,ot,sy,ti,fx,mx,nm,ox,px,rx,ui,ux.	2563
4	south-east asia.ab,bt,hw,kf,ot,sy,ti,fx,mx,nm,ox,px,rx,ui,ux.	4762
5	bangladesh.ab,bt,hw,kf,ot,sy,ti,fx,mx,nm,ox,px,rx,ui,ux.	22545
6	bhutan.ab,bt,hw,kf,ot,sy,ti,fx,mx,nm,ox,px,rx,ui,ux.	1315
7	north korea.ab,bt,hw,kf,ot,sy,ti,fx,mx,nm,ox,px,rx,ui,ux.	476
8	india.ab,bt,hw,kf,ot,sy,ti,fx,mx,nm,ox,px,rx,ui,ux.	188089
9	indonesia.ab,bt,hw,kf,ot,sy,ti,fx,mx,nm,ox,px,rx,ui,ux.	23770
10	maldives.ab,bt,hw,kf,ot,sy,ti,fx,mx,nm,ox,px,rx,ui,ux.	526
11	myanmar.ab,bt,hw,kf,ot,sy,ti,fx,mx,nm,ox,px,rx,ui,ux.	5796

12	nepal.ab,bt,hw,kf,ot,sy,ti,fx,mx,nm,ox,px,rx,ui,ux.	16192
13	sri lanka.ab,bt,hw,kf,ot,sy,ti,fx,mx,nm,ox,px,rx,ui,ux.	10715
14	thailand.ab,bt,hw,kf,ot,sy,ti,fx,mx,nm,ox,px,rx,ui,ux.	45157
15	timor.ab,bt,hw,kf,ot,sy,ti,fx,mx,nm,ox,px,rx,ui,ux.	895
16	timor-leste.ab,bt,hw,kf,ot,sy,ti,fx,mx,nm,ox,px,rx,ui,ux.	568
17	(south-east asia or bangladesh or bhutan or north korea or india or indonesia or maldives or myanmar or nepal or sri lanka or thailand or timor or timor-leste).ab,bt,hw,kf,ot,sy,ti,fx,mx,nm,ox,px,rx,ui,ux.	302596
18	Mediterranean.ab,bt,hw,kf,ot,sy,ti,fx,mx,nm,ox,px,rx,ui,ux.	55545
19	afghanistan.ab,bt,hw,kf,ot,sy,ti,fx,mx,nm,ox,px,rx,ui,ux.	8410
20	bahrain.ab,bt,hw,kf,ot,sy,ti,fx,mx,nm,ox,px,rx,ui,ux.	1515
21	djibouti.ab,bt,hw,kf,ot,sy,ti,fx,mx,nm,ox,px,rx,ui,ux.	519
22	egypt.ab,bt,hw,kf,ot,sy,ti,fx,mx,nm,ox,px,rx,ui,ux.	27569
23	iran.ab,bt,hw,kf,ot,sy,ti,fx,mx,nm,ox,px,rx,ui,ux.	68603
24	iraq.ab,bt,hw,kf,ot,sy,ti,fx,mx,nm,ox,px,rx,ui,ux.	12644
25	jordan.ab,bt,hw,kf,ot,sy,ti,fx,mx,nm,ox,px,rx,ui,ux.	10293
26	kuwait.ab,bt,hw,kf,ot,sy,ti,fx,mx,nm,ox,px,rx,ui,ux.	5336
27	lebanon.ab,bt,hw,kf,ot,sy,ti,fx,mx,nm,ox,px,rx,ui,ux.	8167
28	libya.ab,bt,hw,kf,ot,sy,ti,fx,mx,nm,ox,px,rx,ui,ux.	2048
29	morocco.ab,bt,hw,kf,ot,sy,ti,fx,mx,nm,ox,px,rx,ui,ux.	10168
30	oman.ab,bt,hw,kf,ot,sy,ti,fx,mx,nm,ox,px,rx,ui,ux.	4494
31	pakistan.ab,bt,hw,kf,ot,sy,ti,fx,mx,nm,ox,px,rx,ui,ux.	35596
32	palestine.ab,bt,hw,kf,ot,sy,ti,fx,mx,nm,ox,px,rx,ui,ux.	2223
33	qatar.ab,bt,hw,kf,ot,sy,ti,fx,mx,nm,ox,px,rx,ui,ux.	3741
34	saudi arabia.ab,bt,hw,kf,ot,sy,ti,fx,mx,nm,ox,px,rx,ui,ux.	32865
35	somalia.ab,bt,hw,kf,ot,sy,ti,fx,mx,nm,ox,px,rx,ui,ux.	2962
36	sudan.ab,bt,hw,kf,ot,sy,ti,fx,mx,nm,ox,px,rx,ui,ux.	11407
37	syria.ab,bt,hw,kf,ot,sy,ti,fx,mx,nm,ox,px,rx,ui,ux.	4506
38	tunisia.ab,bt,hw,kf,ot,sy,ti,fx,mx,nm,ox,px,rx,ui,ux.	12275
39	united arab emirates.ab,bt,hw,kf,ot,sy,ti,fx,mx,nm,ox,px,rx,ui,ux.	4711
40	yemen.ab,bt,hw,kf,ot,sy,ti,fx,mx,nm,ox,px,rx,ui,ux.	2788

41	(Mediterranean or afghanistan or bahrain or djibouti or egypt or iran or iraq or jordan or kuwait or lebanon or libya or morocco or oman or pakistan or palestine or qatar or saudi arabia or somalia or sudan or syria or tunisia or united arab emirates or yemen).ab,bt,hw,kf,ot,sy,ti,fx,mx,nm,ox,px,rx,ui,ux.	302938
42	pacific.ab,bt,hw,kf,ot,sy,ti,fx,mx,nm,ox,px,rx,ui,ux.	60583
43	australia.ab,bt,hw,kf,ot,sy,ti,fx,mx,nm,ox,px,rx,ui,ux.	199907
44	brunei.ab,bt,hw,kf,ot,sy,ti,fx,mx,nm,ox,px,rx,ui,ux.	676
45	cambodia.ab,bt,hw,kf,ot,sy,ti,fx,mx,nm,ox,px,rx,ui,ux.	5934
46	china.ab,bt,hw,kf,ot,sy,ti,fx,mx,nm,ox,px,rx,ui,ux.	402509
47	cook islands.ab,bt,hw,kf,ot,sy,ti,fx,mx,nm,ox,px,rx,ui,ux.	254
48	fiji.ab,bt,hw,kf,ot,sy,ti,fx,mx,nm,ox,px,rx,ui,ux.	2663
49	japan.ab,bt,hw,kf,ot,sy,ti,fx,mx,nm,ox,px,rx,ui,ux.	230572
50	kiribati.ab,bt,hw,kf,ot,sy,ti,fx,mx,nm,ox,px,rx,ui,ux.	264
51	laos.ab,bt,hw,kf,ot,sy,ti,fx,mx,nm,ox,px,rx,ui,ux.	3699
52	malaysia.ab,bt,hw,kf,ot,sy,ti,fx,mx,nm,ox,px,rx,ui,ux.	28602
53	marshall islands.ab,bt,hw,kf,ot,sy,ti,fx,mx,nm,ox,px,rx,ui,ux.	360
54	micronesia.ab,bt,hw,kf,ot,sy,ti,fx,mx,nm,ox,px,rx,ui,ux.	1828
55	mongolia.ab,bt,hw,kf,ot,sy,ti,fx,mx,nm,ox,px,rx,ui,ux.	6551
56	nauru.ab,bt,hw,kf,ot,sy,ti,fx,mx,nm,ox,px,rx,ui,ux.	174
57	new zealand.ab,bt,hw,kf,ot,sy,ti,fx,mx,nm,ox,px,rx,ui,ux.	83541
58	niue.ab,bt,hw,kf,ot,sy,ti,fx,mx,nm,ox,px,rx,ui,ux.	86
59	palau.ab,bt,hw,kf,ot,sy,ti,fx,mx,nm,ox,px,rx,ui,ux.	530
60	papua new guinea.ab,bt,hw,kf,ot,sy,ti,fx,mx,nm,ox,px,rx,ui,ux.	5922
61	philippines.ab,bt,hw,kf,ot,sy,ti,fx,mx,nm,ox,px,rx,ui,ux.	14955
62	samoa.ab,bt,hw,kf,ot,sy,ti,fx,mx,nm,ox,px,rx,ui,ux.	1387
63	singapore.ab,bt,hw,kf,ot,sy,ti,fx,mx,nm,ox,px,rx,ui,ux.	24253
64	solomon islands.ab,bt,hw,kf,ot,sy,ti,fx,mx,nm,ox,px,rx,ui,ux.	986
65	south korea.ab,bt,hw,kf,ot,sy,ti,fx,mx,nm,ox,px,rx,ui,ux.	19724
66	taiwan.ab,bt,hw,kf,ot,sy,ti,fx,mx,nm,ox,px,rx,ui,ux.	64613
67	tonga.ab,bt,hw,kf,ot,sy,ti,fx,mx,nm,ox,px,rx,ui,ux.	654
68	tuvalu.ab,bt,hw,kf,ot,sy,ti,fx,mx,nm,ox,px,rx,ui,ux.	90
69	vanuatu.ab,bt,hw,kf,ot,sy,ti,fx,mx,nm,ox,px,rx,ui,ux.	868
70	vietnam.ab,bt,hw,kf,ot,sy,ti,fx,mx,nm,ox,px,rx,ui,ux.	24319

71	(pacific or australia or brunei or cambodia or china or cook islands or fiji or japan or kiribati or laos or malaysia or marshall islands or micronesia or mongolia or nauru or new zealand or niue or palau or papua new guinea or philippines or samoa or singapore or solomon islands or south korea or taiwan or tonga or tuvalu or vanuatu or vietnam).ab,bt,hw,kf,ot,sy,ti,fx,mx,nm,ox,px,rx,ui,ux.	1094056
72	((scad or spontaneous coronary artery dissection) and (south-east asia or bangladesh or bhutan or north korea or india or indonesia or maldives or myanmar or nepal or sri lanka or thailand or timor or timor-leste)).ab,bt,hw,kf,ot,sy,ti,fx,mx,nm,ox,px,rx,ui,ux.	11
73	((scad or spontaneous coronary artery dissection) and (Mediterranean or afghanistan or bahrain or djibouti or egypt or iran or iraq or jordan or kuwait or lebanon or libya or morocco or oman or pakistan or palestine or qatar or saudi arabia or somalia or sudan or syria or tunisia or united arab emirates or yemen)).ab,bt,hw,kf,ot,sy,ti,fx,mx,nm,ox,px,rx,ui,ux.	21
74	((scad or spontaneous coronary artery dissection) and (pacific or australia or brunei or cambodia or china or cook islands or fiji or japan or kiribati or laos or malaysia or marshall islands or micronesia or mongolia or nauru or new zealand or niue or palau or papua new guinea or philippines or samoa or singapore or solomon islands or south korea or taiwan or tonga or tuvalu or vanuatu or vietnam)).ab,bt,hw,kf,ot,sy,ti,fx,mx,nm,ox,px,rx,ui,ux.	85
75	(((scad or spontaneous coronary artery dissection) and (south-east asia or bangladesh or bhutan or north korea or india or indonesia or maldives or myanmar or nepal or sri lanka or thailand or timor or timor-leste)) or ((scad or spontaneous coronary artery dissection) and (Mediterranean or afghanistan or bahrain or djibouti or egypt or iran or iraq or jordan or kuwait or lebanon or libya or morocco or oman or pakistan or palestine or qatar or saudi arabia or somalia or sudan or syria or tunisia or united arab emirates or yemen)) or ((scad or spontaneous coronary artery dissection) and (pacific or australia or brunei or cambodia or china or cook islands or fiji or japan or kiribati or laos or malaysia or marshall islands or micronesia or mongolia or nauru or new zealand or niue or palau or papua new guinea or philippines or samoa or singapore or solomon islands or south korea or taiwan or tonga or tuvalu or vanuatu or vietnam))).ab,bt,hw,kf,ot,sy,ti,fx,mx,nm,ox,px,	**111**

Table S2. Literature search update and results (Set 2)

Medline	Searches and terms - April 24, 2024	Results
	Same WHO regions as above with the addition of Turkey as a country	
Limits	No search limits	
1	“SCAD” AND “Turkey”	38
	"Spontaneous Coronary Artery Dissection" OR "SCAD" AND "Turkey"	59
	("Coronary Artery Dissection, Spontaneous" [Supplementary Concept]) AND "Turkey"[Mesh]	0
2	“SCAD” AND “Bangladesh”	2
	"Spontaneous Coronary Artery Dissection" OR "SCAD" AND "Turkey"	2
	("Coronary Artery Dissection, Spontaneous" [Supplementary Concept]) AND "Bangladesh"[Mesh]	0
3	“SCAD” AND “Bhutan”	0
	"Spontaneous Coronary Artery Dissection" OR "SCAD" AND "Bhutan"	0
	("Coronary Artery Dissection, Spontaneous" [Supplementary Concept]) AND "Bhutan"[Mesh]	0
4	“SCAD” AND “India”	41
	"Spontaneous Coronary Artery Dissection" OR "SCAD" AND "India"	62
	("Coronary Artery Dissection, Spontaneous" [Supplementary Concept]) AND "India"[Mesh]	0
5	“SCAD” AND “Indonesia”	13
	"Spontaneous Coronary Artery Dissection" OR "SCAD" AND "Indonesia"	13
	("Coronary Artery Dissection, Spontaneous" [Supplementary Concept]) AND "Indonesia"[Mesh]	0
6	“SCAD” AND “Maldives”	1
	"Spontaneous Coronary Artery Dissection" OR "SCAD" AND " Maldives"	1
	("Coronary Artery Dissection, Spontaneous" [Supplementary Concept]) AND "Maldives"[Mesh]	0
7	“SCAD” AND “Myanmar”	0
	"Spontaneous Coronary Artery Dissection" OR "SCAD" AND "Myanmar"	0
	("Coronary Artery Dissection, Spontaneous" [Supplementary Concept]) AND "Myanmar"[Mesh]	0
8	“SCAD” AND “Nepal”	4
	"Spontaneous Coronary Artery Dissection" OR "SCAD" AND "Nepal"	5
	("Coronary Artery Dissection, Spontaneous" [Supplementary Concept]) AND "Nepal"[Mesh]	
9	“SCAD” AND “Sri Lanka”	1

	"Spontaneous Coronary Artery Dissection" OR "SCAD" AND "Sri Lanka"	1
	("Coronary Artery Dissection, Spontaneous" [Supplementary Concept]) AND "Sri Lanka"[Mesh]	0
10	“SCAD” AND “Thailand”	6
	"Spontaneous Coronary Artery Dissection" OR "SCAD" AND "Thailand"	6
	("Coronary Artery Dissection, Spontaneous" [Supplementary Concept]) AND "Thailand"[Mesh]	0
11	“SCAD” AND “North Korea”	0
	"Spontaneous Coronary Artery Dissection" OR "SCAD" AND " North Korea"	0
	("Coronary Artery Dissection, Spontaneous" [Supplementary Concept]) AND "Democratic People's Republic of Korea"[Mesh]	0
12	“SCAD” AND “Timor”	0
	"Spontaneous Coronary Artery Dissection" OR "SCAD" AND "Timor"	0
	("Coronary Artery Dissection, Spontaneous" [Supplementary Concept]) AND "Timor-Leste"[Mesh]	0
13	“SCAD” AND “Afghanistan”	0
	"Spontaneous Coronary Artery Dissection" OR "SCAD" AND "Afghanistan"	0
	("Coronary Artery Dissection, Spontaneous" [Supplementary Concept]) AND "Afghanistan"[Mesh]	0
14	“SCAD” AND “Bahrain”	6
	"Spontaneous Coronary Artery Dissection" OR "SCAD" AND "Bahrain”	6
	("Coronary Artery Dissection, Spontaneous" [Supplementary Concept]) AND "Bahrain"[Mesh]	0
15	“SCAD” AND “Djibouti”	0
	"Spontaneous Coronary Artery Dissection" OR "SCAD" AND "Djibouti”	0
	("Coronary Artery Dissection, Spontaneous" [Supplementary Concept]) AND "Djibouti"[Mesh]	0
16	“SCAD” AND “Egypt”	15
	"Spontaneous Coronary Artery Dissection" OR "SCAD" AND “Egypt”	17
	("Coronary Artery Dissection, Spontaneous" [Supplementary Concept]) AND "Egypt"[Mesh]	0
17	“SCAD” AND “Iran”	26
	"Spontaneous Coronary Artery Dissection" OR "SCAD" AND “Iran”	31
	("Coronary Artery Dissection, Spontaneous" [Supplementary Concept]) AND "Iran"[Mesh]	0
18	“SCAD” AND “Iraq”	5
	"Spontaneous Coronary Artery Dissection" OR "SCAD" AND “Iraq”	5
	("Coronary Artery Dissection, Spontaneous" [Supplementary Concept]) AND "Iraq"[Mesh]	0
19	“SCAD” AND “Jordan”	3
	"Spontaneous Coronary Artery Dissection" OR "SCAD" AND “Jordan”	4

	("Coronary Artery Dissection, Spontaneous" [Supplementary Concept]) AND "Jordan"[Mesh]	0
20	“SCAD” AND “Kuwait”	6
	"Spontaneous Coronary Artery Dissection" OR "SCAD" AND “Kuwait”	6
	("Coronary Artery Dissection, Spontaneous" [Supplementary Concept]) AND "Kuwait"[Mesh]	0
21	“SCAD” AND “Lebanon”	12
	"Spontaneous Coronary Artery Dissection" OR "SCAD" AND “Lebanon”	14
	("Coronary Artery Dissection, Spontaneous" [Supplementary Concept]) AND "Lebanon"[Mesh]	0
22	“SCAD” AND “Libya”	0
	"Spontaneous Coronary Artery Dissection" OR "SCAD" AND “Libya”	0
	("Coronary Artery Dissection, Spontaneous" [Supplementary Concept]) AND "Libya"[Mesh]	0
23	“SCAD” AND “Morocco”	4
	"Spontaneous Coronary Artery Dissection" OR "SCAD" AND “Morocco”	5
	("Coronary Artery Dissection, Spontaneous" [Supplementary Concept]) AND "Morocco"[Mesh]	0
24	“SCAD” AND “Oman”	4
	"Spontaneous Coronary Artery Dissection" OR "SCAD" AND “Oman”	5
	("Coronary Artery Dissection, Spontaneous" [Supplementary Concept]) AND "Oman"[Mesh]	0
25	“SCAD” AND “Pakistan”	9
	"Spontaneous Coronary Artery Dissection" OR "SCAD" AND “Pakistan”	13
	("Coronary Artery Dissection, Spontaneous" [Supplementary Concept]) AND "Pakistan"[Mesh]	0
26	“SCAD” AND “Palestine”	0
	"Spontaneous Coronary Artery Dissection" OR "SCAD" AND “Palestine”	0
	("Coronary Artery Dissection, Spontaneous" [Supplementary Concept]) AND "Palestine"[Mesh]	N/A
27	“SCAD” AND “Qatar”	3
	"Spontaneous Coronary Artery Dissection" OR "SCAD" AND “Qatar”	5
	("Coronary Artery Dissection, Spontaneous" [Supplementary Concept]) AND "Qatar"[Mesh]	0
28	“SCAD” AND “Saudi Arabia”	13
	"Spontaneous Coronary Artery Dissection" OR "SCAD" AND “Saudi Arabia”	17
	("Coronary Artery Dissection, Spontaneous" [Supplementary Concept]) AND "Saudi Arabia"[Mesh]	0
29	“SCAD” AND “Somalia”	1
	"Spontaneous Coronary Artery Dissection" OR "SCAD" AND “Somalia”	2
	("Coronary Artery Dissection, Spontaneous" [Supplementary Concept]) AND "Somalia"[Mesh]	0
30	“SCAD” AND “Sudan”	0

	"Spontaneous Coronary Artery Dissection" OR "SCAD" AND “Sudan”	0
	("Coronary Artery Dissection, Spontaneous" [Supplementary Concept]) AND ("Sudan"[Mesh] OR "South Sudan"[Mesh])	0
31	“SCAD” AND “Syria”	0
	"Spontaneous Coronary Artery Dissection" OR "SCAD" AND “Syria”	1
	("Coronary Artery Dissection, Spontaneous" [Supplementary Concept]) AND "Syria"[Mesh]	1
32	“SCAD” AND “Tunisia”	2
	"Spontaneous Coronary Artery Dissection" OR "SCAD" AND “Tunisia”	3
	("Coronary Artery Dissection, Spontaneous" [Supplementary Concept]) AND "Tunisia"[Mesh]	0
33	“SCAD” AND “United Arab Emirates”	3
	"Spontaneous Coronary Artery Dissection" OR "SCAD" AND “United Arab Emirates”	3
	("Coronary Artery Dissection, Spontaneous" [Supplementary Concept]) AND " United Arab Emirates"[Mesh]	0
34	“SCAD” AND “Yemen”	1
	"Spontaneous Coronary Artery Dissection" OR "SCAD" AND “Yemen”	1
	("Coronary Artery Dissection, Spontaneous" [Supplementary Concept]) AND "Yemen"[Mesh]	0
35	“SCAD” AND “Australia”	69
	"Spontaneous Coronary Artery Dissection" OR "SCAD" AND “Australia”	90
	("Coronary Artery Dissection, Spontaneous" [Supplementary Concept]) AND ("Australia"[Mesh] OR "Western Australia"[Mesh] OR "South Australia"[Mesh])	8
36	“SCAD” AND “Brunei”	0
	"Spontaneous Coronary Artery Dissection" OR "SCAD" AND “Brunei”	0
	("Coronary Artery Dissection, Spontaneous" [Supplementary Concept]) AND "Brunei"[Mesh]	0
37	“SCAD” AND “Cambodia”	1
	"Spontaneous Coronary Artery Dissection" OR "SCAD" AND “Cambodia”	1
	("Coronary Artery Dissection, Spontaneous" [Supplementary Concept]) AND "Cambodia"[Mesh]	0
38	“SCAD” AND “China”	236
	"Spontaneous Coronary Artery Dissection" OR "SCAD" AND “China”	236
	("Coronary Artery Dissection, Spontaneous" [Supplementary Concept]) AND "China"[Mesh]	0
39	“SCAD” AND “Cook Islands”	0
	"Spontaneous Coronary Artery Dissection" OR "SCAD" AND “Cook Islands”	0
	("Coronary Artery Dissection, Spontaneous" [Supplementary Concept]) AND ("Polynesia"[Mesh] OR "Cook Islands Maori people" [Supplementary Concept])	0

40	“SCAD” AND “Fiji”	0
	"Spontaneous Coronary Artery Dissection" OR "SCAD" AND “Fiji”	0
	("Coronary Artery Dissection, Spontaneous" [Supplementary Concept]) AND ("Fiji"[Mesh] OR "Fijian people" [Supplementary Concept])	0
41	“SCAD” AND “Japan”	97
	"Spontaneous Coronary Artery Dissection" OR "SCAD" AND “Japan”	139
	("Coronary Artery Dissection, Spontaneous" [Supplementary Concept]) AND "Japan"[Mesh]	2
42	“SCAD” AND “Kiribati”	0
	"Spontaneous Coronary Artery Dissection" OR "SCAD" AND “Kiribati”	0
	("Coronary Artery Dissection, Spontaneous" [Supplementary Concept]) AND ("Micronesia"[Mesh] OR "Kiribati people" [Supplementary Concept])	0
43	“SCAD” AND “Laos”	0
	"Spontaneous Coronary Artery Dissection" OR "SCAD" AND “Laos”	0
	("Coronary Artery Dissection, Spontaneous" [Supplementary Concept]) AND "Laos"[Mesh]	0
44	“SCAD” AND “Malaysia”	14
	"Spontaneous Coronary Artery Dissection" OR "SCAD" AND “Malaysia”	17
	("Coronary Artery Dissection, Spontaneous" [Supplementary Concept]) AND "Malaysia"[Mesh]	0
45	“SCAD” AND “Marshall Islands”	0
	"Spontaneous Coronary Artery Dissection" OR "SCAD" AND “Marshall Islands”	0
	("Coronary Artery Dissection, Spontaneous" [Supplementary Concept]) AND ("Micronesia"[Mesh] OR "Marshallese people" [Supplementary Concept])	0
46	“SCAD” AND “Micronesia”	0
	"Spontaneous Coronary Artery Dissection" OR "SCAD" AND “Micronesia”	0
	("Coronary Artery Dissection, Spontaneous" [Supplementary Concept]) AND ("Micronesia"[Mesh] OR "Pacific Islands"[Mesh])	4
47	“SCAD” AND “Mongolia”	1
	"Spontaneous Coronary Artery Dissection" OR "SCAD" AND “Mongolia”	1
	("Coronary Artery Dissection, Spontaneous" [Supplementary Concept]) AND "Mongolia"[Mesh]	0
48	“SCAD” AND “Nauru”	0
	"Spontaneous Coronary Artery Dissection" OR "SCAD" AND “Nauru”	0
	("Coronary Artery Dissection, Spontaneous" [Supplementary Concept]) AND ("Nauruan people" [Supplementary Concept] OR "Micronesia"[Mesh])	0

49	"SCAD" AND "New Zealand"	13
	"Spontaneous Coronary Artery Dissection" OR "SCAD" AND "New Zealand"	19
	("Coronary Artery Dissection, Spontaneous" [Supplementary Concept]) AND "New Zealand"[Mesh]	4
50	"SCAD" AND "Niue"	0
	"Spontaneous Coronary Artery Dissection" OR "SCAD" AND "Niue"	0
	("Coronary Artery Dissection, Spontaneous" [Supplementary Concept]) AND "Polynesia"[Mesh]	0
51	"SCAD" AND "Palau"	0
	"Spontaneous Coronary Artery Dissection" OR "SCAD" AND "Palau"	0
	("Coronary Artery Dissection, Spontaneous" [Supplementary Concept]) AND "Palau"[Mesh]	0
52	"SCAD" AND "Papua New Guinea"	1
	"Spontaneous Coronary Artery Dissection" OR "SCAD" AND "Papua New Guinea"	1
	("Coronary Artery Dissection, Spontaneous" [Supplementary Concept]) AND "Papua New Guinea"[Mesh]	0
53	"SCAD" AND "Philippines"	3
	"Spontaneous Coronary Artery Dissection" OR "SCAD" AND "Philippines"	3
	("Coronary Artery Dissection, Spontaneous" [Supplementary Concept]) AND "Philippines"[Mesh]	0
54	"SCAD" AND "Samoa"	0
	"Spontaneous Coronary Artery Dissection" OR "SCAD" AND "Samoa"	0
	("Coronary Artery Dissection, Spontaneous" [Supplementary Concept]) AND ("Samoa"[Mesh] OR "Independent State of Samoa"[Mesh] OR "American Samoa"[Mesh])	0
55	"SCAD" AND "Singapore"	7
	"Spontaneous Coronary Artery Dissection" OR "SCAD" AND "Singapore"	12
	("Coronary Artery Dissection, Spontaneous" [Supplementary Concept]) AND "Singapore"[Mesh]	1
56	"SCAD" AND "Solomon Islands"	0
	"Spontaneous Coronary Artery Dissection" OR "SCAD" AND "Solomon Islands"	0
	("Coronary Artery Dissection, Spontaneous" [Supplementary Concept]) AND ("Melanesia"[Mesh] OR "Solomon Islanders" [Supplementary Concept])	0
57	"SCAD" AND "South Korea"	18
	"Spontaneous Coronary Artery Dissection" OR "SCAD" AND "South Korea"	19
	("Coronary Artery Dissection, Spontaneous" [Supplementary Concept]) AND "Republic of Korea"[Mesh]	0
58	"SCAD" AND "Taiwan"	14
	"Spontaneous Coronary Artery Dissection" OR "SCAD" AND "Taiwan"	17
	("Coronary Artery Dissection, Spontaneous" [Supplementary Concept]) AND "Taiwan"[Mesh]	0

59	“SCAD” AND “Tonga”	0
	"Spontaneous Coronary Artery Dissection" OR "SCAD" AND “Tonga”	0
	("Coronary Artery Dissection, Spontaneous" [Supplementary Concept]) AND ("Tonga"[Mesh] OR "Tonga people" [Supplementary Concept])	0
60	“SCAD” AND “Tuvalu”	0
	"Spontaneous Coronary Artery Dissection" OR "SCAD" AND “Tuvalu”	0
	("Coronary Artery Dissection, Spontaneous" [Supplementary Concept]) AND "Micronesia"[Mesh]	0
61	“SCAD” AND “Vanuatu”	0
	"Spontaneous Coronary Artery Dissection" OR "SCAD" AND “Vanuatu”	0
	("Coronary Artery Dissection, Spontaneous" [Supplementary Concept]) AND ("Vanuatu"[Mesh] OR "Ni-Vanuatu people" [Supplementary Concept])	0
62	“SCAD” AND “Vietnam”	10
	"Spontaneous Coronary Artery Dissection" OR "SCAD" AND “Vietnam”	10
	("Coronary Artery Dissection, Spontaneous" [Supplementary Concept]) AND "Vietnam"[Mesh]	0
	Total	**1602**

Table S3. Excluded studies

No.	Study	Reason for exclusion
1.	Alfonso F, Salamanca J, Díez-Villanueva P. Spontaneous coronary artery dissection in Japan: Different from western countries?. *Int J Cardiol*. 2020;316:49-51. doi:10.1016/j.ijcard.2020.05.097	Editorial
2.	Yalta K, Ucar F, Yilmaztepe M, Ozkalayci F. Tako-tsubo cardiomyopathy and spontaneous coronary artery dissection: A subtle association with prognostic implications?. *Int J Cardiol*. 2016;202:174-176. doi:10.1016/j.ijcard.2015.08.152	Commentary
3.	Xu C, Wang G, He J. Precipitating factors in patients with spontaneous coronary artery dissection: Emotional stressors matter. *Int J Cardiol*. 2023;389:131169. doi:10.1016/j.ijcard.2023.131169	Commentary
4.	Tajrishi FZ, Ahmad A, Jamil A, et al. Spontaneous coronary artery dissection and associated myocardial bridging: Current evidence from cohort study and case reports. *Med Hypotheses*. 2019;128:50-53. doi:10.1016/j.mehy.2019.05.012	Review article
5.	Aoki J, Nakazawa G, Onuma Y, Tanabe K, Nakajima H. Acute myocardial infarction due to spontaneous coronary artery dissection detected three days before by 64-slice spiral computed tomography. *EuroIntervention*. 2006;2(2):272.	Case report
6.	Mishra K, Junday K, Wong CMY, et al. Generation of VCCRIi001-A, a human induced pluripotent stem cell line, from a patient with spontaneous coronary artery dissection. *Stem Cell Res*. 2019;41:101584. doi:10.1016/j.scr.2019.101584	Case report
7.	Nishi T, Kume T, Ueno M, Kobayashi Y, Uemura S. Resolution of spontaneous coronary artery dissection involving the left main coronary artery. *Cardiovasc Interv Ther*. Published online March 23, 2024. doi:10.1007/s12928-024-00996-5	Case report
8.	Bax M, Junday K, Iismaa SE, et al. Generation of induced pluripotent stem cell lines from a sister pair who suffered post-partum or recurrent Spontaneous Coronary Artery Dissections. *Stem Cell Res*. 2023;73:103238. doi:10.1016/j.scr.2023.103238	Case report (2 cases)
9.	Shah N, Michel J, Aitken SA, Harding SA. Outcomes following conservative management of spontaneous coronary artery dissection. *Heart Lung Circ*. 2014;23(10):e193-e196. doi:10.1016/j.hlc.2014.03.028	Case report (3 cases)
10.	Kim SK, Wing-Lun E, Chandrasekhar J, et al. The Australian New Zealand Spontaneous Coronary Artery Dissection (ANZ-SCAD) Registry - A Multi-Centre Cohort Study: Protocol, Background and Significance. *Heart Lung Circ*. 2022;31(12):1612-1618. doi:10.1016/j.hlc.2022.08.018	Study protocol and design
11.	Karimi A, Razaghi R, Koyama M. A patient-specific numerical modeling of the spontaneous coronary artery dissection in relation to atherosclerosis. *Comput Methods Programs Biomed*. 2019;182:105060. doi:10.1016/j.cmpb.2019.105060	Laboratory study from cadavers
12.	Chai T, Tian M, Yang X, Qiu Z, Lin X, Chen L. Genome-Wide Identification of Associations of Circulating Molecules With Spontaneous Coronary Artery Dissection and Aortic Aneurysm and Dissection. *Front Cardiovasc Med*. 2022;9:874912. Published 2022 Apr 27. doi:10.3389/fcvm.2022.874912	Genome-wide identification
13.	Chai T, Tian M, Yang X, Qiu Z, Lin X, Chen L. Genome-Wide Identification of RNA Modifications for Spontaneous Coronary Aortic Dissection. *Front Genet*. 2021;12:696562. Published 2021 Jul 2. doi:10.3389/fgene.2021.696562	Genome-wide identification

14.	Mori R, Macaya F, Sara JD, et al. Non-invasive assessment of endothelial function in patients with spontaneous coronary artery dissection: A case-control study. *Int J Cardiol*. 2020;316:40-42. doi:10.1016/j.ijcard.2020.04.049	Population from Spain (active cases) and the United States (control)
15.	Al-Hussaini A, Abdelaty AMSEK, Gulsin GS, et al. Chronic infarct size after spontaneous coronary artery dissection: implications for pathophysiology and clinical management. *Eur Heart J*. 2020;41(23):2197-2205. doi:10.1093/eurheartj/ehz895	Population from the United Kingdom
16.	Elzanaty AM, Maraey A, Khalil M, et al. Sex Differences in Outcomes of Spontaneous Coronary Artery Dissection: Insights From the National Readmissions Database. *Cardiovasc Revasc Med*. 2022;39:123-124. doi:10.1016/j.carrev.2021.09.002	Population from the United States
17.	Baechler CJ, Witt DR, Lohese O, Benson G. Spontaneous Coronary Artery Dissection and Evidence-Based Medicine. *Am J Cardiol*. 2022;171:65-68. doi:10.1016/j.amjcard.2022.01.046	Population from the United States
18.	Henkin S, Negrotto SM, Tweet MS, et al. Spontaneous coronary artery dissection and its association with heritable connective tissue disorders. *Heart*. 2016;102(11):876-881. doi:10.1136/heartjnl-2015-308645	Population from the United States
19.	Faden MS, Bottega N, Benjamin A, Brown RN. A nationwide evaluation of spontaneous coronary artery dissection in pregnancy and the puerperium. *Heart*. 2016;102(24):1974-1979. doi:10.1136/heartjnl-2016-309403	Population from the United States
20.	Ghani AR, Inayat F, Ali NS, et al. Spontaneous Coronary Artery Dissection: A Case Series of 9 Patients With Literature Review. *J Investig Med High Impact Case Rep*. 2018;6:2324709618770479. Published 2018 Apr 18. doi:10.1177/2324709618770479	Population from the United States
21.	Elkaryoni A, Klappa A, Elgendy IY, et al. Outcomes of ST-Elevation Myocardial Infarction Because of Spontaneous Coronary Artery Dissection Stratified by Involved Coronary Artery. *Am J Cardiol*. 2022;165:135-136. doi:10.1016/j.amjcard.2021.11.004	Population from the United States
Study related to included studies	Objectives	Conclusions
Eastern Mediterranean region		
22.<br>Daoulah et al 2020 *Crit Pathw Cardiol*. 2020 [1]	• To compare in-hospital and follow-up adverse cardiovascular events in patients with and without VA at presentation.	• In-hospital adverse CV events were significantly more frequent for patients with SCAD who presented with VA but was not significant at follow-up.
23.<br>Daoulah et al 2021 *Crit Pathw Cardiol*. 2021 [2]	• To compare demographics, risk factors, associated conditions, clinical presentation, diagnostics, therapeutic, and prognostic implications in patients with SCAD and patients with ACPD from 4 gulf countries.	• Despite a completely different pathophysiology of ACS between SCAD and ACPD, in-hospital and follow-up events were comparable.
24.<br>Daoulah et al 2021 *Curr Probl Cardiol*. 2021 [3]	• To investigate in-hospital and follow-up events in patients with SCAD, stratified by presence or absence of reported stress as a trigger, in 4 Arab Gulf countries.	• In-hospital and follow-up events were comparable in patients with SCAD in the presence or absence of reported stress as a trigger.<br>• Females displayed more emotional stress; whereas, males displayed more physical and combined stress.<br>• Recall, cultural, and gender biases may be contributing factors.

25. Daoulah et al 2021 *Curr Cardiol Rev*. 2021 [4]	• To examine the association between employment status and in-hospital and follow-up adverse cardiovascular events in patients with SCAD.	• Adverse CV events were significantly worse for patients with SCAD who were unemployed.
Western Pacific Region		
26. Adlam et al 2019 [5]	• To investigate the association between rs9349379 and SCAD, to assess whether, at this locus, SCAD is genetically closer to FMD, given their clinical overlap, or to atherosclerotic CAD and acute MI.	• The first genetic risk factor for SCAD was identified in the largest study conducted to date for this condition. This genetic link may contribute to the clinical overlap between SCAD and FMD.
27. Georges et al 2021 [6]	• To identify rare genetic causes to elucidate molecular mechanisms implicated in FMD and SCAD.	• The study shows that rare genetic mutations in PTGIR are enriched among FMD patients and found in SCAD patients, suggesting a role for prostacyclin signalling in non-atherosclerotic stenosis and dissection.
References		
	1. Daoulah A, Al-Faifi SM, Alsheikh-Ali AA, Hersi AS, Lotfi A; G-SCAD investigators. Ventricular Arrhythmias in Patients with Spontaneous Coronary Artery Dissection: Findings from the Gulf Spontaneous Coronary Artery Dissection (Gulf SCAD) Registry. *Crit Pathw Cardiol*. 2020;19(3):146-152.<br>2. Daoulah A, Al-Faifi SM, Madan M, et al. Clinical Presentation and Outcome of Patients With Spontaneous Coronary Artery Dissection Versus Atherosclerotic Coronary Plaque Dissection. *Crit Pathw Cardiol*. 2021;20(1):36-43.<br>3. Daoulah A, Al-Faifi SM, Hersi AS, et al. Spontaneous Coronary Artery Dissection in Relation to Physical and Emotional Stress: A Retrospective Study in 4 Arab Gulf Countries. *Curr Probl Cardiol*. 2021;46(3):100484.<br>4. Daoulah A, Al-Faifi SM, Hurley WT, et al. Spontaneous Coronary Artery Dissection: Does Being Unemployed Matter? Insights from the GSCAD Registry. *Curr Cardiol Rev*. 2021;17(3):328-339.<br>5. Adlam D, Olson TM, Combaret N, et al. Association of the PHACTR1/EDN1 Genetic Locus With Spontaneous Coronary Artery Dissection. *J Am Coll Cardiol*. 2019;73(1):58-66.<br>6. Georges A, Albuisson J, Berrandou T, et al. Rare loss-of-function mutations of PTGIR are enriched in fibromuscular dysplasia. *Cardiovasc Res*. 2021;117(4):1154-1165.	

Abbreviations: ACPD, atherosclerotic coronary plaque dissection; ACS, acute coronary syndrome; CAD, coronary artery disease; CV, cardiovascular; FMD, fibromuscular dysplasia; MI, myocardial infarction; SCAD, spontaneous coronary artery dissection; VA, ventricular arrhythmias.

Table S4. Studies from Australia, New Zealand, and Cambodia

	Study
	The main ethnic group in Australia and New Zealand is the Europeans, i.e., in more than 70.0% of the population.<br>References:<br>• Ancestry | Australia | Community profile (id.com.au) – accessed June 2024<br>• Australia Demographics Profile (indexmundi.com) – accessed June 2024<br>• Ethnic group summaries reveal New Zealand's multicultural make-up | Stats NZ – accessed June 2024
	**Data from studies are presented in Tables S5 – S20**.<br>Caucasian ethnicity in the two countries ranged from 81.0% to 92.0% of patients in three of the retrieved studies that reported ethnicity. [1-3]
1	Dang Q, Murphy B, Graham RM, et al. Patients' perspective of quality-of-care and its correlation to quality-of-life following spontaneous coronary artery dissection. *Eur J Cardiovasc Nurs*. Published online September 14, 2023.
2	McAlister CP, Yi M, Adamson PD, et al. Trends in the Detection, Management and 30-Day Outcomes of Spontaneous Coronary Artery Dissection: A Six-Year, New Zealand Centre Experience. *Heart Lung Circ*. 2021;30(1):78-85.
3	Wong B, To A, El-Jack S. Spontaneous coronary artery dissection: insights from computed tomography coronary angiography follow-up. *N Z Med J*. 2022;135(1555):41-47. Published 2022 May 20.
	Other studies
4	Rashid HN, Wong DT, Wijesekera H, et al. Incidence and characterisation of spontaneous coronary artery dissection as a cause of acute coronary syndrome--A single-centre Australian experience. *Int J Cardiol*. 2016;202:336-338.
5	McGrath-Cadell L, McKenzie P, Emmanuel S, Muller DW, Graham RM, Holloway CJ. Outcomes of patients with spontaneous coronary artery dissection. *Open Heart*. 2016;3(2):e000491. Published 2016 Aug 24.
6	Adams H, Paratz E, Somaratne J, et al. Different patients, different outcomes: A case-control study of spontaneous coronary artery dissection versus acute coronary syndrome. *J Interv Cardiol*. 2018;31(1):41-47.
7	Yuvaraj J, Lin A, Nerlekar N, et al. Is spontaneous coronary artery dissection (SCAD) related to vascular inflammation and epicardial fat? -insights from computed tomography coronary angiography. *Cardiovasc Diagn Ther*. 2020;10(2):239-241.
8	Fahey JK, Chew A, Ihdayhid AR, et al. Women With Spontaneous Coronary Artery Dissection Are at Increased Risk of Iatrogenic Coronary Artery Dissection. *Heart Lung Circ*. 2021;30(1):e23-e28.
9	Murphy BM, Rogerson MC, Hesselson S, Iismaa SE, Graham RM, Jackson AC. Psychosocial impacts of spontaneous coronary artery dissection: A qualitative study. *PLoS One*. 2022;17(9):e0273978. Published 2022 Sep 6. doi:10.1371/journal.pone.0273978
10	Murphy BM, Rogerson MC, Hesselson S, et al. Prevalence of Anxiety, Depression, and Distress in SCAD and Non-SCAD AMI Patients: A Comparative Study. *J Cardiopulm Rehabil Prev*. 2023;43(5):338-345.
11	Murphy BM, Rogerson MC, Le Grande MR, et al. Psychosocial and lifestyle impacts of spontaneous coronary artery dissection: A quantitative study. *PLoS One*. 2024;19(1):e0296224. Published 2024 Jan 5.
12	Tarr I, Hesselson S, Iismaa SE, et al. Exploring the Genetic Architecture of Spontaneous Coronary Artery Dissection Using Whole-Genome Sequencing. *Circ Genom Precis Med*. 2022;15(4):e003527.
13	Tarr I, Hesselson S, Troup M, et al. Polygenic Risk in Families With Spontaneous Coronary Artery Dissection. *JAMA Cardiol*. 2024;9(3):254-261.
	A study from Cambodia: Case series of eight patients was not discussed as six patients (75.0%) were of Caucasian ethnicity.<br>Uribe CE, Ramirez-Barrera JD, Rubio C, et al. Spontaneous coronary artery dissection: Case series from two institutions with literature review. *Anatol J Cardiol*. 2015;15(5):409-415.

Table S5. Study objective, conclusion, diagnosis, and exclusion criteria

WHO region<br>Study	Objectives and Conclusions	Diagnosis	Exclusion criteria
Eastern Mediterranean region			
Benahmed et al 2018	• To report experience of catheterization laboratory to highlight 7 cases of SCAD.<br>• SCAD is a rare entity that is often overlooked and presents a challenge in diagnosis and treatment.	• Diagnosis by CAG; only Type 1 was identified by visualization of a radiolucent intimal flap. [1,3]	• Iatrogenic coronary dissection or aortic dissection.
Daoulah et al 2021	• To evaluate epidemiology and outcomes of patients presenting with SCAD in the Arab Gulf countries.<br>• G-SCAD registry showed similarities and differences as compared with previous studies. Percentages of male and female with SCAD were balanced. Females with SCAD had numerically worse outcomes. Males had more common conventional CAD risk factors.	• SCAD according to CAG classification by Saw et al. [1,2]	• Atherosclerotic plaque rupture or plaque erosion, and occlusive thrombus.<br>• Iatrogenic Coronary artery dissection.
Almasi et al 2022	• To determine prognoses and characteristics of adult women with SCAD who presented with STEMI.<br>• STEMI due to SCAD is one of the known causes of STEMI in young women. SCAD patients had more favourable prognoses in both conservative and revascularization management modalities than patients with STEMI due to atherosclerotic lesion.	• Diagnosis by CAG with Saw SCAD classification.	• No CAG for any reason.<br>• 3 negative troponin tests.
South-East Asia region			
Sharma et al 2016	• To describe a case series from South Asia and highlight long-term outcomes on conservative management.<br>• Clinical recognition of SCAD is difficult. It should be suspected in peripartum state, young females and in presence of other precipitating factors. CAG is essential for establishing diagnosis. Medical treatment provides favourable long-term survival.	• Diagnosis by CAG: (1) Visualization of thin radiolucent intimal flap and presence of extraluminal contrast after wash out of dye from the remainder of the vessel; (2) Presence of haziness and irregularity of the vessel. Haziness and intraluminal filling defects indicate possible thrombus.<br>• Classification by Saw et al. [1]	• Patients with coronary dissection who had any history of cardiac surgery, coronary intervention, trauma, or aortic dissection.
Valappil et al 2018	• To study clinical, angiographic, and technical characteristics of patients with SCAD undergoing PCI.<br>• PCI is associated with fair rate of technical success.	• Diagnosis by presence of CAG-confirmed dissection plane in a major epicardial coronary artery with or without coexisting atherosclerosis. [4]	• Iatrogenic dissection
Western Pacific Region			
Rashid et al 2016	• To describe incidence and characterisation of SCAD as cause of ACS.<br>• SCAD as a cause of ACS is becoming more frequent. Although it is more relevant to females < 60 years, older patients may also be	• Angiographic diagnoses according to Tweet et al and Saw et al. [5,6]	-

WHO region<br>Study	Objectives and Conclusions	Diagnosis	Exclusion criteria
	affected. Uncertainties over best practice are becoming increasingly resolved.		
McGrath-Cadell et al 2016	• To investigate characteristics and outcomes of patients with SCAD.<br>• Patients with SCAD often had multiple coronary territories and extracardiac vascular abnormalities, suggesting a systemic vascular process, which may explain the high incidence of migraine. Patients should be screened for FMD and followed up due to possible recurrence.	• Diagnosis by treating cardiologists or self-identified SCAD patients if were enrolled as result of a social media survey.<br>• Diagnosis by CAG as Type 1, 2 or 3. [7]	-
Adams et al 2018	• To identify SCAD cases and compare risk factors, presentation, and management outcomes with ACS controls.<br>• SCAD affects young females without CV risk factors. Major risk factors for SCAD were depression, anxiety, or neuropsychiatric illness. A conservative approach led to similar MACCE compared with ACS controls.	-	-
Yuvaraj et al 2020	• To investigate EAT, density and PCAT attenuation as CTCA markers of vascular inflammation in the context of SCAD.<br>• Vascular inflammation may harbour a limited role in SCAD pathophysiology.	-	-
Fahey et al 2021	• To compare rate of iatrogenic coronary artery dissection in women with and without SCAD.<br>• Iatrogenic dissection rate in women with SCAD during cardiac catheterisation is potentially high and significantly higher than women without SCAD. This may indicate generalised coronary fragility.	• Diagnosis by CAG: abrupt vessel tapering, evidence of dissection flap by presence of radiolucent plane, or contrast staining within arterial wall, or mimicking atherosclerosis. [1]	• Iatrogenic coronary dissection
Murphy et al 2022	• To investigate psychosocial impacts of SCAD in Australian SCAD survivors.<br>• There is lack of information as an over-arching issue which exacerbates all other psychosocial impacts, magnifying SCAD survivors' emotional distress and self-management challenges.	• Self-reported	-
Murphy et al 2023	• To compare rates of anxiety, depression, and distress in acute MI patients with and without SCAD.<br>• Anxiety, depression, and distress are more common after SCAD-AMI than after traditional acute MI.	• Self-reported	-
Murphy et al 2024	• To document prevalence and predictors of a range of psychosocial and lifestyle impacts of SCAD.<br>• Findings extend on previous investigation by reporting prevalence of each of 48 psychosocial and 5 lifestyle impacts identified in an earlier focus group.	-	-

WHO region<br>Study	Objectives and Conclusions	Diagnosis	Exclusion criteria
Dang et al 2023	• To investigate SCAD survivors' perceptions of their quality-of-care and its relationship to quality-of-life.<br>• While SCAD survivors rated their overall hospital care highly, healthcare providers' knowledge of SCAD was perceived to be poor, and the most common source of SCAD information was the internet. Mental health conditions were common, and a significant association was observed between perceived quality-of-care and SCAD survivors' quality-of-life.	• Self-reported	-
Tarr et al 2022	• To clarify genetic cause of SCAD using targeted and genome-wide methods in sporadic cases. Also, to identify potentially damaging coding and noncoding rare genetic variants in SCAD- and CTD-associated genes by using WGS of unrelated SCAD patients.<br>• SCAD shares some genetic overlap with CTD, even in the absence of major CTD phenotype.	-	-
Tarr et al 2024	• To determine contribution of rare and common genetic variants to SCAD risk in familial cases, the latter via the comparison of a polygenic risk score with those with sporadic SCAD and healthy controls.<br>• Extreme aggregation of common genetic risk appears to play a significant role in familial clustering of SCAD as well as in sporadic case predisposition.	-	-
McAlister et al 2021	• To assess trends in detection, management, and outcomes of patients with SCAD.<br>• Detection of SCAD has increased and is an important cause of ACS in younger women. This has been driven by increased number of NSTEMI patients diagnosed with SCAD, associated with an improved 30-day MACE.	• Diagnosis of SCAD was made as per the definition described by Saw et al. [1]	• Iatrogenic dissection or significant (> 50% stenosis) coronary atherosclerosis.
Wong et al 2022	• To investigate the diagnostic performance of CTCA in patients with SCAD compared to invasive CAG. To understand natural history of SCAD via follow-up CTCA.<br>• Utilizing CTCA in diagnosing SCAD remains challenging due to limitations in spatial and temporal resolution, particularly in distal vessels. Optimal timing of CTCA to assess dissection healing was 80 days.	• Diagnosis by CAG.<br>• Classified according to Saw et al. [1,8]	-
Uribe et al 2015	• To conduct a demographic descriptive analysis of patients with SCAD and determine ACS type the patient presented and document treatment received.	• Diagnosis by CAG according to NHLBI classification. [9]	-

WHO region<br>Study	Objectives and Conclusions	Diagnosis	Exclusion criteria
Meng et al 2017	• To focus on the characteristics of SCAD as a cause of young female acute MI population in Jiangsu, China.<br>• SCAD has high prevalence in young female acute MI population.	• SCAD defined as spontaneous separation of coronary vessel wall together with typical diagnostic features (intimal dissection or intramural hematoma) identified by CAG and IVUS or OCT. [1]<br>• Intimal dissection was defined as the presence of multiple radiolucent lumens, with or without contrast staining. Intramural hematoma was identified by an abrupt vessel tapering. [1]	• PCI-related dissection, iatrogenic, trauma, and atherosclerotic changes.
Sun et al 2019	• To identify genes associated with SCAD development in Chinese Han population.<br>• TSR1 is a potential causal gene, which might lead to further progress in diagnosis and treatment of SCAD.	• Diagnosis by CAG, IVUS, or OCT (intimal dissection and intramural hematoma).	• Iatrogenic dissections
Liu et al 2019	• To describe clinical and angiographic features in SCAD and evaluate treatment and long-term prognosis of SCAD in China.<br>• SCAD was associated with atherosclerosis and predominantly affected male population. SCAD often affected RCA and caused a short dissection. In-hospital mortality rate was low regardless of therapeutic strategy. Significantly better long-term prognosis was observed in revascularization compared with conservative therapy.	• SCAD defined as the presence of a longitudinal radiolucent linear image in at least two orthogonal projections on CAG.<br>• NHLBI classification was used to characterize SCAD lesions. [9]	• Iatrogenic and traumatic SCD
Hui et al 2020	• To explore value of plasma fibrillin-1 levels in patients with SCAD.<br>• Plasma fibrillin-1 is a promising biomarker for aiding diagnosis of SCAD and ma y have value in prognosis prediction.	• Diagnosis by CAG or OCT (intimal dissection and intramural hematoma) in the absence of atherosclerosis. Classified according to Saw et al. [1,2]	• Traumatic or iatrogenic dissections
Chang et al 2022	• To study characteristics, management, and prognosis of SCIH.<br>• SCIH occurs more often in post-menopausal women, and many of them had hypertension. LAD, most affected artery, displayed diffuse narrowing, and end segment might be involved leading to insufficient perfusion. Conservative treatment may be the best choice. SCIH patients had good prognosis with reduced dosage and shorter course of antiplatelet treatment. SCIH patients may not be administered antiplatelet drugs, particularly dual antiplatelet drugs.	• SCIH was defined as SCAD without obvious intima tear on CAG, i.e., non-classical SCAD (Type 1). [10,11]<br>• SCIH classified as Type 1, 2, and 3 in this study based on CAG findings.	-
Ma et al 2023	• To compare clinical and angiographic characteristics of high- and low-risk SCAD patients to determine optimal treatment strategy.<br>• Conservative treatment is recommended strategy for low-risk patients. PCI can be considered in high-risk patients with favourable outcomes and vessel healing.	• Lesion classified based on Saw classification.<br>• High- or low-risk SCAD according to lesion segment location (segment with dissection or intramural hematoma). High-risk: involving LM artery or proximal segment of any main coronary	• Patients with atherosclerotic, traumatic or iatrogenic dissection

WHO region<br>Study	Objectives and Conclusions	Diagnosis	Exclusion criteria
		artery. Low-risk: involving side branch or middle and distal segments of any main artery. [10]	
Tokura et al 2014	• To review patients with CAG-confirmed SCAD to provide insight into diagnosis and treatment of SCAD.<br>• SCAD appears to be rare, but it should be considered in ACS patients, especially in younger female.	• Diagnosis by CAG in the absence of coronary atherosclerosis.	• Iatrogenic coronary artery trauma and atherosclerotic plaque dissection.
Nakashima et al 2016	• To compare prognosis of patients with SCAD and atherosclerosis as cause of acute MI, especially in young females.<br>• Young females with SCAD represent a high-risk group of patients with acute MI and require close follow-up.	• SCAD definition according to Saw et al. [7]	• PCI-related dissection, posttraumatic coronary artery dissection, and atherosclerotic-related dissection.
Nishiguchi et al 2016	• To investigate prevalence of SCAD in ACS using OCT.<br>• SCAD is not a rare cause for ACS, especially in females without classical coronary risk factors.	• Diagnosis by CAG according to NHLBI classification. [9]<br>• Lesions were classified morphologically according to Ambrose classification.<br>• Plaque rupture, thin-cap fibroatheroma, thrombus and calcium deposit in OCT were diagnosed according to the consensus documents. Thrombi divided into high and low backscattering.	• Patients with bypass graft failure or failed to obtain TIMI flow grade 3 by initial thrombus aspiration before OCT.
Nishiguchi et al 2017	• To investigate outcomes of SCAD treated by OCT-guided PCI.<br>• Outcomes were favourable, as well as those for other ACS etiologies.	• SCAD treated by OCT-guided PCI	• Post resuscitation or shock, failure to advance OCT catheter over lesion, stent restenosis, thromboembolism, and urgent CABG.
Kubota et al 2020	• To describe 7 cases in which SCAD in small vessels was detected only on repeat CAG in chronic stage.<br>• In cases where SCAD is suspected but cannot be established at initial CAG or by other diagnostic techniques, repeat CAG may reveal vessel healing, which is suggestive of SCAD occurrence.	-	-
Inohara et al 2020	• To investigate characteristics, treatment pattern, and in-hospital outcome of SCAD compared with non-SCAD using nationwide administrative data in Japan.<br>• In-hospital mortality of SCAD patients was lower and comparable with other international studies. More than half of SCAD patients were managed with PCI.	• Diagnosis of SCAD according to database abstraction<br>• No angiograms were manually reviewed.	• Iatrogenic or coronary artery dissection as procedural complication.

WHO region<br>Study	Objectives and Conclusions	Diagnosis	Exclusion criteria
Inoue et al 2021	• To examine young females who underwent PCI and to determine characteristics, treatment, and long-term clinical outcomes among patients with SCAD.<br>• SCAD was not uncommon among young Japanese women requiring PCI. Patients with SCAD exhibited fewer coronary risk factors and more precipitating factors than those without SCAD. Long-term outcomes after an early period were favourable.	• Diagnosis by CAG. IVUS or OCT were used as needed.<br>• SCAD classified according to Saw et al. [7]	-
Taguchi et al 2023	• To identify clinical characteristics of SCAD patients.<br>• There is a high prevalence of FMD in brachial artery.	• Diagnosis by CAG and classified according to Saw et al. [1,12,13]	-
Kim et al 2021	• To evaluate prevalence, characteristics, and outcomes of SCAD in young females with acute MI in Korea.<br>• SCAD is not rare, among female patients < 60 years with acute MI. Type 2 was most observed on CAG. Distal portion of LAD was most affected. Long-term outcomes were favourable.	• Diagnosis by CAG and classified according to Saw et al. [14]	-
Turkey			
Celik et al 2001	• To describe characteristics of primary SCAD patients.<br>• Primary SCAD is not only disease of middle-aged women without risk factors but can also be seen in middle and older aged males having CAD risk factors. Prognosis of primary SCAD may be more favourable in atherosclerotic patients than non-atherosclerotic ones due to improved collateral circulation in chronic coronary artery atherosclerosis.	• CAG	-
Unal et al 2008	• To show importance of prompt diagnosis and treatment of SCAD.<br>• Early diagnosis of SCAD by CAG is important, as urgent CABG can be lifesaving.	• CAG	-
Canga et al 2018	• To investigate systemic inflammatory activation in patients with an ACS secondary to NA-SCAD.<br>• Degree of inflammatory activation in NA-SCAD-ACS patients was similar to or even greater than that in CAD-ACS patients. This suggested a role of inflammation in pathophysiology of NA-SCAD-ACS.	• NA-SCAD defined as intraluminal filling defect during contrast injection or persistent staining of the artery following contrast injection that was not interpreted as an obstructive coronary atheroma or intracoronary thrombus, and the artery in which the dissection was observed should be free from any coronary atherosclerotic plaque.	• Present or suspected iatrogenic dissection<br>• Non-emergent conditions (i.e., evaluation for stable angina)
Özkan et al 2023	• To investigate whether there is a relationship between triglyceride glucose index and SCAD in young females.<br>• Development of non-atherosclerotic conditions can be predicted by simple biochemical tests in young women with low atherosclerotic CV risk factors.	• CAG<br>• Classified into the 3 types. [15]	• Iatrogenic coronary artery dissection.

WHO region<br>Study	Objectives and Conclusions	Diagnosis	Exclusion criteria
References	Table S6		

Abbreviations: ACS, acute coronary syndrome; AMI, acute myocardial infarction; CABG, coronary artery bypass grafting; CAD, coronary artery disease; CAG, coronary angiography; CR, cardiac rehabilitation; CTCA, computed tomography coronary angiography; CTD, connective tissue disorders; CV, cardiovascular; EAT, epicardial adipose tissue; FMD, fibromuscular dysplasia; G-SCAD, spontaneous coronary artery dissection in the Gulf; IVUS, intracoronary ultrasound; LAD, left anterior descending artery; LM, left main; MACCE, major adverse cardiovascular or cerebrovascular event; MACE, major adverse cardiovascular event; MI, myocardial infarction; NA-SCAD; nonatherosclerotic spontaneous coronary artery dissection; NHLBI, National, Heart, Lung, and Blood Institute; NSTEMI, non-ST segment myocardial infarction; OCT, coherence tomography; PCAT, peri-coronary adipose tissue; PCI, percutaneous coronary intervention; RCA, right coronary artery; SCAD, spontaneous coronary artery dissection; SCIH, Spontaneous coronary intramural hematoma; TIMI, thrombolysis in myocardial infarction; WGS, whole genome sequencing.

Table S6. SCAD classification by included studies

Study	SCAD classification
Benahmed et al 2018<br>Daoulah et al 2021<br>Almasi et al 2022<br>Sharma et al 2016<br>McGrath-Cadell et al 2016<br>McAlister et al 2021<br>Fahey et al 2021<br>Meng et al 2017<br>Hui et al 2020<br>Ma et al 2023<br>Nakashima et al 2016<br>Inoue et al 2021<br>Taguchi et al 2023<br>Kim et al 2021<br>Wong et al 2022<br>Özkan et al 2023	SCAD types or Saw classification: [1-3,7,10,12-15]<br>• Type 1: the classic contrast dye staining of arterial wall with multiple radiolucent lumens with or without the presence of dye hang-up or slow contrast clearing from the lumen. This which can be caused by a tear in the inner lining of the artery or the rupture of vasa vasorum. This leads to the formation of a false lumen, which compresses the true lumen and decreases blood flow to heart.<br>• Type 2: SCAD appears as diffuse (typically 20 to 30 mm) and smooth narrowing that can vary in severity. It is caused by an intramural hematoma within the arterial wall. It often occurs in the mid to distal segments of coronary arteries.<br>• Type 3: SCAD resembles atherosclerosis with focal or tubular stenosis. It can be challenging to distinguish from atherosclerosis without additional intracoronary imaging. It has long lesion (11–20 mm), hazy or linear stenosis but there is lack of atherosclerotic changes in other coronary arteries.<br>• Type II and III need IVUS or OCT test to intramural hematoma or double lumen.
Ma et al 2023	High- or low-risk SCAD according to lesion segment location (segment with dissection or intramural hematoma): [10]<br>• High-risk: involving LM artery or proximal segment of any main coronary artery.<br>• Low-risk: involving side branch or middle and distal segments of any main artery.
Rashid et al 2016	• Angiographic diagnoses according to Tweet et al and Saw et al. [5,6]
Liu et al 2019<br>Nishiguchi et al 2016<br>Uribe et al 2015	Diagnosis by CAG according to NHLBI classification. [9]<br>• Type A: radiolucent within the lumen/no persistence of contrast.<br>• Type B: parallel double lumen separated by a radiolucent area with minimal/no persistence of contrast.<br>• Type C: persistent presence of contrast outside the lumen.<br>• Type D: spiral luminal filling defect.<br>• Type E: dissection with persistent filling defect.<br>• Type F: dissection with total coronary occlusion.
Chang et al 2022	SCIH was defined as SCAD without obvious intima tear on CAG, i.e., non-classical SCAD (Type 1). SCIH classified as Type 1, 2, and 3 in this study based on CAG findings: [10,11]<br>• Type 1 refers to diffuse stenosis (length >20 mm) and can be subclassified as type 1A based on long diffuse arterial narrowing in the middle coronary artery and or type 1B based on diffuse narrowing that extends to the distal tip of the artery, including occlusion of the distal coronary artery.<br>• Type 2 is characterized by focal nonatherosclerotic smooth stenosis, which is usually diagnosed by intracoronary imaging.<br>• Type 3 SCIH includes the involvement of multiple vessels (different coronary arteries rather than one main branch with side branches).<br>The SCIH was classified as type 1, 2, and 3 in this study based on the angiography findings. Type 1 refers to diffuse stenosis (length >20 mm) and can be subclassified as type 1A based on long diffuse arterial narrowing in the middle coronary artery and or type 1B based on

Study	SCAD classification
	diffuse narrowing that extends to the distal tip of the artery, including occlusion of the distal coronary artery. Type 2 is characterized by focal nonatherosclerotic smooth stenosis, which is usually diagnosed by intracoronary imaging. The involvement of multiple vessels (different coronary arteries rather than one main branch with side branches) was classified as type 3 SCIH.
References	
	1. Saw J. Coronary angiogram classification of spontaneous coronary artery dissection. *Catheter Cardiovasc Interv*. 2014;84(7):1115-1122.<br>2. Saw J, Mancini GB, Humphries K, et al. Angiographic appearance of spontaneous coronary artery dissection with intramural hematoma proven on intracoronary imaging. *Catheter Cardiovasc Interv*. 2016;87(2):E54-E61.<br>3. Iskandrian AS, Bemis CE, Kimbiris D, Mintz GS, Hakki AH. Primary coronary artery dissection. *Chest*. 1985;87(2):227-228.<br>4. Saw J. Spontaneous coronary artery dissection. *Can J Cardiol*. 2013;29(9):1027-1033.<br>5. Tweet MS, Eleid MF, Best PJ, et al. Spontaneous coronary artery dissection: revascularization versus conservative therapy. *Circ Cardiovasc Interv*. 2014;7(6):777-786.<br>6. Saw J, Ricci D, Starovoytov A, Fox R, Buller CE. Spontaneous coronary artery dissection: prevalence of predisposing conditions including fibromuscular dysplasia in a tertiary center cohort. *JACC Cardiovasc Interv*. 2013;6(1):44-52.<br>7. Saw J, Aymong E, Sedlak T, et al. Spontaneous coronary artery dissection: association with predisposing arteriopathies and precipitating stressors and cardiovascular outcomes. *Circ Cardiovasc Interv*. 2014;7(5):645-655.<br>8. Tweet MS, Gulati R, Williamson EE, Vrtiska TJ, Hayes SN. Multimodality Imaging for Spontaneous Coronary Artery Dissection in Women. *JACC Cardiovasc Imaging*. 2016;9(4):436-450.<br>9. Huber MS, Mooney JF, Madison J, Mooney MR. Use of a morphologic classification to predict clinical outcome after dissection from coronary angioplasty. *Am J Cardiol*. 1991;68(5):467-471.<br>10. Hayes SN, Kim ESH, Saw J, et al. Spontaneous Coronary Artery Dissection: Current State of the Science: A Scientific Statement From the American Heart Association. *Circulation*. 2018;137(19):e523-e557.<br>11. Adlam D, Alfonso F, Maas A, Vrints C; Writing Committee. European Society of Cardiology, acute cardiovascular care association, SCAD study group: a position paper on spontaneous coronary artery dissection. *Eur Heart J*. 2018;39(36):3353-3368.<br>12. Saw J, Humphries K, Aymong E, et al. Spontaneous Coronary Artery Dissection: Clinical Outcomes and Risk of Recurrence. *J Am Coll Cardiol*. 2017;70(9):1148-1158.<br>13. Saw J, Starovoytov A, Humphries K, et al. Canadian spontaneous coronary artery dissection cohort study: in-hospital and 30-day outcomes. *Eur Heart J*. 2019;40(15):1188-1197.<br>14. Saw J, Mancini GBJ, Humphries KH. Contemporary Review on Spontaneous Coronary Artery Dissection. *J Am Coll Cardiol*. 2016;68(3):297-312.<br>15. Hayes SN, Tweet MS, Adlam D, et al. Spontaneous Coronary Artery Dissection: JACC State-of-the-Art Review. *J Am Coll Cardiol*. 2020;76(8):961-984.

Table S7. General study characteristics

WHO region<br>Study	Study design	Enrolment period	Recruitment duration (year)	Country	Number of sites	Sample size (SCAD patients)	Incidence/ Prevalence of SCAD	Longest follow-up period	Other
Eastern Mediterranean region									
Benahmed et al 2018	Case series	September 2014 to October 2017	3 years	Morocco	1	7	CAG report:<br>Cases: 2000<br>SCAD: 7 (0.35%)	Average 16.4 (1.0 – 29.0) months	-
Daoulah et al 2021	Retrospective descriptive	January 2011 to December 2017	7	Bahrain, Kuwait, KSA, UAE	30	83	CAG reports: 198,000<br>SCAD: 83 (0.04%)	Median 18.8 (9.0-40.0) months	G-SCAD Registry
Almasi et al 2022	Retrospective cohort	March 2017 to August 2020	3.5	Iran	1	15	CAG reports: Young females with STEMI: 311<br>SCAD: 15 (4.8%)	1 year	Young females <60 years with STEMI: STEMI-SCAD vs. STEMI-AS vs. STEMI-others
South-East Asia region									
Sharma et al 2016	Case series	January 1994 to June 2015	11.5	India	1	5 (6 episodes total; 2 for 1 case 17 years apart)	-	7 (2.0 – 19.0)	-
Valappil et al 2018	Retrospective descriptive	January 2013 to January 2017	4	India	1	ACS: 42<br>ACS with PCI: 16	-	8 months to 3 years	Data for patients who underwent PCI (n=16)
Western Pacific Region									
Rashid et al 2016	Retrospective descriptive<br><br>Cohort for females <60 years	August 2013 to July 2014	1	Australia	1	All patients: 21 (23 case; 26 vessels)<br>Females <60 years: 16	ACS cases underwent CAG: 1332<br>SCAD 23 (1.7%)	In-hospital	Females <60 years with ACS: SCAD vs. Athero-occlusive
McGrath-Cadell et al 2016	Retrospective descriptive	-	3	Australia	31	40	-	Median 16 (8.0 - 28.5) months	-
Adams et al 2018	Case-control (matching 1:3)	January 2000 to December 2015	16	Australia	1	22	-	1 year	SCAD vs. PR

WHO region Study	Study design	Enrolment period	Recruitment duration (year)	Country	Number of sites	Sample size (SCAD patients)	Incidence/ Prevalence of SCAD	Longest follow-up period	Other
Yuvaraj et al 2020	Retrospective cohort (letter)	-	-	Australia	1	11	-	CTCA study within 2 weeks of presentation	SCAD vs. Non-ACS asymptomatic control
Fahey et al 2021	Retrospective descriptive	January 2007 to December 2017	11	Australia	1	85	CAG reports: Females with ACS: 485 SCAD: 85 (17.5%)	In-hospital	Females with ACS: SCAD vs. Non-SCAD#
Murphy et al 2022	Qualitative study	-	-	Australia	1	30	-	-	Focused group 0f SCAD survivors
Murphy et al 2023	Survey (online & hard copy)	-	6 months	Australia*	2	35	Patients with acute MI within 6 months: 162* SCAD: 35 (22.2%)	-	Acute MI survivors: SCAD vs. Non-SCAD
Murphy et al 2024	Online survey	February 2023 to May 2023	3months	Australia	1	310	-	-	From same databases of Murphy et al 2022 & Tarr et al 2022
Dang et al 2023 (n=172)	Online survey	September to October 2022	2 months	Australia	Recruitment via social media and other sources	172	-	-	SCAD survivors
Tarr et al 2022	Whole genome sequencing	-	-	Australia	Recruitment via social media	91	-	No outcomes reported	SCAD cases with (n=17) or without (n=74) CTD gene variants
Tarr et al 2024	Whole genome sequencing	-	-	Australia	Recruitment via social media	200	-	No outcomes reported	Sporadic (n=173) vs. Familial (n=27) SCAD
McAlister et al 2021	Retrospective descriptive	January 2014 to January 2020	6	New Zealand	1	113	ANZACS-QI database:	30 days	Used data from ANZACS-QI

WHO region Study	Study design	Enrolment period	Recruitment duration (year)	Country	Number of sites	Sample size (SCAD patients)	Incidence/ Prevalence of SCAD	Longest follow-up period	Other
							SCAD rate 1.6%		database for comparison**
Wong et al 2022	Retrospective descriptive<br><br>Cohort for SCAD lesion	2010 to 2018	8 – 9	New Zealand	1	32 (38 lesions)	-	Time to CTCA F/U cut-off: median 40.5 (20.8 – 107) days	F/U CTCA study: Healed vs. non-healed SCAD lesion
Uribe et al 2015	Case series	2003 to 2012	9	Cambodia	2	8	-	1 year (for 4 cases only)	-
Meng et al 2017	Retrospective descriptive	-	4	China	1	21	Prevalence 21/60 (35%)	No outcomes reported	Females with acute MI
Sun et al 2019	Case control genetic study	November 2011 to January 2018	6	China	Cases: 1<br>Control: 4	85 (92 lesions)	-	No outcomes reported	-
Liu et al 2019	Retrospective descriptive<br><br>Cohort for management	January 2003 to December 2015	13	China	1	118	Prevalence Primary CAG reports: 76,359<br>118 (0.15%)	Median 43 (25.0 – 75.0) months<br><br>12 years (Kaplan-Meier curve)	For all SCAD patients:<br>PCI vs. CABG vs. Medical treatment
Hui et al 2020	Part 1: retrospective case-control<br>Part 2: prospective cohort	January 2014 to September 2018	~5	China	1	70	-	Median 28 months	Part 1: SCAD vs. no-SCAD<br>Part 2: in SCAD patients based on FBN1 level
Chang et al 2022	Retrospective descriptive	January 2012 to December 2018	7	China	1	SCIH: 30 (35 lesions)	ACS patients: 7243<br>SCAD: 141 (1.9%)<br>SCIH: 30 (0.4%)	Mean 29.3±13.5 months	SCIH patients
Ma et al 2023	Retrospective descriptive<br><br>Cohort for SCAD risk	2012 to 2020	8 – 9	China	1	81	Dissection cases (not MI): 531<br>SCAD: 81 (15.2%)	1 year	For all SCAD patients:<br>Low vs. high SCAD risk

WHO region Study	Study design	Enrolment period	Recruitment duration (year)	Country	Number of sites	Sample size (SCAD patients)	Incidence/ Prevalence of SCAD	Longest follow-up period	Other
Tokura et al 2014	Case series	March 2001 to November 2012	12.5	Japan	1	10	All ACS patients: 1159<br>SCAD: 10 (0.86%)	Mean 7.3 months	-
Nakashima et al 2016	Retrospective descriptive<br><br>Retrospective cohort for females	January 2000 to December 2013	14	Japan	20	All: 63<br><br>Young females (≤50 years) for cohorts:<br>130	Overall prevalence<br>All acute MI patients: 20,195<br>SCAD: 63 (0.31%)<br>In young females: 130 (0.64%)	Median 34 (3.0 – 160.0) months<br><br>5 years (Kaplan– Meier curve)	Young females (≤50 years) with acute MI:<br>SCAD vs Non-SCAD
Nishiguchi et al 2016	Retrospective descriptive	November 2008 to February 2012	~3.5	Japan	3	13	ACS with OCT study: 326<br>SCAD: 13 (4.0%)	OCT findings	SCAD vs. PR vs. Non-SCAD/Non-PR#
Nishiguchi et al 2017	Retrospective cohort according to culprit lesion etiology	January 2011 to December 2014	4	Japan	1	12	ACS patients underwent OCT-guided PCI: 306<br><br>SCAD: 12 (3.9%)	17.1±13.3 months	SCAD vs. PR vs. CN vs. UE
Kubota et al 2020	Case series	2010 to 2019	9 – 10	Japan	2	7	-	-	SCAD detected only on repeat CAG in chronic stage
Inohara et al 2020	Retrospective cohort	2012 to 2017	5 – 6	Japan	Nationwide administrative database	322	CAG reports:<br>Females with MI: 68,986<br>SCAD: 322 (0.47%)	In-hospital	Females with MI:<br>SCAD vs. Non-SCAD
Inoue et al 2021	Retrospective descriptive	January 2011 to December 2016	6	Japan	Multicentre registry	19	Prevalence<br>Young females underwent PCI: 187	Median 960 (686 – 1504) days	N-registry<br>Females <60 years &<br>underwent PCI:

WHO region Study	Study design	Enrolment period	Recruitment duration (year)	Country	Number of sites	Sample size (SCAD patients)	Incidence/ Prevalence of SCAD	Longest follow-up period	Other
							SCAD: 19 (10.2%)		SCAD vs. Non-SCAD#
Taguchi et al 2023	Case series	January 2011 to January 2023	12	Japan	1	16	-	In-hospital	-
Kim et al 2021	Retrospective descriptive	November 2005 to September 2017	12	South Korea	1	13	Prevalence CAG reports: Young females with MI: 148 SCAD: 13 (8.8%)	Median 935 (121 - 4125) days	Females <60 years with acute MI: SCAD vs. Non-SCAD#
Turkey									
Celik et al 2001	Case series	-	2 years	Turkey	1	9	CAG reports: 3,750 Primary SCAD: 9 (0.24%)	1 year	-
Unal et al 2008	Case series	January 2001 and August 2006	5.5	Turkey	1	6	Incidence CAG reports: 5,000 SCAD: 6 (0.12%)	1 year	-
Canga et al 2018	Retrospective cohort	2011 to 2015	4 – 5	Turkey	1	22	CAG reports: 30,255 NA-SCAD: 22 (0.07%)	In-hospital	CAG and dissection: NA-SCAD-ACS vs. CAD-ACS vs. ACS no CAD
Özkan et al 2023	Retrospective descriptive	January 2017 to December 2022	6	Turkey	1	49	CAG reports: Females: 3,889 Young females: 281*** SCAD: 49 (17.4%)	No outcomes reported	Young females (≤50 years) with ACS: SCAD vs Non-SCAD#

* With assistance from Stanford University (United States); and as part of a larger international study of cardiac-related emotions that was open to people who had experienced a broad range of cardiac events. Population: 23 (14.0%) from North America.

**An estimate of total ACS was taken from data submitted by study institution to the All New Zealand Acute Coronary Syndrome Quality Improvement (ANZACS-QI) database, a nationwide database recording all patients undergoing angiography for suspected ACS.

***Excluded 12 pregnant women (not accepted for CAG) and 6 patients with chronic kidney or liver disease.

#Compared characteristics only, not outcomes, between the cohorts.

Abbreviations: ACPD, atherosclerotic coronary plaque dissection; ACS, acute coronary syndrome; ANZACS-QI, Zealand Acute Coronary Syndrome Quality Improvement database; CAD, coronary artery disease; CAG, coronary angiography; CN, calcified nodule; CTCA, computed tomography coronary angiography; CTD, connective tissue disorder; F/U, follow-up; FBN1, fibrillin-1; G-SCAD, spontaneous coronary artery dissection in the Gulf; KSA, Kingdom of Saudi Arabia; MI, myocardial infarction; NA-SCAD, nonatherosclerotic spontaneous coronary artery dissection; OCT, Optical coherence tomography; PCI, percutaneous coronary intervention;

WHO region Study	Study design	Enrolment period	Recruitment duration (year)	Country	Number of sites	Sample size (SCAD patients)	Incidence/ Prevalence of SCAD	Longest follow-up period	Other

PR, plaque rupture; SCAD, spontaneous coronary artery dissection; SCIH, Spontaneous coronary intramural hematoma; STEMI, ST segment elevation myocardial infarction; STEMI-SCAD, SCAD due to STEMI; STEMI-AS (STEMI due to an atherosclerotic lesion), and STEMI-others (STEMI due to other etiologies including Takotsubo cardiomyopathy and myopericarditis, as well as STEMI despite a normal epicardial CAG); UAE, United Arab Emirates; UE, undetermined etiology.

Table S8. Demographics and risk factors

WHO region Study	Age (year) Mean or median	Female	Ethnicity	BMI (kg/m²) Mean or median	Obesity	Smoker	Alcohol drinking	Family history of heart disease	Dyslipidemia	Hypertension	Diabetes	Absence of cardiac RF	≥3 cardiac RF
Eastern Mediterranean region													
Benahmed et al 2018 (n=7)	58.0 (28.0 – 76.0)	1 (14.3%)	-	-	4 (57.1%)	2 (28.6%)	-	-	-	2 (28.6%)	1 (14.3%)	-	-
Daoulah et al 2021 (n=83)	44.0 (37.0 – 55.0)	42 (50.6%)	Arab: 37 (44.6%)	27 (24.8-30)	-	37 (44.6%)	-	-	32 (38.5%)	26 (31.3%)	21 (25.3%)	-	-
Almasi et al 2022 (n=15)	48.2±6.3	15 (100%)	-	30.9±4.7	-	0	-	3 (20.0%)	9 (60.0%)	8 (53.3%)	6 (40.0%)	0	2 (13.3%)
South-East Asia region													
Sharma et al 2016 (n=5)	33.0 (18.0 – 56.0)	1 (20.0%)	-	-	-	1 (20.0%)	-	-	-	2 (40.0%)	2 (40.0%)	1 (20.0%)	-
Valappil et al 2018 (n=16)	51.0±9.14	2 (12.5%)	-	-	-	6 (37.5%)	-	2 (12.5%)	5 (31.3%)	5 (31.3%)	6 (37.5%)	-	-
Western Pacific Region													
Rashid et al 2016 (n=21)	53.3±8.8	20/21 (95.2%)	-	-	-	3/21 (14.3%)	-	5/21 (23.8%)	10/21 (47.6%)	10/21 (47.6%)	1/21 (4.8%)	-	-
McGrath-Cadell et al 2016 (n=40)	45.0±10.0	38 (95.0%)	-	-	-	3 (8.0%)	-	11 (28.0%)	4 (10.0%)	7 (18.0%)	2 (5.0%)	24 (60.0%)	-
Adams et al 2018 (n=22)	48.7±10.7	17 (77.3%)	-	24.9 (22.3 – 30.1)	-	12 (54.5%)	-	7 (31.8%)	6 (27.3%)	9 (40.9%)	0	-	-
Yuvaraj et al 2020 (n=11)	52.4±11.1	9 (81.8%)	-	-	-	-	-	-	-	-	-	-	-
Fahey et al 2021 (n=85)	51.0±11.0	85 (100%)	-	-	-	-	14 (16.5%)	-	30 (35.0%)	34 (40.0%)	4 (5.0%)	-	-
Murphy et al 2022 (n=30)	52.2±9.5	27 (90.0%)	-	-	-	-	-	-	-	-	-	-	-
Murphy et al 2023 (n=35)	53.7±10.6	33 (94.3%)	-	-	9 (25.7%)	-	-	-	4 (11.4%)	7 (20.0%)	1 (2.9%)	-	-
Murphy et al 2024 (n=310)	55.6±9.0	295 (95.2%)	-	-	33 (10.6%)	6 (1.9%)	-	-	39 (12.6%)	92 (29.7%)	8 (2.6%)	-	-

WHO region Study	Age (year) Mean or median	Female	Ethnicity	BMI (kg/m²) Mean or median	Obesity	Smoker	Alcohol drinking	Family history of heart disease	Dyslipidemia	Hypertension	Diabetes	Absence of cardiac RF	≥3 cardiac RF
Dang et al 2023 (n=172)	52.0 (46.0 – 58.0) (n=170)	164 (95.3%)	Caucasian 159 (92.4%)<br><br>Aboriginal 4 (2.3%)<br><br>Asian 3 (1.7%)<br><br>African 1 (0.6%) (n=167)	-	-	35 (20.3%)	-	41 (23.8%)	7 (4.1%)	31 (18.0%)	3 (1.7%)	-	-
Tarr et al 2022 (n=91)	45.4	83 (91.2%)	-	26.4	-	-	-	66 (72.5%)	13 (14.3%)	17 (18.7%)	3 (3.3%)	-	-
Tarr et al 2024 (n=200)	47.2±9.4	188 (94.0%)	-	26.9±5.6 (n=195)	-	-	-	-	28 (14.0%)	44 (22.0%)	-	-	-
McAlister et al 2021 (n=113)	54.0 (49.0 – 63.0)	100 (88.0%)	Caucasia 92 (81.0%)<br><br>Polynesian 17 (15.0%)<br><br>Asian 3 (3.0%)	-	-	19 (17.0%)	-	-	31 (27.0%)	33 (29.0%)	1 (1.0%)	54 (48.0%)	1 (1.0%)
Wong et al 2022 (n=32)	50.5±8.8	21 (65.6%)	European 27 (84.4%)<br><br>Asian 3 (9.4%)<br><br>Mori	-	-	1 (3.1%)	-	6 (18.8%)	15 (46.9%)	8 (25.0%)	0	-	-

WHO region<br>Study	Age (year)<br>Mean or median	Female	Ethnicity	BMI (kg/m²)<br>Mean or median	Obesity	Smoker	Alcohol drinking	Family history of heart disease	Dyslipidemia	Hypertension	Diabetes	Absence of cardiac RF	≥3 cardiac RF
			2 (6.3%)										
Uribe et al 2015 (n=8)	Mean 43.5	4 (50.0%)	Caucasian 6 (75.0%)<br><br>Black 2 (25.0%)	-	-	-	-	-	2 (25.0%)	3 (37.0%)	2 (25.0%)	-	-
Meng et al 2017 (n=21)	44.4±4.1	21 (100%)	-	-	-	1 (5.0%)	-	-	-	7 (33.0%)	0	-	-
Sun et al 2019 (n=85)	55.0±13.6	15 (17.6%)	-	-	-	32 (37.6%)	-	-	29 (34.1%)	44 (51.8%)	24 (28.2%)	-	-
Liu et al 2019 (n=118)	57.4±10.3	16 (13.6%)	-	26.0±3.2	-	82 (69.5%)	54 (45.8%)	-	51 (43.2%)	74 (62.7%)	44 (37.3%)	-	-
Hui et al 2020 (n=70)	50.8±11.5	52 (74.3%)	-	-	-	17 (24.3%)	-	-	25 (35.7%)	40 (57.1%)	8 (11.4%)	0 or 1 RF 43 (61.0%)	-
Chang et al 2022 (n=30)	51.8±9.5	20 (66.7%)	-	-	-	8 (26.7%)	-	-	3 (10.0%)	16 (53.3%)	2 (6.7%)	-	-
Ma et al 2023 (n=81)	56.8±12.8	55 (67.9%)	-	-	-	18 (22.2%)	-	-	22 (27.2%)	40 (49.4%)	16 (19.8%)	-	-
Tokura et al 2014 (n=10)	46.0±17.0	9 (90.0%)	-	-	-	4 (40.0%)	-	-	1 (10.0%)	2 (20.0%)	0	7 (70.0%)	0
Nakashima et al 2016 (n=63)	46.0±10.0	59 (94.0%)	-	22.4±4.2	-	20 (32.0%)	-	5 (8.0%)	14 (23.0%)	21 (33.0%)	0	-	-
Nishiguchi et al 2016 (n=13)	67.3±13.3	7 (54.0%)	-	-	3 (23.0%)	1 (8.0%)	-	1 (8.0%)	4 (31.0%)	7 (54.0%)	4 (31.0%)	-	-
Nishiguchi et al 2017 (n=12)	63.1±11.8	7 (58.0%)	-	-	2 (17.0%)	6 (50.0%)	-	0	6 (50.0%)	7 (58.0%)	4 (33.0%)	-	-<br>No. of RF 2.2±1.3
Kubota et al 2020 (n=7)	50.0±6.0	6 (86.0)	-	-	-	1 (14.3%)	-	-	2 (28.6%)	0	0	-	-
Inohara et al 2020 (n=322)	52.8±13.5	322 (100%)	-	22.6±4.0	-	-	-	-	129 (40.1%)	189 (58.7%)	10 (3.1%)	-	-
Inoue et al 2021 (n=19)	48.7±7.1	19 (100%)	-	23.0±4.2	-	2 (10.5%)	-	-	8 (42.1%)	8 (42.1%)	1 (5.3%)	-	-
Taguchi et al 2023 (n=16)	58.4 (43.0 – 79.0)	14 (87.5%)	-	-	-	2 (12.5%)	-	-	4 (25.0%)	11 (69.0%)	-	3 (18.75%)	0

WHO region<br>Study	Age (year)<br>Mean or median	Female	Ethnicity	BMI (kg/m^2)<br>Mean or median	Obesity	Smoker	Alcohol drinking	Family history of heart disease	Dyslipidemia	Hypertension	Diabetes	Absence of cardiac RF	≥3 cardiac RF
Kim et al 2021 (n=13)	52.1±5.8	13 (100%)	-	21.4±8.2	-	3 (23.1%)	-	-	1 (7.7%)	3 (23.1%)	1 (7.7%)	-	-
Turkey													
Celik et al 2001 (n=9)	55.7 (45.0 – 73.0)	2 (22.2%)	-	-	4 (44.4%)	7 (77.8%)	-	-	3 (33.3%)	2 (22.2%)	2 (22.2%)	-	-
Unal et al 2008 (n=6)	48.0 (35.0 – 61.0)	5 (83.3%)	-	-	-	2 (33.3%)	-	-	2 (33.3%)	5 (83.3%)	2 (33.3%)	1 (16.7%)	1 (16.7%)
Canga et al 2018 (n=22)	47.0±12.3	11 (50.0%)		-	-	7 (32.0%)	-	-	1 (5.0%)	5 (25.0%)	3 (16.0%)	-	-
Özkan et al 2023 (n=49)	44.0 (36.0 – 50.0)	49 (100%)	-	-	-	7 (14.3%)	8 (16.3%)	8 (16.3%)	6 (12.2%)	5 (10.2%)	1 (2.0%)	-	-

Abbreviations: BMI, body mass index; CAD, coronary artery disease; RF, risk factor(s).

Table S9. Co-morbidities at baseline

WHO region<br>Study	CCI<br>0	CCI<br>1	CCI<br>2	CCI<br>≥3	CAD	Prior MI	Prior PCI	Prior CABG	Prior CVA/TIA	CKD	Dialysis	Polycystic kidney disease	Hypothyroidism	Prior SCAD
Eastern Mediterranean region														
Benahmed et al 2018 (n=7)	-	-	-	-	-	-	-	-	-	-	-	-	-	-
Daoulah et al 2021 (n=83)	-	-	-	-	-	-	-	-	-	-	-	-	6 (7.2%)	-
Almasi et al 2022 (n=15)	-	-	-	-	-	-	0	0	0	-	-	-	-	-
South-East Asia region														
Sharma et al 2016 (n=5)	-	-	-	-	-	-	-	-	-	-	-	-	-	-
Valappil et al 2018 (n=16)	-	-	-	-	-	-	-	-	-	-	-	-	-	-
Western Pacific Region														
Rashid et al 2016 (n=21)	-	-	-	-	-	-	-	-	-	-	-	-	-	Recurrent SCAD: 3/11 (14.3%)
McGrath-Cadell et al 2016 (n=40)	-	-	-	-	-	-	-	-	-	-	-	1 (2.5%)	-	-
Adams et al 2018 (n=22)	-	-	-	-	-	-	-	-	-	-	-	-	-	-
Yuvaraj et al 2020 (n=11)	-	-	-	-	-	-	-	-	-	-	-	-	-	-
Fahey et al 2021 (n=85)	-	-	-	-	-	-	-	-	-	-	-	-	-	-
Murphy et al 2022 (n=30)	-	-	-	-	-	-	-	-	-	-	-	-	-	-
Murphy et al 2023 (n=35)	-	-	-	-	-	-	-	-	0	1 (2.9%)	-	-	Thyrpoid condition: 2 (5.7%)	-
Murphy et al 2024 (n=310)	-	-	-	-	-	-	-	-	8 (2.6%)	-	-	-	-	No. of episode:

WHO region<br>Study	CCI<br>0	CCI<br>1	CCI<br>2	CCI<br>≥3	CAD	Prior MI	Prior PCI	Prior CABG	Prior CVA/TIA	CKD	Dialysis	Polycystic kidney disease	Hypothyroidism	Prior SCAD
														1: 241 (77.7%)<br>2: 46 (14.8%)<br>3: 16 (5.2%)<br>4: 4 (1.3%)<br>5: 3 (1.0%)
Dang et al 2023 (n=172)	-	-	-	-	-	-	-	-	-	-	-	-	-	Any – No. of episode:<br>1: 132 (77.6%)<br>2: 27 (15.9%)<br>3: 10 (5.9%)<br>4: 1 (0.6%)<br>(n=170)
Tarr et al 2022 (n=91)	-	-	-	-	-	Heart disease 5 (5.5%)	-	-	2 (2.2%)	1 (1.1%)	-	-	-	-
Tarr et al 2024 (n=200)	-	-	-	-	-	-	-	-	-	-	-	-	-	-
McAlister et al 2021 (n=113)	-	-	-	-	-	-	-	-	-	-	-	-	-	-
Wong et al 2022 (n=32)	-	-	-	-	-	-	-	-	-	-	-	-	-	-
Uribe et al 2015 (n=8)	-	-	-	-	0	0	0	0	0	-	-	-	-	0
Meng et al 2017 (n=21)	-	-	-	-	-	-	-	-	-	-	-	-	-	-
Sun et al 2019 (n=85)	-	-	-	-	-	16 (18.8%)	-	-	7 (8.2%)	-	-	-	4 (4.7%)	-

WHO region<br>Study	CCI<br>0	CCI<br>1	CCI<br>2	CCI<br>≥3	CAD	Prior MI	Prior PCI	Prior CABG	Prior CVA/TIA	CKD	Dialysis	Polycystic kidney disease	Hypothyroidism	Prior SCAD
Liu et al 2019 (n=118)	-	-	-	-	85 (72.0%)	39 (33.1%)	-	-	16 (13.6%)	-	-	-	-	-
Hui et al 2020 (n=70)	-	-	-	-	-	4 (5.7%)	-	-	-	-	-	-	-	-
Chang et al 2022 (n=30)	-	-	-	-	-	0	0	0	-	-	-	-	-	-
Ma et al 2023 (n=81)	-	-	-	-	-	-	-	-	-	-	-	-	-	-
Tokura et al 2014 (n=10)	-	-	-	-	-	-	-	-	-	-	-	-	-	-
Nakashima et al 2016 (n=63)	-	-	-	-	-	-	-	-	-	0	-	-	-	-
Nishiguchi et al 2016 (n=13)	-	-	-	-	-	-	-	-	-	-	-	-	-	-
Nishiguchi et al 2017 (n=12)	-	-	-	-	-	1 (8.0%)	1 (8.0%)-	-	-	1 (8.0%)	1 (8.0%)	-	-	-
Kubota et al 2020 (n=7)	-	-	-	-	-	-	-	-	-	-	-	-	-	-
Inohara et al 2020 (n=322)	229 (71.1%)	7 (2.2%)	82 (25.5%)	4 (1.2%)	-	14 (4.3%)	-	-	-	2 (0.6%)	-	-	-	-
Inoue et al 2021 (n=19)	-	-	-	-	-	1 (5.3%)	0	-	0	0	0	-	-	-
Taguchi et al 2023 (n=16)	-	-	-	-	-	-	-	-	-	-	-	-	-	-
Kim et al 2021 (n=13)	-	-	-	-	-	-	-	-	-	-	-	-	-	-
Turkey														
Celik et al 2001 (n=9)	-	-	-	-	-	4 (44.4%)	-	-	-	-	-	-	-	-
Unal et al 2008 (n=6)	-	-	-	-	3 (50.0%)	-	-	-	-	-	-	-	-	-
Canga et al 2018 (n=22)	-	-	-	-	-	-	-	-	-	-	-	-	-	-
Özkan et al 2023 (n=49)	-	-	-	-	-	1 (2.0%)	-	-	-	-	-	-	-	-

Abbreviations: CABG, coronary artery bypass grafting; CCI, Charlson Comorbidity Index; CKD, chronic kidney disease; CVA, cerebrovascular attack; MI, myocardial infarction; PCI, percutaneous coronary intervention; SCAD, spontaneous coronary artery dissection; TIA, transient ischemic attack.

Table S10. Predisposing conditions and precipitating factors or preceding events

WHO region Study	Systemic inflammatory disorder	Autoimmune disease	FMD	Carotid dissection	Extra-coronary aneurysm	Depression	Anxiety	Migraine	Physical stress	Emotional stress	Combined stress	Valsalva-type activities	IV Methamphetamine use	Vasoconstrictor drug use
Eastern Mediterranean region														
Benahmed et al 2018 (n=7)	-	-	-	-	-	-	-	-	-	-	-	-	-	-
Daoulah et al 2021 (n=83)	-	-	-	-	-	12 (14.5%)	28 (33.7%)	19 (22.9%)	9 (10.8%)	33 (39.8%)	7 (8.4%)	-	-	-
Almasi et al 2022 (n=15)	-	-	-	-	-	-	-	-	-	-	-	-	-	-
South-East Asia region														
Sharma et al 2016 (n=5)	-	-	-	-	-	-	-	-	Vigorous 1 (20.0%)	-	-	-	-	-
Valappil et al 2018 (n=16)	-	-	-	-	-	-	-	-	Severe exertion 8 (50.0%)	-	-	-	-	-
Western Pacific Region														
Rashid et al 2016 (n=21)	-	-	-	-	3/11 (27.3%)	-	-	-	-	-	Any: 5/21 (23.8%)	-	-	-
McGrath-Cadell et al 2016 (n=40)	-	-	-	1 (2.5%)	2 (5.0%)	-	-	-	5 (12.5%)	4 (10.0%)	-	-	1 (2.5%)	2 (5.0%)
Adams et al 2018 (n=22)	-	-	-	-	-	Anxiety, Depression or Others 11 (52.4%)		-	-	Extreme exertion 3 (13.6%)	-	-	0	-
Yuvaraj et al 2020 (n=11)	-	-	-	-	-	-	-	-	-	-	-	-	-	-
Fahey et al 2021 (n=85)	-	-	-	-	-	-	-	-	-	-	-	-	-	-
Murphy et al 2022 (n=30)	-	-	-	-	-	-	-	-	-	-	-	-	-	-

WHO region Study	Systemic inflammatory disorder	Autoimmune disease	FMD	Carotid dissection	Extra-coronary aneurysm	Depression	Anxiety	Migraine	Physical stress	Emotional stress	Combined stress	Valsalva-type activities	IV Methamphetamine use	Vasoconstrictor drug use
Murphy et al 2023 (n=35)	-	-	-	-	-	14 (40.0%)	17 (48.6%)	-	-	-	-	-	-	-
Murphy et al 2024 (n=310)	21 (1.6%)	-	53 (17.1%)	-	-	60/287 (20.9%)	60/290 (20.7%)	102 (32.9%)	-	-	-	-	-	-
Dang et al 2023 (n=172)	-	Any 28 (16.3%)		-	-	34 (21.4%)	31 (19.5%)	-	-	-	-	-	-	-
Tarr et al 2022 (n=91)	-	-	12/34 screened patients (27.9%)	6 (6.6%)	4 (4.4%)	Depression or Anxiety 21 (23.1%)		42 (46.2%)	14 (15.4%)	Acute: 4 (4.4%) Chronic: 46 (50.0%)	-	-	-	-
Tarr et al 2024 (n=200)	-	-	-	-	-	Depression or Anxiety 70 (35.0%)		98 (49.0%)	34 (17.0%)	117 (58.5%)	-	-	-	-
McAlister et al 2021 (n=113)	-	-	-	-	-	-	-	-	-	-	-	-	-	-
Wong et al 2022 (n=32)	-	-	-	-	-	-	-	-	-	-	-	-	-	-
Uribe et al 2015 (n=8)	-	-	0	-	-	-	-	-	-	-	-	-	-	-
Meng et al 2017 (n=21)	-	0	1 (5.0%)	-	-	0	-	-	-	-	-	-	-	-
Sun et al 2019 (n=85)	-	-	-	-	-	-	-	-	-	-	-	-	-	-
Liu et al 2019 (n=118)	-	-	-	-	-	-	-	-	-	-	-	-	-	-
Hui et al 2020 (n=70)	-	-	32 (45.8%)	-	-	Depression or Anxiety 11 (15.7%)		-	-	-	-	-	-	-
Chang et al 2022 (n=30)	-	-	-	-	-	-	-	-	-	-	-	-	-	-
Ma et al 2023 (n=81)	-	-	-	-	-	-	-	-	-	-	-	-	-	-
Tokura et al 2014 (n=10)	-	-	-	-	-	-	-	-	-	-	-	-	-	-

WHO region Study	Systemic inflammatory disorder	Autoimmune disease	FMD	Carotid dissection	Extra-coronary aneurysm	Depression	Anxiety	Migraine	Physical stress	Emotional stress	Combined stress	Valsalva-type activities	IV Methamphetamine use	Vasoconstrictor drug use
Nakashima et al 2016 (n=63)	-	-	5 (20.0%)	-	-	-	-	-	Extreme 6 (10.0%)	18 (29.0%)	-	-	-	-
Nishiguchi et al 2016 (n=13)	-	-	-	-	-	-	-	-	-	-	-	-	-	-
Nishiguchi et al 2017 (n=12)	-	-	-	-	-	-	-	-	-	-	-	-	-	-
Kubota et al 2020 (n=7)	-	-	-	-	-	-	-	-	-	-	-	-	-	-
Inohara et al 2020 (n=322)	-	-	-	-	-	-	-	-	-	-	-	-	-	-
Inoue et al 2021 (n=19)	1 (5.3%)	-	-	-	-	-	-	-	2 (10.5%)	2 (10.5%)	4 (21.0%)	2 (10.5%)	-	-
Taguchi et al 2023 (n=16)	-	-	Brachial artery 6/9 (66.7%)	-	-	-	-	-	-	-	-	-	-	-
Kim et al 2021 (n=13)	-	-	-	-	-	-	-	-	-	-	-	-	-	-
Turkey														
Celik et al 2001 (n=9)	-	-	-	-	-	-	-	-	-	-	-	-	-	-
Unal et al 2008 (n=6)	-	-	-	-	-	-	-	-	-	-	-	-	-	-
Canga et al 2018 (n=22)	-	-	-	-	-	-	-	-	-	-	-	-	-	-
Özkan et al 2023 (n=49)	-	-	Connective tissue disease 7 (14.3%)	-	-	-	-	-	-	-	-	-	-	-

Abbreviations: FMD, fibromuscular dysplasia disease; IV, intravenous; OCP, oral contraceptive pills.

Tables S11. Female sex related characteristics

WHO region<br>Study	Prior SCAD with pregnancy	Multiparous (≥4 births)	Menopause	On contraceptives	On postmenopausal hormonal therapy	In pregnancy period	In postpartum period
Eastern Mediterranean region							
Benahmed et al 2018 (n=7)	-	-	-	-	-	-	-
Daoulah et al 2021 (n=83)	-	9/42 (21.4%)	12/42 (28.5%)	Any<br>5/42 (12.0%)		-	-
Almasi et al 2022 (n=15)	-	-	-	-	-	-	-
South-East Asia region							
Sharma et al 2016 (n=5)	-	-	-	-	-	1 (20.0%)	-
Valappil et al 2018 (n=16)	-	-	2 (12.5%)	-	-	-	-
Western Pacific Region							
Rashid et al 2016 (n=21)	-	-	-	-	-	-	-
McGrath-Cadell et al 2016 (n=40)	-	-	-	-	-	-	3 (7.5%)
Adams et al 2018 (n=22)	-	-	5 (22.2%)	On OCP or in peripartum period 4 (16.7%)	-	-	-
Yuvaraj et al 2020 (n=11)	-	-	-	-	-	-	-
Fahey et al 2021 (n=85)	-	-	-	-	-	-	-
Murphy et al 2022 (n=30)	-	-	-	-	-	-	-
Murphy et al 2023 (n=35)	-	-				-	-
Murphy et al 2024 (n=310)	-	-	-	-	-	-	-
Dang et al 2023 (n=172)	-	-	-	-	-	-	-
Tarr et al 2022 (n=91)	10/83 females (12.0%)	Number of births 2.1 (n=83)	-	-	-	-	-

WHO region<br>Study	Prior SCAD with pregnancy	Multiparous (≥4 births)	Menopause	On contraceptives	On postmenopausal hormonal therapy	In pregnancy period	In postpartum period
Tarr et al 2024 (n=200)	-	-	-	-	-	-	-
McAlister et al 2021 (n=113)	-	-	-	-	-	-	-
Wong et al 2022 (n=32)	-	-	-	-	-	-	-
Uribe et al 2015 (n=8)	-	-	-	2 (25.0%)	-	-	3 (37.0%)
Meng et al 2017 (n=21)	-	-	11 (52.0%)	0	-	0	0
Sun et al 2019 (n=85)	2 (2.4%)	-	-	-	-	-	-
Liu et al 2019 (n=118)	-	-	-	-	-	-	-
Hui et al 2020 (n=70)	-	-	29 (56.9%)	-	-	-	Peripartum<br>5 (9.8%)
Chang et al 2022 (n=30)	-	13 (43.3%)	-	-	-	-	-
Ma et al 2023 (n=81)	-	-	-	-	-	-	-
Tokura et al 2014 (n=10)	-	-	-	-	-	1 (10.0%)	1 (10.0%)
Nakashima et al 2016 (n=63)	-	-	Early<br>3 (5.0%)	0	1 (2.0%)	-	Peripartum<br>5 (8.0%)
Nishiguchi et al 2016 (n=13)	-	-	-	-	-	-	-
Nishiguchi et al 2017 (n=12)	-	-	-	-	-	-	-
Kubota et al 2020 (n=7)	-	-	-	-	-	-	-
Inohara et al 2020 (n=322)	-	-	-	-	-	-	-
Inoue et al 2021 (n=19)	-	-	-	Any:<br>1 (5.3%)		0	0
Taguchi et al 2023 (n=16)	-	-	-	-		-	-
Kim et al 2021 (n=13)	-	-	3 (23.1%)	-	-	0	-

WHO region<br>Study	Prior SCAD with pregnancy	Multiparous (≥4 births)	Menopause	On contraceptives	On postmenopausal hormonal therapy	In pregnancy period	In postpartum period
Turkey							
Celik et al 2001 (n=9)	-	-	-	-	-	0	-
Unal et al 2008 (n=6)	-	-	-	-	-	-	-
Canga et al 2018 (n=22)	-	-	-	-	-	-	-
Özkan et al 2023 (n=49)	-	-	-	-	-	0	-

Table S12. Clinical manifestation and presentation upon hospital admission

WHO regions Study	Chest pain	Dyspnea	Asymptomatic	Killip 0 & I	Killip II & III	Killip IV	STEMI	NSTEMI	UA	Stable angina	CA or VA	HF
Eastern Mediterranean region												
Benahmed et al 2018 (n=7)	-	-	-	-	-	-	4 (57.1%)	2 (28.6%)	-	Exertional 1 (14.3%)		
Daoulah et al 2021 (n=83)	-	-	-	-	-	-	41 (49.4%)	39 (46.9%)	3 (3.6%)	-	VA 10 (12.0%)	-
Almasi et al 2022 (n=15)	-	-	-	-	-	-	15 (100%)	-	-	-	-	-
South-East Asia region												
Sharma et al 2016 (n=5)	-	-	-	-	-	-	5 (100%)	-	-	-	-	-
Valappil et al 2018 (n=16)	-	-	-	-	-	-	7 (43.75%)	NSTEMI or UA 3 (18.75%)		6 (37.5%)	-	-
WHO Western Pacific Region												
Rashid et al 2016 (n=21)	-	-	-	-	-	-	8/23 (34.8%)	13/23 (56.5%)	2/23 (8.7%)	-	-	-
McGrath-Cadell et al 2016 (n=40)	-	-	-	-	-	-	12 (30.0%)	26 (65.0%)	-	-	5 (12.5%)	-
Adams et al 2018 (n=22)	-	-	-	-	-	-	-	-	-	-	-	-
Yuvaraj et al 2020 (n=11)	-	-	-	-	-	-	-	-	-	-	-	-
Fahey et al 2021 (n=85)	2 (2.3%)	-	-	-	-	-	37 (43.5%)	43 (50.6%)	-	-	Arrest 3 (3.5%)	-
Murphy et al 2022 (n=30)	-	-	-	-	-	-	-	-	-	-	-	-
Murphy et al 2023 (n=35)	-	-	-	-	-	-	-	-	-	-	-	-
Murphy et al 2024 (n=310)	-	-	-	-	-	-	-	-	-	-	-	-
Dang et al 2023 (n=172)	119 (69.2%)	45 (26.2%)	-	-	-	-	-	-	-	-	-	-
Tarr et al 2022 (n=91)	-	-	-	-	-	-	-	-	-	-	-	-
Tarr et al 2024 (n=200)	-	-	-	-	-	-	-	-	-	-	-	-

WHO regions Study	Chest pain	Dyspnea	Asymptomatic	Killip 0 & I	Killip II & III	Killip IV	STEMI	NSTEMI	UA	Stable angina	CA or VA	HF
McAlister et al 2021 (n=113)	-	-	-	-	-	-	32 (28.0%)	81 (72.0%)	-	-	6 (5.0%)	-
Wong et al 2022 (n=32)	24 (89.0%)	12 (44.0%)	-	-	-	-	15 (46.9%)	17 (53.1%)	-	-	-	-
Uribe et al 2015 (n=8)	-	-	-	-	-	-	0	5 (63.0%)	3 (37.0%)	-	-	-
Meng et al 2017 (n=21)	-	-	-	-	-	-	12/21 (57.0%)	9/21 (43.0%)	-	-	-	-
Sun et al 2019 (n=85)	-	-	-	-	-	-	27 (32.1%)	9 (10.8%)	48 (57.1%)	-	VA: 2 (4.1%)	-
Liu et al 2019 (n=118)	-	-	9 (7.6%)	-	-	-	28 (23.7%)	28 (23.7%)	32 (27.1%)	11 (9.3%)	-	10 (8.5%)
Hui et al 2020 (n=70)	64 (91.4%)	-	-	-	> I 17 (22.9%)		29 (41.4%)	NSTEMI or UA 41 (58.6%)		-	-	-
Chang et al 2022 (n=30)	-	-	-	-	-	-	6 (20.0%)	22 (73.3%)	2 (6.7%)	-	-	-
Ma et al 2023 (n=81)	-	-	-	-	-	-	-	-	-	-	-	-
Tokura et al 2014 (n=10)	-	-	-	-	-	-	9 (90.0%)	-	1 (10.0%)	-	-	-
Nakashima et al 2016 (n=59)	-	-	-	-	-	-	55 (87.0%)	8 (13.0%)	-	-	CA or CS 10 (16.0%)	-
Nishiguchi et al 2016 (n=13)	-	-	-	-	-	-	8 (62.0%)	-	-	-	-	-
Nishiguchi et al 2017 (n=12)	-	-	-	-	-	-	11 (92.0%)	-	-	-	-	-
Kubota et al 2020 (n=7)	-	-	-	-	-	-	3 (42.8%)	4 (57.1%)	-	-	-	-
Inohara et al 2020 (n=322)	-	-	-	0: 25 (7.9%) I: 208 (66.0%)	II: 49 (15.6%) III: 10 (3.2%)	23 (7.3%)	-	-	-	-	-	-
Inoue et al 2021 (n=19)	-	-	-	-	-	-	11 (57.8%)	4 (21.0%)	4 (21.0%)	-	-	-
Taguchi et al 2023 (n=16)	-	-	-	-	-	-	8 (50.0%)	8 (50.0%)	-	-	-	-
Kim et al 2021 (n=13)	12 (92.3%)	1 (7.7%)	-	-	III: 0	0	7 (53.8%)	6 (46.2%)	-	-	-	-

WHO regions Study	Chest pain	Dyspnea	Asymptom atic	Killip 0 & I	Killip II & III	Killip IV	STEMI	NSTEMI	UA	Stable angina	CA or VA	HF
Turkey												
Celik et al 2001 (n=9)	-	-	-	-	-	-	4 (44.4%)	-	4 (44.4%)	2 (22.2%)	-	1 (11.1%) plus UA
Unal et al 2008 (n=6)	-	-	-	-	-	-	5 (83.3%)	-	1 (16.7%)	-	-	-
Canga et al 2018 (n=22)	-	-	-	-	-	-	4 (18.2%)	NSTEMI or UA 18 (81.8%)		-	-	-
Özkan et al 2023 (n=49)	-	-	-	-	-	-	13 (26.5%)	33 (67.3%)	3 (6.1%)	-	-	-

Abbreviations: CA, cardiac arrest; CS, cardiogenic shock; HF, heart failure; NSTEMI, non-ST segment elevation myocardial infarction; STEMI, ST segment elevation myocardial infarction; UA, unstable angina; VA, ventricular arrhythmia.

Table S13. Laboratory findings

WHO region Study	Hb	SCr	ALT	Glucose	TC	TG	HDL-C	LDL-C	hs-CRP	CK-MB	cTnT	cTnI	NT-pro-BNP	D-dimer
Eastern Mediterranean region														
Benahmed et al 2018 (n=7)	-	-	-	-	-	-	-	-	-	-	-	-	-	-
Daoulah et al 2021 (n=83)	-	-	-	-	-	-	-	-	-	33 (5.8 - 84.5) µg/L	Troponin 2.7 (0.05 – 20.0) ng/L	-	-	-
Almasi et al 2022 (n=15)	-	-	-	-	-	-	-	-	-	-	-	-	-	-
South-East Asia region														
Sharma et al 2016 (n=5)	-	-	-	-	-	-	-	-	-	-	-	-	-	-
Valappil et al 2018 (n=16)	-	-	-	-	-	-	-	-	-	-	-	-	-	-
Western Pacific Region														
Rashid et al 2016 (n=21)	-	-	-	-	4.3±0.6 mmol/L	-	1.7±0.4 mmol/L	2.5±0.6 mmol/L	-	-	-	-	-	-
McGrath-Cadell et al 2016 (n=40)	-	-	-	-	-	-	-	-	-	-	-	-	-	-
Adams et al 2018 (n=22)	-	-	-	-	-	-	-	-	-	-	-	-	-	-
Yuvaraj et al 2020 (n=11)	-	-	-	-	-	-	-	-	-	-	-	-	-	-
Fahey et al 2021 (n=85)	-	-	-	-	-	-	-	-	-	Peak CK 677±701 U/L	-	Peak 11.9± 15.7 µg/L	-	-
Murphy et al 2022 (n=30)	-	-	-	-	-	-	-	-	-	-	-	-	-	-
Murphy et al 2023 (n=35)	-	-	-	-	-	-	-	-	-	-	-	-	-	-
Murphy et al 2024 (n=310)	-	-	-	-	-	-	-	-	-	-	-	-	-	-
Dang et al 2023 (n=172)	-	-	-	-	-	-	-	-	-	-	-	-	-	-

WHO region Study	Hb	SCr	ALT	Glucose	TC	TG	HDL-C	LDL-C	hs-CRP	CK-MB	cTnT	cTnI	NT-pro-BNP	D-dimer
Tarr et al 2022 (n=91)	-	-	-	-	-	-	-	-	-	-	-	-	-	-
Tarr et al 2024 (n=200)	-	-	-	-	-	-	-	-	-	-	-	-	-	-
McAlister et al 2021 (n=113)	-	-	-	-	-	-	-	-	-	-	-	-	-	-
Wong et al 2022 (n=32)	-	-	-	-	-	-	-	-	-	-	-	-	-	-
Uribe et al 2015 (n=8)	-	-	-	-	-	-	-	-	-	-	-	-	-	-
Meng et al 2017 (n=21)	121.9± 14.4 g/L	51.5± 11.1 µmol/L	29.6±18.7 U/L	5.2±1.2 mmol/L	3.6±0.9 mmol/L	1.3±0.7 mmol/L	1.2±0.2 mmol/L	2.0±0.7 mmol/L	-	-	-	-	-	-
Sun et al 2019 (n=85)	-	-	-	-	-	-	-	-	-	-	-	-	-	-
Liu et al 2019 (n=118)	-	-	-	-	-	-	-	-	-	-	-	-	-	-
Hui et al 2020 (n=70)	-	64.1 (55.7 - 74.4) µmol/L	-	-	-	-	-	-	-	-	-	3.2 (0.2 - 12.2) ng/mL	-	-
Chang et al 2022 (n=30)	-	-	-	-	-	-	-	-	-	-	-	-	-	-
Ma et al 2023 (n=81)	-	-	-	-	-	-	-	-	-	17.65±14.69 U/L	0.18± 0.50 mg/mL	-	499.15± 639.84 pg/mL	0.57±1.0 mg/L
Tokura et al 2014 (n=10)	-	-	-	-	-	130± 82.0 mmol/L	48.0± 20.0 mmol/L	88.0± 29.0 mmol/L	-	-	-	-	-	-
Nakashima et al 2016 (n=59)	-	-	-	-	-	-	-	-	-	-	-	-	-	-
Nishiguchi et al 2016 (n=13)	-	-	-	-	-	-	-	-	0.14 (IQR: 0.29) mg/dL	Peak 90.0 (IQR: 142) IU/L	-	-	-	-
Nishiguchi et al 2017 (n=12)	-	-	-	-	-	-	-	-	0.20±0.19 mg/dL	Peak	-	-	-	-

WHO region Study	Hb	SCr	ALT	Glucose	TC	TG	HDL-C	LDL-C	hs-CRP	CK-MB	cTnT	cTnI	NT-pro-BNP	D-dimer
										53.0 (IQR: 257) IU/L				
Kubota et al 2020 (n=7)	-	-	-	-	-	-	-	-	-	-	-	-	-	-
Inohara et al 2020 (n=322)	-	-	-	-	-	-	-	-	-	-	-	-	-	-
Inoue et al 2021 (n=19)	-	-	-	-	-	-	-	-	-	-	-	-	-	-
Taguchi et al 2023 (n=16)	-	-	-	-	-	-	-	-	-	-	-	-	-	-
Kim et al 2021 (n=13)	-	-	-	-	168 (147 - 197) mg/dL	78.0 (39.0 - 109) mg/dL	54.0 (46.0 – 69.0) mg/dL	99.0 (67.0 - 127) mg/dL	0.8 (0.1 - 2.9) mg/dL	Peak 38.2 (2.6 -145.5) ng/dL	-	Peak 5.1 (1.1 - 21.1) ng/dL	-	-
Turkey														
Celik et al 2001 (n=9)	-	-	-	-	-	-	-	-	-	-	-	-	-	-
Unal et al 2008 (n=6)	-	-	-	-	-	-	-	-	-	-	-	-	-	-
Canga et al 2018 (n=22)	-	-	-	-	-	-	-	-	0.70 (0.13 - 2.70) mg/dl	-	-	-	-	-
Özkan et al 2023 (n=49)	12.1±1.0 g/dL	0.9±0.1 mg/dL	26.9±5.0 U/L	97.6±9.3 mg/dL	239.8± 33.4 mg/dL	222.5± 38.0 mg/dL	33.0 (19.0 – 44.0)	131.3± 14.9	39.0 (26.0 – 51.0)	-	-	-	-	-

ALT, alanine transaminase; CK-MB, creatine kinase-myocardial band; cTnI, cardiac troponin I; cTnT, cardiac troponin T; Hb, hemoglobin; HDL-C, high-density lipoprotein-cholesterol; hs-CRP, high-sensitivity C-reactive protein; IQR, interquartile range; LDL-C, low-density lipoprotein-cholesterol; NT-pro-BNP, N-terminal pro-B-type natriuretic peptide; SCr, serum creatinine; TC, total cholesterol; TG, triglycerides.

Table S14. Echocardiography parameters and FMD screening

WHO region<br>Study	LVEF (%)	LVEF <50%	LVEF <35%	LVEDD (cm)	RWMA	Ventricular aneurysm		FMD screening			
								Not screened	Positive	Negative	Uncertain
Eastern Mediterranean region											
Benahmed et al 2018 (n=7)	45.6 (15.0 – 64.0)	-	-	-	-	-		-	-	-	-
Daoulah et al 2021 (n=83)	0.45 (0.4-0.55)	-	14 (16.9%)	-	-	-		-	-	-	-
Almasi et al 2022 (n=15)	44.0±8.6	-	-	-	-	-		-	-	-	-
South-East Asia region											
Sharma et al 2016 (n=5)	46.0 (30.0 – 50.0)	-	-	-	-	-		-	-	-	-
Valappil et al 2018 (n=16)	-	-	-	-	-	-		0	-	0	-
Western Pacific Region											
Rashid et al 2016 (n=21)	-	-	-	-	-	-		10/21 (47.6%)	2/21 (9.5%)	-	-
McGrath-Cadell et al 2016 (n=40)	-	-	-	-	-	-		17 (43.0%)	7 (17.5%)	12 (30.0%)	4 (10.0%)
Adams et al 2018 (n=22)	60.0 (50.0 – 65.0)	-	-	-	-	-		-	-	-	-
Yuvaraj et al 2020 (n=11)	-	-	-	-	-	-		-	-	-	-
Fahey et al 2021 (n=85)	-	-	-	-	-	-		-	-		
Murphy et al 2022 (n=30)	-	-	-	-	-	-		-	-	-	-
Murphy et al 2023 (n=35)	-	-	-	-	-	-		-	-	-	-
Murphy et al 2024 (n=310)	-	-	-	-	-	-		-	-	-	-
Dang et al 2023 (n=172)	-	-	-	-	-	-		55/159 (34.6%)	24/104 (23.1%)	Any 80/104 (76.9%)	
Tarr et al 2022 (n=91)	-	-	-	-	-	-		-	-	-	-

WHO region Study	LVEF (%)	LVEF <50%	LVEF <35%	LVEDD (cm)	RWMA	Ventricular aneurysm		FMD screening			
								Not screened	Positive	Negative	Uncertain
Tarr et al 2024 (n=200)	-	-	-	-	-	-		-	-	-	-
McAlister et al 2021 (n=113)	57.0 (50.0 – 62.0)	25 (22.0%)	9 (8.0%)	-	-	-		-	-	-	-
Wong et al 2022 (n=32)	-	-	-	-	-	-		-	-	-	-
Uribe et al 2015 (n=8)	Mean 47.5%	3 (37.0%)	1 (12.0%)	-	-	-	-	-	-	-	-
Meng et al 2017 (n=21)	-	-	-	-	-	-		-	-	-	-
Sun et al 2019 (n=85)	53.3±13.0	25 (29.4%)	-	-	38 (44.7%)	-		-	-	-	-
Liu et al 2019 (n=118)	50.1±9.4	-	-	5.2±0.7	65 (55.1%)	13 (11.0%)		-	-	-	-
Hui et al 2020 (n=70)	-	-	-	-	-	-		-	-	-	-
Chang et al 2022 (n=30)	62.8±5.4	-	-	-	2 (6.7%)	-		-	-	-	-
Ma et al 2023 (n=81)	59.6±8.8	-	-	-	-	-		-	-	-	-
Tokura et al 2014 (n=10)	-	-	-	-	-	-		-	-	-	-
Nakashima et al 2016 (n=59)	-	-	-	-	-	-		38/63 (60.3%)	5/25 (20.0%)	-	-
Nishiguchi et al 2016 (n=13)	-	-	-	-	-	-		-	-	-	-
Nishiguchi et al 2017 (n=12)	-	-	-	-	-	-		-	-	-	-
Kubota et al 2020 (n=7)	-	-	-	-	-	-		-	-	-	-
Inohara et al 2020 (n=322)	-	-	-	-	-	-		-	-	-	-
Inoue et al 2021 (n=19)	-	-	-	-	-	-		-	-	-	-
Taguchi et al 2023 (n=16)	-	-	-	-	-	-		-	-	-	-

WHO region<br>Study	LVEF (%)	LVEF <50%	LVEF <35%	LVEDD (cm)	RWMA	Ventricular aneurysm		FMD screening			
								Not screened	Positive	Negative	Uncertain
Kim et al 2021 (n=13)	54.9±8.9	-	-	-	-	-		-	-	-	-
Turkey											
Celik et al 2001 (n=9)	-	-	-	-	-	-		-	-	-	-
Unal et al 2008 (n=6)	-	-	-	-	-	-		-	-	-	-
Canga et al 2018 (n=22)	Pre-discharge 49.1±12.0										
Özkan et al 2023 (n=49)	55.0 (42.0 – 67.0)	-	-	-	-	-	-	-	-	-	-

Abbreviations: FMD, fibromuscular dysplasia; LVEDD, left ventricular end diastolic diameter; LVEF, left ventricular ejection fraction; RWMA, regional ventricular wall motion abnormality.

Table S15. In-hospital SCAD management

WHO region<br>Study	Medical therapy	CABG	PCI	IVUS	OCT	Aspiration	Conventional balloon	Cutting balloon	Wiring
Eastern Mediterranean region									
Benahmed et al 2018 (n=7)	6 (85.7%)	-	1 (14.3%)	-	-	-	-	-	-
Daoulah et al 2021 (n=83)	33 (39.8%)	6 (7.2%)	44 (53.0%)	-	-	-	-	-	-
Almasi et al 2022 (n=15)	-	-	11 (73.3%)	-	-	-	-	-	-
South-East Asia region									
Sharma et al 2016 (n=5)	5 (100%)	0	0	-	-	-	-	-	-
Valappil et al 2018 (n=16)	-	-	16 (100%)<br>Stents<br>13 (81.2%)	-	-	-	POBA<br>1 (6.2%)	-	16 (100%)
Western Pacific Region									
Rashid et al 2016 (n=21)	-	-	Stents<br>1/26 (3.8%)	-	-	-	1/26 (3.8%)	-	Guide wire passed<br>4/26 (15.4%)
McGrath-Cadell et al 2016 (n=40)	27 (67.5%)	2 (5.0%)	12 (30.0%)	-	-	-	-	-	-
Adams et al 2018 (n=22)	17 (77.3%)	2 (9.0%)	3 (13.6%)	IVUS or OCT<br>3 (13.6%)		-	-	-	-
Yuvaraj et al 2020 (n=11)	-	-	-	-	-	-	-	-	-
Fahey et al 2021 (n=85)	-	-	Stents<br>12 (14.1%)	-	-	-	-	-	-
Murphy et al 2022 (n=30)	-	-	-	-	-	-	-	-	-
Murphy et al 2023 (n=35)	-	-	-	-	-	-	-	-	-
Murphy et al 2024 (n=310)	256 (82.6%)	6 (1.9%)	42 (13.5%)	-	-	-	-	-	-
Dang et al 2023 (n=172)	-	4 (2.3%)	16 (9.3%)	-	-	-	-	-	-
Tarr et al 2022 (n=91)	-	-	-	-	-	-	-	-	-

WHO region<br>Study	Medical therapy	CABG	PCI	IVUS	OCT	Aspiration	Conventional balloon	Cutting balloon	Wiring
Tarr et al 2024 (n=200)	-	-	-	-	-	-	-	-	-
McAlister et al 2021 (n=113)	99 (88.0%)	-	14 (12.0%)	7 (6.0%)	4 (4.0%)	-	-	-	-
Wong et al 2022 (n=32)	30 (93.8%)	1 (3.1%)	1 (3.1%)	-	-	-	-	-	-
Uribe et al 2015 (n=8)	-	0	8 (100%)<br>BMS: 3 (37.0%)<br>DES: 5 (63.0%)	-	-	-	-	-	-
Meng et al 2017 (n=21)	18 (86.0%)	-	3 (14.0%)	All cases had IVUS or OCT		-	-	-	-
Sun et al 2019 (n=85)	-	-	47 (55.3%)<br>Successful	√	√	-	-	-	-
Liu et al 2019 (n=118)	33 (28.0%)	18 (15.2%)	67 (56.8%)	-	-	-	-	-	-
Hui et al 2020 (n=70)	56 (80.0%)	2 (2.9%)	12 (17.1%)	-	-	-	-	-	-
Chang et al 2022 (n=30)	-	-	7 (23.3%)<br><br>Stents<br>5 (16.7%)	20 (66.7%)	-	-	2 (6.7%)	1 (3.3%)	-
Ma et al 2023 (n=81)	36 (44.4%)	5 (6.2%)	40 (49.4%)	-	-	-	-	-	-
Tokura et al 2014 (n=10)	1 (10.0%)	0	Stents<br>4 (40.0%)	7 (70.0%)	-	3 (30.0%)	2 (20.0%)	-	-
Nakashima et al 2016 (n=63)	28 (44.0%)	1 (2.0%)	34 (54.0%)<br><br>Stents<br>23/34 (68.0%)	30/32 (93.7%)	2/32 (6.3%)	-	POBA<br>11/34 (32.0%)	-	-
Nishiguchi et al 2016 (n=13)	-	-	-	-	13 (100%)	-	-	-	-
Nishiguchi et al 2017 (n=12)	-	-	12 (100%)	-	12 (100%)	8 (67.0%)	-	-	Repositioning<br>2 (17.0%)
Kubota et al 2020 (n=7)	7 (100%)	-	-	-	-	-	-	-	-
Inohara et al 2020 (n=322)	137 (42.6%)	10 (3.1%)	175 (54.3%)<br><br>Stents	205 (63.7%)	-	-	-	-	-

WHO region<br>Study	Medical therapy	CABG	PCI	IVUS	OCT	Aspiration	Conventional balloon	Cutting balloon	Wiring
			108 (33.5%)						
Inoue et al 2021<br>(n=19)	-	-	Stents<br>7 (36.8%)	14 (73.7%)	2 (10.5%)	2 (10.5%)	6 (31.5%)	2 (10.5%)	2 (10.5%)
Taguchi et al 2023<br>(n=16)	13 (81.25%)	-	3 (18.75%)	6 (37.5%)	1 (6.25%)	-	-	-	-
Kim et al 2021<br>(n=13)	5 (38.5%)	0	Stents<br>6 (46.1%)	4 (30.8%)	0	-	POBA<br>2 (15.4%)<br>One failed	-	-
Turkey									
Celik et al 2001<br>(n=9)	1 (11.1%)	7 (77.8%)	Stents<br>1 (11.1%)	-	-	-	-	-	-
Unal et al 2008<br>(n=6)	0	6 (100%)	1 (16.7%)<br>Failed	-	-	-	-	-	-
Canga et al 2018<br>(n=22)	8 (36.4%)	7 (31.8%)	7 (31.8%)	-	-	-	-	-	-
Özkan et al 2023<br>(n=49)	8 (16.3%)	0	41 (83.7%)	-	-	-	-	-	-

Abbreviations: BMS, bare metal stent; CABG, coronary artery bypass grafting; CAG, coronary angiography; DES, drug-eluting stent; IVUS, intravascular ultrasound; OCT, optical coherence tomography; PCI, percutaneous coronary intervention.

Table S16. Percutaneous procedure characteristics

WHO region<br>Study	Stent number	Stent size/diameter (mm)	Stent length (mm)	Femoral access	Radial access	Dye volume (mL)	Fluoroscopy time (min)	IABP use	Impella use	ECMO use
Eastern Mediterranean region										
Benahmed et al 2018 (n=7)	-	-	-	-	-	-	-	-	-	-
Daoulah et al 2021 (n=83)	-	-	-	-	-	-	-	-	-	-
Almasi et al 2022 (n=15)	-	-	-	14 (93.3)	-	-	-	-	-	-
South-East Asia region										
Sharma et al 2016 (n=5)	-	-	-	-	-	-	-	-	-	-
Valappil et al 2018 (n=16)	-	3.17±0.34	41.6± 14.5	-	-	189.4±69.3	10.4±6.9	-	-	-
Western Pacific Region										
Rashid et al 2016 (n=21)	-	-	-	-	-	-	-	-	-	-
McGrath-Cadell et al 2016 (n=40)	-	-	-	-	-	-	-	-	-	-
Adams et al 2018 (n=22)	-	-	-	-	-	-	-	-	-	-
Yuvaraj et al 2020 (n=11)	-	-	-	-	-	-	-	-	-	-
Fahey et al 2021 (n=85)	-	-	-	31 (36.5%)	54 (63.5%)	-	-	-	-	-
Murphy et al 2022 (n=30)	-	-	-	-	-	-	-	-	-	-
Murphy et al 2023 (n=35)	-	-	-	-	-	-	-	-	-	-
Murphy et al 2024 (n=310)	-	-	-	-	-	-	-	-	-	-
Dang et al 2023 (n=172)	-	-	-	-	-	-	-	-	-	-
Tarr et al 2022 (n=91)	-	-	-	-	-	-		-	-	-

WHO region<br>Study	Stent number	Stent size/diameter (mm)	Stent length (mm)	Femoral access	Radial access	Dye volume (mL)	Fluoroscopy time (min)	IABP use	Impella use	ECMO use
Tarr et al 2024 (n=200)	-	-	-	-	-	-	-	-	-	-
McAlister et al 2021 (n=113)	-	-	-	-	-	-	-	-	-	-
Wong et al 2022 (n=32)	-	-	-	-	-	-	-	-	-	-
Uribe et al 2015 (n=8)	-	-	-	-	-	-	-	-	-	-
Meng et al 2017 (n=21)	-	-	-	-	-	-	-	-	-	-
Sun et al 2019 (n=85)	-	-	-	-	-	-	-	-	-	-
Liu et al 2019 (n=118)	-	-	-	-	-	-	-	-	-	-
Hui et al 2020 (n=70)	-	-	-	-	-	-	-	-	-	-
Chang et al 2022 (n=30)	-	-	-	-	-	-	-	-	-	-
Ma et al 2023 (n=81)	1.89±1.11	-	-	-	-	-	-	-	-	-
Tokura et al 2014 (n=10)	-	-	-	-	-	-	-	-	-	-
Nakashima et al 2016 (n=63)	-	1.03±0.16	-	-	-	-	-	-	-	-
Nishiguchi et al 2016 (n=13)	-	-	-	-	-	-	-	-	-	-
Nishiguchi et al 2017 (n=12)	1.42±0.90	3.17±0.50	29.1±19.1	-	-	-	-	-	-	-
Kubota et al 2020 (n=7)	-	-	-	-	-	-	-	-	-	-
Inohara et al 2020 (n=322)	-	-	-	-	-	-	-	31 (9.6%)	-	-
Inoue et al 2021 (n=19)	-	-	-	-	-	-	-	2 (10.5%)	-	1 (5.3%)
Taguchi et al 2023 (n=16)	-	-	-	-	-	-	-	-	-	-

WHO region<br>Study	Stent number	Stent size/diameter (mm)	Stent length (mm)	Femoral access	Radial access	Dye volume (mL)	Fluoroscopy time (min)	IABP use	Impella use	ECMO use
Kim et al 2021 (n=13)	-	-	-	-	-	-	-	-	-	-
Turkey										
Celik et al 2001 (n=9)	-	-	-	-	-	-	-	-	-	-
Unal et al 2008 (n=6)	-	-	-			-	-	-	-	-
Canga et al 2018 (n=22)	-	-	-	-	-	-	-	-	-	-
Özkan et al 2023 (n=49)	-	-	-			-	-	-	-	-

Abbreviations: ECMO, extracorporeal membrane oxygenation; IABP, intra-aortic balloon pump; IVUS, intravascular ultrasound; min, minutes; OCT, optical coherence tomography.

Table S17. Angiographic characteristics of SCAD lesion(s)

WHO region<br>Study	1 lesion	2 lesions	3 lesions or multiple	LM	LAD	Diagonal	LCx	OM	RCA	RCA branch	Ramus	Stenosis severity (%)	Lesion length (mm)	Length <20 mm & ≥20 mm
Eastern Mediterranean region														
Benahmed et al 2018 (n=7)	7 (100%)	-	-	-	2 (28.6%)	-	-	-	5 (71.4%)	-	-	-	-	-
Daoulah et al 2021 (n=83)	-	-	Multi 8 (9.6%)	10 (12.0%)	36 (43.4%)	13 (15.7%)	8 (9.6%)	4 (4.8%)	18 (21.7%)	PDA 2 (2.4%)	-	Max 80.0 (50.0 - 95.0)	Max 25.0 (18.0 - 36.0)	-
Almasi et al 2022 (n=15)	-	-	-	1 (6.7%)	12 (80.0%)	Included with LAD	1 (6.7%)	-	1 (6.7%)	-	-	-	-	-
South-East Asia region														
Sharma et al 2016 (n=5)	5 (100%)	-	-	-	3 (60.0%)	-	-	-	1 (20.0%)	-	-	-	-	-
Valappil et al 2018 (n=16)	14 (87.5%)	2 (12.5%)	-	-	7 (38.9%)	-	2 (12.5%)	2 (12.5%)	7 (38.9%)	-	-	-	-	-
Western Pacific Region														
Rashid et al 2016 (n=21)	-	-	-	-	12/26 (46.1%)	-	4/26 (15.4%)	-	10/26 (38.5%)	-	-	-	-	
McGrath-Cadell et al 2016 (n=40)	35 (87.5%)	4 (10.0%)	1 (2.5%)	1 (2.5%)	26 (65.0%)	1 (2.5%)	5 (12.5%)	5 (12.5%)	3 (7.5%)	4 (10.0%)	1 (2.5%)	-	-	-
Adams et al 2018 (n=22)	-	-	Multi 3 (13.6%)	2 (8.0%)	11 (48.0%)	-	3 (15.0%)	-	6 (29.0%)	-	-	-	-	-
Yuvaraj et al 2020 (n=11)	-	-	-	-	-	-	-	-	-	-	-	-	-	-
Fahey et al 2021 (n=85)	-	-	Multi 4 (4.7%)	-	40 (47.0%)	-	16 (18.8%)	-	25 (29.4%)	-	-	-	-	-
Murphy et al 2022 (n=30)	-	-	-	-	-	-	-	-	-	-	-	-	-	-
Murphy et al 2023 (n=35)	-	-	-	-	-	-	-	-	-	-	-	-	-	-
Murphy et al 2024 (n=310)	-	-	-	-	-	-	-	-	-	-	-	-	-	-

WHO region Study	1 lesion	2 lesions	3 lesions or multiple	LM	LAD	Diagonal	LCx	OM	RCA	RCA branch	Ramus	Stenosis severity (%)	Lesion length (mm)	Length <20 mm & ≥20 mm
Dang et al 2023 (n=172)	-	-	-	-	-	-	-	-	-	-	-	-	-	-
Tarr et al 2022 (n=91)	-	-	-	-	-	-	-	-	-	-	-	-	-	-
Tarr et al 2024 (n=200)	-	-	-	-	-	-	-	-	-	-	-	-	-	-
McAlister et al 2021 (n=113)	-	-	Multi 7 (6.0%)	2 (2.0)	51 (45.0%)	-	25 (22.0%)	-	28 (25.0%)	-	-	-	-	-
Wong et al 2022 (n=32)	-	-	4 (12.5%)	1 (2.6%)	12 (31.6%)	-	13 (34.2%)	-	10 (26.3%)	-	2 (5.3%)	-	-	-
Uribe et al 2015 (n=8)	-	-	-	0	0	0	3 (37.0%)	0	5 (63.0%)	0	0	-	-	-
Meng et al 2017 (n=21)	20 (95.0%)	-	-	-	14 (67.0%)	-	3 (14.0%)	-	4 (19.0%)	-	-	76.9± 20.6	36.6±8.6	-
Sun et al 2019 (n=85)	-	>1 lesion 3 (3.5%)		2/92 (2.2%)	48/92 (52.2%)	-	5/92 (5.4%)	-	37/92 (40.2%)	-	-	-	-	-
Liu et al 2019 (n=118)	-	-	Multi 3 (2.5%)	7 (5.7%)	30 (24.6%)	-	6 (4.9%)	-	79 (64.8%)	-	-	-	-	<20 mm: 93 (76.2%) ≥20 mm: 29 (23.8%)
Hui et al 2020 (n=70)	-	>1 lesion 12 (17.1%)		1 (1.4%)	35 (50.0%)	-	25 (35.7%)	-	20 (28.6%)	-	-	-	-	-
Chang et al 2022 (n=30) SCIH	-	5 (16.7%)	-	-	20 (66.7%)	-	7 (23.3%)	-	8 (26.7%)	-	-	-	-	-
Ma et al 2023 (n=81)	-	-	-	2 (2.5%)	26 (32.1%)	-	8 (9.9%)	-	45 (55.6%)	-	-	68.5± 24.2	40.1± 24.3	-
Tokura et al 2014 (n=10)	9 (90.0%)	1 (10.0%)	-	-	4 (40.0%)	-	1 (10.0%)	-	6 (60.0%)	-	-	-	-	-
Nakashima et al 2016 (n=63)	-	-	Multi 7 (11.0%)	-	37 (59.0%)	-	4 (6.0%)	-	15 (24.0%)	-	-	-	-	-
Nishiguchi et al 2016 (n=13)	-	-	-	-	8 (62.0%)	-	-	-	5 (38.0%)	-	-	73.8±9.0	12.9±3.8	-

WHO region<br>Study	1 lesion	2 lesions	3 lesions or multiple	LM	LAD	Diagonal	LCx	OM	RCA	RCA branch	Ramus	Stenosis severity (%)	Lesion length (mm)	Length <20 mm & ≥20 mm
Nishiguchi et al 2017 (n=12)	-	-	-	-	5 (42.0%)	-	-	-	7 (58.0%)	-	-	74.2± 10.4	34.3± 26.9	-
Kubota et al 2020 (n=7)	-	-	-	-	-	1 (14.0%)	3 (43.0%)	-	3 (43.0%)	-	-	-	-	-
Inohara et al 2020 (n=322)	-	-	-	-	-	-	-	-	-	-	-	-	-	-
Inoue et al 2021 (n=19)	18 (94.7%)	1 (5.3%) LAD + RCA	-	-	13 (68.4%)	-	2 (10.5%)	-	5 (26.3%)	-	-	-	-	-
Taguchi et al 2023 (n=16)	-	-	-	-	Branch 5 (31.25%)	Branch 2 (12.5%)	2 (12.5%)	Branch 2 (12.5%)	Peripher al 4 (25.0%)	-	-	-	-	-
Kim et al 2021 (n=13)	13 (100%)	-	-	-	10 (76.9%)	-	1 (7.7%)	-	2 (15.4%)	-	-	-	-	-
Turkey														
Celik et al 2001 (n=9)	4 (44.4%)	2 (22.2%)	3 (33.3%)	2 (22.2%)	5 (55.5%)	-	-	-	2 (22.2%)	-	-	-	-	-
Unal et al 2008 (n=6)	6 (100%)	-	-	5 (83.3%)	-	-	-	-	1 (16.7%)	-	-	-	-	-
Canga et al 2018 (n=22)	-	-	Multi 3 (13.6%)	3 (13.6%)	9 (40.9%)	-	2 (9.1%)	-	13 (59.1%)	-	-	-	26.5± 18.7	-
Özkan et al 2023 (n=49)	-	-	-	-	21 (42.9%)	-	19 (38.8%)	-	9 (18.4%)	-	-	-	-	-

Abbreviations: LAD, left anterior descending artery; LCx, left circumflex artery; OM, Obtuse marginal; LM, left main artery; PDA, Posterior descending artery; RCA, right coronary artery; SCIH, spontaneous coronary intramural hematoma.

“Continued Part 1” Table S17. Angiographic characteristics of SCAD lesion(s)

WHO region<br>Study	Ostial	Proximal	Middle	Distal	Diffuse	CTO	No tortuosity (%)	Tortuosity >90% & <90%	Thrombus	Isolated SCAD	AS & SCAD	Iatrogenic dissection
Eastern Mediterranean region												
Benahmed et al 2018 (n=7)	-	-	-	-	-	-	-	-	-	-	-	-
Daoulah et al 2021 (n=83)	-	-	-	-	-	-	-	-	-	-	-	-
Almasi et al 2022 (n=15)	-	-	-	-	-	-	-	-	-	-	-	-
South-East Asia region												
Sharma et al 2016 (n=5)	-	4 (80.0%)	3 (60.0%)	1 (20.0%)	-	-	-	-	-	-	-	-
Valappil et al 2018 (n=16)	-	5 (27.7%)	2 (11.1%)	3 (16.6%)	11 (61.1%)	1 (5.6%)	6 (37.5%)	>90%: 7 (43.8%) <90%: 3 (18.7%)	-	8 (50.0%)	8 (50.0%)	-
Western Pacific Region												
Rashid et al 2016 (n=21)	-	-	-	-	-	-	-	-	Double-lumen hematoma 5/26 (19.2%)	-	Luminal ± occlusion 12/26 (46.2%)	-
McGrath-Cadell et al 2016 (n=40)	-	-	-	-	-	-	-	-	-	-	-	-
Adams et al 2018 (n=22)	-	-	-	-	-	-	-	-	-	-	-	-
Yuvaraj et al 2020 (n=11)	-	-	-	-	-	-	-	-	-	-	-	-
Fahey et al 2021 (n=85)	-	-	-	-	-	-	-	-	-	-	-	4 (4.7%)
Murphy et al 2022 (n=30)	-	-	-	-	-	-	-	-	-	-	-	-
Murphy et al 2023 (n=35)	-	-	-	-	-	-	-	-	-	-	-	-
Murphy et al 2024 (n=310)	-	-	-	-	-	-	-	-	-	-	-	-

WHO region<br>Study	Ostial	Proximal	Middle	Distal	Diffuse	CTO	No tortuosity (%)	Tortuosity >90% & <90%	Thrombus	Isolated SCAD	AS & SCAD	Iatrogenic dissection
Dang et al 2023 (n=172)	-	-	-	-	-	-	-	-	-	-	-	-
Tarr et al 2022 (n=91)	-	-	-	-	-	-	-	-	-	-	-	-
Tarr et al 2024 (n=200)	-	-	-	-	-	-	-	-	-	-	-	-
McAlister et al 2021 (n=113)	-	-	-	-	-	-	-	-	-	-	-	-
Wong et al 2022 (n=32)	-	5 (13.1%)	6 (15.8%)	Diffuse or branch<br>27 (71.1%)	-	-	-	-	-	-	-	-
Uribe et al 2015 (n=8)	-	-	-	-	-	-	-	-	-	-	1 (12.0%)	-
Meng et al 2017 (n=21)	-	-	-	-	-	-	-	-	IMH<br>20 (95.0%)<br><br>Intimal tearing<br>1 (5.0%)	-	-	-
Sun et al 2019 (n=85)	-	-	-	-	-	-	-	-	-	-	-	-
Liu et al 2019 (n=118)	0	58 (47.5%)	37 (30.3%)	27 (22.2%)	-	-	-	-	-	-	-	-
Hui et al 2020 (n=70)	-	-	Mid & distal<br>65 (92.9%)		-	-	-	-	-	-	-	-
Chang et al 2022 (n=30)<br>SCIH	-	-	-	-	-	-	-	-	-	-	-	-
Ma et al 2023 (n=81)	-	38 (46.9%)	30 (37.0%)	13 (16.1%)	-	-	-	-	6 (7.4%)	-	35 (43.2%)	-
Tokura et al 2014 (n=10)	-	-	-	-	-	-	-	-	-	-	-	-
Nakashima et al 2016 (n=63)	-	-	-	-	-	-	-	-	-	-	-	-
Nishiguchi et al 2016 (n=13)	-	-	-	-	-	-	-	-	-	-	-	-

WHO region<br>Study	Ostial	Proximal	Middle	Distal	Diffuse	CTO	No tortuosity (%)	Tortuosity >90% & <90%	Thrombus	Isolated SCAD	AS & SCAD	Iatrogenic dissection
Nishiguchi et al 2017 (n=12)	-	-	-	-	-	-	-	-	-	-	9 (75.0%)	-
Kubota et al 2020 (n=7)	-	-	-	6 (86.0%)	-	-	-	-	-	-	-	-
Inohara et al 2020 (n=322)	-	-	-	-	-	-	-	-	-	-	-	-
Inoue et al 2021 (n=19)	0	2 (10.5%)	8 (42.1%)	9 (47.4%)	-	-	-	-	-	-	-	-
Taguchi et al 2023 (n=16)	-	2 (12.5%)	-	9 (56.2%)	√	-	-	-	-	-	-	-
Kim et al 2021 (n=13)	0	Proximal-distal<br>1 (7.7%)	-	12 (92.3%)	-	-	-	-	-	-	-	-
Turkey												
Celik et al 2001 (n=9)	-	-	-	-	-	-	-	-	-	-	9 (100%)	-
Unal et al 2008 (n=6)	-	-	-	-	-	-	-	-	-	-	-	-
Canga et al 2018 (n=22)	-	-	-	-	-	-	-	-	-	-	-	
Özkan et al 2023 (n=49)	-	-	-	-	-	-	-	-	-	-	-	-

Abbreviations: AS, atherosclerosis; CTO, chronic total occlusion; IMH, intramural hematoma; SCAD, spontaneous coronary artery dissection; TIMI, thrombolysis in myocardial infarction.

“Continued Part 2” Table S17. Angiographic characteristics of SCAD lesion(s)

WHO region<br>Study	Initial TIMI flow<br>0 & I	Initial TIMI flow<br>II & III	OCT findings			SCAD Type or Saw Classification					NHLBI Classification	
			TCEF	Thrombus	Ca deposit	Type 1	Type 2A	Type 2B	Type 3	Multiple type	A/B/C	D/E/F
Eastern Mediterranean region												
Benahmed et al 2018 (n=7)	-	-	-	-	-	-	-	-	-	-	-	-
Daoulah et al 2021 (n=83)	0:<br>8 (9.6%)<br><br>I:<br>13 (15.7%)	II:<br>21 (25.3%)<br><br>III:<br>41 (49.4%)	-	-	-	43 (51.8%)	2A & 2B<br>35 (42.2%)		3 (3.6%)	2 (2.4%)	-	-
Almasi et al 2022 (n=15)*	<III: 4/11 (36.4%)	III: 7/11 (63.6%)	-	-	-	2 (13.3%)	6 (40.0%)	2 (13.3%)	2 (13.3%)	Type 4<br>3 (20.0%)	-	-
South-East Asia region												
Sharma et al 2016 (n=5)	-	-	-	-	-	4 (80.0%)	2A & 2B<br>1 (20.0%)		0	0	-	-
Valappil et al 2018 (n=16)	-	III:<br>13 (72.2%)	-	-	-	-	-	-	-	-	-	-
Western Pacific Region												
Rashid et al 2016 (n=21)	-	-	-	-	-	-	-	-	-	-	-	-
McGrath-Cadell et al 2016 (n=40)	-	-	-	-	-	-	-	-	-	-	-	-
Adams et al 2018 (n=22)	-	-	-	-	-	-	-	-	-	-	-	-
Yuvaraj et al 2020 (n=11)	-	-	-	-	-	-	-	-	-	-	-	-
Fahey et al 2021 (n=85)	-	-	-	-	-	-	-	-	-	-	-	-
Murphy et al 2022 (n=30)	-	-	-	-	-	-	-	-	-	-	-	-
Murphy et al 2023 (n=35)	-	-	-	-	-	-	-	-	-	-	-	-
Murphy et al 2024 (n=310)	-	-	-	-	-	-	-	-	-	-	-	-
Dang et al 2023 (n=172)	-	-	-	-	-	-	-	-	-	-	-	-

WHO region<br>Study	Initial TIMI flow<br>0 & I	Initial TIMI flow<br>II & III	OCT findings			SCAD Type or Saw Classification					NHLBI Classification	
			TCEF	Thrombus	Ca deposit	Type 1	Type 2A	Type 2B	Type 3	Multiple type	A/B/C	D/E/F
Tarr et al 2022 (n=91)	-	-	-	-	-	-	-	-	-	-	-	-
Tarr et al 2024 (n=200)	-	-	-	-	-	-	-	-	-	-	-	-
McAlister et al 2021 (n=113)	-	-	-	-	-	15 (13.0%)	43 (38.0%)	48 (42.0%)	7 (6.0%)	-	-	-
Wong et al 2022 (n=32)	-	-	-	-	-	7/38 (18.4%)	2A & 2B 30/38 (78.9%)		1/38 (2.6%)	-	-	-
Uribe et al 2015 (n=8)	-	-	-	-	-	-	-	-	-	-	-	E: 7 (88.0%)<br>F: 1 (12.0%)
Meng et al 2017 (n=21)	-	-	-	-	-	1 (5.0%)	2A & 2B 14 (67.0%)		6 (28.0%)			
Sun et al 2019 (n=85)	-	-	-	-	-	-	-	-	-	-	-	-
Liu et al 2019 (n=118)	0-II:<br>29 (23.8%)	III:<br>93 (76.2%)	-	-	-	-	-	-	-	-	58 (47.5%)	64 (52.5%)
Hui et al 2020 (n=70)	-	-	-	-	-	19 (27.1%)	2A & 2B 49 (70.0%)		2 (2.7%)	-	-	-
Chang et al 2022 (n=30) SCIH#	-	-	-	-	-	IA:<br>7 (23.3%)<br>IB:<br>7 (23.3%)<br>IA&IB:<br>7 (23.3)#	2A & 2B<br>4 (13.3%)#	-	5 (16.7%)#	-	-	-
Ma et al 2023 (n=81)	0:<br>9 (11.1)<br><br>I:<br>4 (4.9)	II:<br>2 (2.5%)<br><br>III:<br>66 (81.5%)	-	-	-	47 (58.0)	2A & 2B 30 (37.1%)		4 (4.9%)	-	-	-
Tokura et al 2014 (n=10)	-	-	-	-	-	-	-	-	-	-	-	-
Nakashima et al 2016 (n=63)	0:<br>27 (44.0%)<br><br>I:	II:<br>16 (26.0%)<br><br>III:	-	-	-	27 (43.0%)	2A & 2B 35 (55.0%)		1 (2.0%)	-	-	-

WHO region<br>Study	Initial TIMI flow<br>0 & I	Initial TIMI flow<br>II & III	OCT findings			SCAD Type or Saw Classification					NHLBI Classification	
			TCEF	Thrombus	Ca deposit	Type 1	Type 2A	Type 2B	Type 3	Multiple type	A/B/C	D/E/F
	7 (12.0%)	12 (19.0%)										
Nishiguchi et al 2016 (n=13)	0:<br>4 (31.0%)<br><br>I:<br>1 (8.0%)	II:<br>1 (8.0%)<br><br>III:<br>7 (54.0%)	0	9 (69.0%)	6 (46.0%)	-	-	-	-	-	-	-
Nishiguchi et al 2017 (n=12)	0: 6 (50.0%)<br>I: 1 (8.0%)	II:<br>3 (25.0%)<br><br>III:<br>2 (17.0%)	-	-	-	-	-	-	-	-	-	-
Kubota et al 2020 (n=7)	-	III:<br>7 (100%)	-	-	-	-	-	-	-	-	-	-
Inohara et al 2020 (n=322)	-	-	-	-	-	-	-	-	-	-	-	-
Inoue et al 2021 (n=19)	-	-	-	-	-	11 (57.9%)	2A & 2B<br>5 (26.3%)		2 (10.5%)	Unknown:<br>1 (5.3%)	-	-
Taguchi et al 2023 (n=16)	-	-	-	-	-	2 (12.5%)	2A & 2B<br>8 (50.0%)		6 (37.5%)	-	-	-
Kim et al 2021 (n=13)**	0:<br>5 (38.5%)<br><br>I:<br>1 (7.7.%)	II:<br>4 (30.8%)<br><br>III: 3 (23.1%)	-	-	-	1 (7.7%)	4 (30.8%)	5 (38.5%)	3 (23.1%)	-	-	-
Turkey												
Celik et al 2001 (n=9)	-	-	-	-	-	-	-	-	-	-	-	-
Unal et al 2008 (n=6)	-	-	-	-	-	-	-	-	-	-	-	-
Canga et al 2018 (n=22)	-	-	-	-	-	-	-	-	-	-	-	-
Özkan et al 2023 (n=49)	-	-	-	-	-	10 (20.4%)	2A & 2B<br>32 (65.3%)		7 (14.3%)	-	-	-

*Final TIMI flow grade: <III:2/11 (18.2%); III: 9/11 (81.8%)

**Final TIMI flow grade: 0: 0; I: 1 (7.7%); II: 2 (15.4%); III: 10 (%76.9).

Abbreviations: Ca, calcium; NHLBI, National, Heart, Lung, and Blood Institute; OCT, optical coherence tomography; SCAD, spontaneous coronary artery dissection; TCFA, thin-cap fibroatheroma; TIMI, thrombolysis in myocardial infarction.

Table S18. Medications at discharge

WHO region<br>Study	SAPT	DAPT	Aspirin	Clopidogrel	Prasugrel	Ticagrelor	Ticlopidine	Cilostazol	OAC	BB	ACEI/ARB	CCB	Diuretics	Statin
Eastern Mediterranean region														
Benahmed et al 2018 (n=7)	-	√	-	-	-	-	-	-	-	√	√	-	-	√
Daoulah et al 2021 (n=83)	-	-	82 (98.8%)	P_2Y_{12} inhibitor 75 (90.4%)				-	-	74 (89.2%)	54 (65.1%)	9 (10.8%)	-	70 (84.3%)
Almasi et al 2022 (n=14)	-	-	13 (92.2%)	P_2Y_{12} inhibitor 12 (85.7%)				-	1 (7.1%)	10 (71.4%)	ACEI: 8 (57.1%) ARB: 9 (64.3%)	3 (21.4%) Nitrate: 6 (42.9%)	0	12 (85.7%)
South-East Asia region														
Sharma et al 2016 (n=5)	-	-	-	-	-	-	-	-	-	-	-	-	-	-
Valappil et al 2018 (n=16)	-	-	-	-	-	-	-	-	-	-	-	-	-	-
Western Pacific Region														
Rashid et al 2016 (n=21)	-	-	21 (100%)	8 (38.1%)	4 (19.0%)	4 (19.0%)	-	-	-	16 (76.2%)	17 (80.9%)	CCB or Nitrate 5 (23.8%)	-	18 (85.7%)
McGrath-Cadell et al 2016 (n=40)	20/27 (74.0%) in medically treated	-	24/27 (89.0%) in medically treated	-	-	-	-	-	-	21/27 (78.0%) in medically treated	16/27 (59.0%) in medically treated	2/27 (7.0%) in medically treated	-	11/27 (41.0%) in medically treated
Adams et al 2018 (n=22)	-	18 (81.8%)	22 (100%)	13 (59.1%)	-	5 (22.7%)	-	-	2 (9.1%)	16 (72.7%)	16 (72.7%)	-	-	15 (68.2%)
Yuvaraj et al 2020 (n=11)	-	-	-	-	-	-	-	-	-	-	-	-	-	-
Fahey et al 2021 (n=85)	-	-	-	-	-	-	-	-	-	-	-	-	-	-
Murphy et al 2022 (n=30)	-	-	-	-	-	-	-	-	-	-	-	-	-	-
Murphy et al 2023 (n=35)	-	-	-	-	-	-	-	-	-	-	-	-	-	-

WHO region Study	SAPT	DAPT	Aspirin	Clopidogrel	Prasugrel	Ticagrelor	Ticlopidine	Cilostazol	OAC	BB	ACEI/ARB	CCB	Diuretics	Statin
Murphy et al 2024 (n=310)	-	-	-	-	-	-	-	-	-	-	-	-	-	-
Dang et al 2023 (n=172)	-	100 (58.1%)	143 (83.1%)	-	-	-	-	-	-	136 (79.1%)	5 (2.9%)	-	-	93 (54.1%)
Tarr et al 2022 (n=91)	-	-	-	-	-	-	-	-	-	-	-	-	-	-
Tarr et al 2024 (n=200)	-	-	-	-	-	-	-	-	-	-	-	-	-	-
McAlister et al 2021 (n=113)	20 (18.0%)	91 (81.0%)	-	-	-	-	-	-	7 (6.0%)	82 (73.0%)	48 (42.0%)	11 (10.0%)	-	77 (68.0%)
Wong et al 2022 (n=32)	-	-	-	-	-	-	-	-	-	-	-	-	-	-
Uribe et al 2015 (n=8)	-	-	-	-	-	-	-	-	-	-	-	-	-	-
Meng et al 2017 (n=21)	-	-	-	-	-	-	-	-	-	-	-	-	-	-
Sun et al 2019 (n=85)	-	-	79 (92.9%)	P_2Y_{12} inhibitor 76 (89.4%)				-	-	58 (68.2%)	57 (67.1%)	-	-	78 (91.8%)
Liu et al 2019 (n=118)	-	110 (93.2%)	-	-	-	-	-	-	90 (76.3%)	-	-	-	-	-
Hui et al 2020 (n=70)	-	-	-	-	-	-	-	-	-	-	-	-	-	-
Chang et al 2022 (n=30) SCIH	8 (26.7%)	19 (63.3%)	-	-	-	-	-	-	-	20 (66.7%)	23 (76.7%)	10 (33.3%)	-	25 (83.3%)
Ma et al 2023 (n=81)	-	-	-	-	-	-	-	-	-	-	-	-	-	-
Tokura et al 2014 (n=10)	-	-	-	-	-	-	-	-	-	-	-	-	-	-
Nakashima et al 2016 (n=63)	-	-	-	-	-	-	-	-	-	-	-	-	-	-
Nishiguchi et al 2016 (n=13)	-	-	-	-	-	-	-	-	-	-	-	-	-	-
Nishiguchi et al 2017 (n=12)	-	-	12 (100%)	11 (92.0%) including ticlopidine	-	-	-	-	-	4 (33.0%)	9 (75.0%)	4 (33.0%)	0	8 (67.0%)

WHO region<br>Study	SAPT	DAPT	Aspirin	Clopidog<br>rel	Prasugre<br>l	Ticagrelo<br>r	Ticlopidi<br>ne	Cilostazo<br>l	OAC	BB	ACEI/AR<br>B	CCB	Diuretics	Statin
Kubota et al 2020<br>(n=7)	-	-	-	-	-	-	-	-	-	-	-	-	-	-
Inohara et al 2020<br>(n=322)	-	-	287<br>(89.1%)	117<br>(36.3%)	127<br>(39.4%)	0	2 (0.6%)	6 (1.9%)	-	200<br>(62.1%)	159<br>(49.4%)	-	-	161<br>(50.0%)
Inoue et al 2021<br>(n=19)	9<br>(47.4%)	9<br>(47.4%)	-	-	-	-	-	-	-	-	-	-	-	-
Taguchi et al 2023<br>(n=16)	-	-	-	-	-	-	-	-	-	-	-	-	-	-
Kim et al 2021<br>(n=13)	-	6<br>(46.2%)	7<br>(53.8%)	7<br>(53.8%)	0	3<br>(23.1%)	-	-	-	9<br>(69.2%)	10<br>(76.9%)	4<br>(30.8%)	-	9<br>(69.2%)
Turkey														
Celik et al 2001<br>(n=9)	-	-	-	-	-	-	-	-	-	-	-	-	-	-
Unal et al 2008<br>(n=6)	-	-	-	-	-	-	-	-	-	-	-	-	-	-
Canga et al 2018<br>(n=22)	-	-	-	-	-	-	-	-	-	-	-	-	-	-
Özkan et al 2023<br>(n=49)	-	-	-	-	-	-	-	-	-	-	-	-	-	-

Abbreviations: ACEI, angiotensin-converting enzyme inhibitors; ARB, angiotensin receptor blockers; BB, beta-blockers; CCB, calcium channel blockers; DAPT, dual antiplatelet therapy; OAC, oral anticoagulants; SAPT, single antiplatelet therapy.

Table S19. Post-procedure or in-hospital outcomes

WHO region<br>Study	PCI-related complications	Repeat CAG	Cardiac arrest/VA	CS	ICD	Extension of dissection	In-hospital MACE	In-hospital death
Eastern Mediterranean region								
Benahmed et al 2018 (n=7)	-	-	-	-	-	-	-	-
Daoulah et al 2021 (n=83)	-	-	Recurrent VA 5 (6%)	4 (4.8%)	1 (1.2%)	3 (3.6%)	Composite events 8 (9.6%)	1 (1.2%)
Almasi et al 2022 (n=15)	-	-	-	-	-	-	-	1 (6.7%)
South-East Asia region								
Sharma et al 2016 (n=5)	-	-	-	-	-	-	-	-
Valappil et al 2018 (n=16)	0	-	-	-	-	-	0	0
Western Pacific Region								
Rashid et al 2016 (n=21)	-	-	-	-	-	-	-	0
McGrath-Cadell et al 2016 (n=40)	-	-	-	-	-	-	-	-
Adams et al 2018 (n=22)	-	-	-	-	-	-	-	-
Yuvaraj et al 2020 (n=11)	-	-	-	-	-	-	-	-
Fahey et al 2021 (n=85)	-	-	-	-	-	-	-	-
Murphy et al 2022 (n=30)	-	-	-	-	-	-	-	-
Murphy et al 2023 (n=35)	-	-	-	-	-	-	-	-
Murphy et al 2024 (n=310)	-	-	-	-	3 (1.0%)			
Dang et al 2023 (n=172)	-	-	-	-	-	-	-	-
Tarr et al 2022 (n=91)	-	-	-	-	-	-	-	-
Tarr et al 2024 (n=200)	-	-	-	-	-	-	-	-

WHO region<br>Study	PCI-related complications	Repeat CAG	Cardiac arrest/VA	CS	ICD	Extension of dissection	In-hospital MACE	In-hospital death
McAlister et al 2021 (n=113)	-	20 (18.0%)	-	-	-	-	-	-
Wong et al 2022 (n=32)	-	-	-	-	-	-	-	-
Uribe et al 2015 (n=8)	-	-	-	-	-	-	-	0
Meng et al 2017 (n=21)	-	-	-	-	-	-	-	-
Sun et al 2019 (n=85)	-	-	-	-	-	-	-	-
Liu et al 2019 (n=118)	-	0	-	-	-	-	-	1 (0.85%)
Hui et al 2020 (n=70)	-	-	-	-	-	-	-	-
Chang et al 2022 (n=30)<br>SCIH	-	-	-	-	-	-	-	-
Ma et al 2023 (n=81)	-	-	-	-	-	-	-	-
Tokura et al 2014 (n=10)	Day 6: Stent thrombosis<br>1 (10.0%)	-	-	-	-	-	-	-
Nakashima et al 2016 (n=63)	Dissection during PCI<br>1 (1.6%)	-	-	-	-	Propagation of dissection flap<br>8/34 (24.0%)	-	-
Nishiguchi et al 2016 (n=13)	-	-	-	-	-	-	-	-
Nishiguchi et al 2017 (n=12)	1 (8.0%)	-	-	-	-	-	0	0
Kubota et al 2020 (n=7)	0	-	-	-	-	-	-	0
Inohara et al 2020 (n=322)	-	-	-	-	-	-	-	8 (2.5%)
Inoue et al 2021 (n=19)	-	-	-	-	-	-	-	-
Taguchi et al 2023 (n=16)	-	-	-	Shock<br>2 (12.5%)	-	-	-	0

WHO region<br>Study	PCI-related complications	Repeat CAG	Cardiac arrest/VA	CS	ICD	Extension of dissection	In-hospital MACE	In-hospital death
Kim et al 2021 (n=13)	-	-	-	-	-	-	-	-
Turkey								
Celik et al 2001 (n=9)	-	-	-	-	-	-	-	-
Unal et al 2008 (n=6)	1 (16.7%) Failed	-	-	-	-	-	-	1 (16.7%) – due to sepsis on the 30th day post procedure.
Canga et al 2018 (n=22)	-	-	-	-	-	-	1 (4.5%)	0
Özkan et al 2023 (n=49)	-	-	-	-	-	-	-	-

Abbreviations: CS, cardiogenic shock; ICD, Implantable cardioverter-defibrillator; MACE, major cardiovascular adverse effects; VA, ventricular arrhythmia.

Table S20. Clinical outcomes at follow-up

WHO region<br>Study	Follow-up period	Death	MI	Composite events	Repeat CAG	Revascularization	SCAD status	SCAD events	Others
Eastern Mediterranean region									
Benahmed et al 2018 (n=7)	16.4 (1.0 – 29.0) months	0	0	0	-	-	-	-	-
Daoulah et al 2021 (n=83)	18.8 (9.0-40.0) months	1 (1.2%)	6 (7.3%)	6 (7.3%)				De novo<br>4 (4.9%)	SSMAD<br>1 (1.2%)
Almasi et al 2022 (n=15)	1 year	0	1 (6.7%)	2 (13.3%)	0	0	-	-	-
South-East Asia region									
Sharma et al 2016 (n=5)	7.0 (2.0 – 19.0) months	0	1 (20.0%)	-	-	-	-	-	-
Valappil et al 2018 (n=16)	8 months to 3 years	0	0	0	0	0	-	-	-
Western Pacific Region									
Rashid et al 2016 (n=21)	-	-	-	-	-	-	-	-	-
McGrath-Cadell et al 2016 (n=40)	16.0 (8.0 - 28.5) months	0	-	Stent thrombosis:<br>1 (2.5%)	-	-	-	Recurrent<br>3 (7.5%)	Coronary artery aneurysm<br>2 (5.0%)
Adams et al 2018 (n=22)	1 year	1 (4.5%)	-	MACCE<br>3 (13.6%)	2 (9.1%)	-	-	0	Stroke<br>0<br><br>Readmission<br>2 (9.1%)<br><br>LOS – hospital<br>5.0 (3.0 – 9.0) days
Yuvaraj et al 2020 (n=11)	-	-	-	-	-	-	-	-	-
Fahey et al 2021 (n=85)	-	-	-	-				-	-
Murphy et al 2022 (n=30)	-	-	-	-	-	-	-	-	-

WHO region<br>Study	Follow-up period	Death	MI	Composite events	Repeat CAG	Revascularization	SCAD status	SCAD events	Others
Murphy et al 2023 (n=35)	-	-	-	-	-	-	-	-	-
Murphy et al 2024 (n=310)	-	-	-	-	-	-	-	-	-
Dang et al 2023 (n=172)									
Tarr et al 2022 (n=91)	-	-	-	-	-	-	-	-	-
Tarr et al 2024 (n=200)	-	-	-	-	-	-	-	-	-
McAlister et al 2021 (n=113)	30 days	1 (1.0%)	5 (4.0%)	MACE<br>10 (9.0%)	At median 109 days<br>20 (18.0%)	3 (3.0%)	-	-	Stroke<br>3 (3.0%)<br><br>Cardiac arrest<br>1 (1.0%)
Wong et al 2022 (n=32)	40.5 (20.8 - 107) days	-	-	-	-	-	Unhealed<br>25/38 (65.8%)	-	-
Uribe et al 2015 (n=8)*	1 year	-	-	-	-	-	-	-	-
Meng et al 2017 (n=21)	-	-	-	-	-	-	-	-	-
Sun et al 2019 (n=85)	-	-	-	-	-	-	-	-	-
Liu et al 2019 (n=118)	43.0 (25.0 – 75.0) months	8 (6.8%)	32 (27.4%)	-		9 (7.7%)<br>PCI (n=7)<br>CABG (n=2)		-	-
Hui et al 2020 (n=70)	28.3 months	CV<br>2 (2.9%)	MI & SCAD<br>5 (7.1%)	MACE<br>11 (15.7%)	-	TVR<br>4 (5.7%)	-	Recurrent<br>3 (4.3%)	-
Chang et al 2022 (n=30)<br>SCIH	29.3±13.5 months	-	-	-	-	-	-	-	Stent restenosis<br>1 (3.3)<br><br>Hospitalized<br>8 (26.7%)
Ma et al 2023 (n=81)	1 year	Cardiac<br>1 (1.2%)	UA<br>6 (7.4%)	MACE<br>10 (12.3%)	57 (70.4%)	-	Healed<br>40/57 (70.2%)<br><br>Residual	-	-

WHO region<br>Study	Follow-up period	Death	MI	Composite events	Repeat CAG	Revascularization	SCAD status	SCAD events	Others
							17/57 (29.8%)		
Tokura et al 2014 (n=10)	7.3 months	0	-	-	-	-	-	-	-
Nakashima et al 2016 (n=63)	34.0 (3.0 – 160) months	-	18 (28.6%) at 1 year	MACE<br>23 (37.0%) at 5 years	-	-	-	Propagation<br>4 (6.0%)<br><br>Recurrent<br>14 (22.0%)**	SCD<br>1 (1.6%) at 1 year
Young females (n=45)<br>From Nakashima study above it	50.0 (1.0 – 197) months	Cardiac<br>1 (2.0%)	16 (36.0%)	MACE<br>17 (38.0%)<br><br>MACE<br>20 (44.0%) at 5 years	-	Urgent:<br>0	-	Propagation<br>4 (8.9%)<br><br>Recurrent<br>12 (26.7%)	-
Nishiguchi et al 2016 (n=13)	-	-	-	-	-	-	-	-	-
Nishiguchi et al 2017 (n=12)	17.1±13.3 months	0	-	-	-	-	-	1 (8.0%) at 6 months	-
Kubota et al 2020 (n=7)	-	-	-	-	-	-	-	-	-
Inohara et al 2020 (n=322)	-	-	-	-				-	-
Inoue et al 2021 (n=19)	960 (686 – 1504) days	-	-	-				De novo<br>2 (10.5%) at Days 4 & 5 of discharge	-
Taguchi et al 2023 (n=16)	-	-	-	-	-	-	-	-	-
Kim et al 2021 (n=13)	935 (121 – 4125) days	-	-	-		PCI<br>1 (7.7%)<br>At 236 days		-	-
Turkey									
Celik et al 2001 (n=9)	1 year	0	0	0	As follow-up CAG<br>1 (11.1%)	0	-	0	-
Unal et al 2008 (n=6)	-	-	-	-	-	-	-	-	-

WHO region Study	Follow-up period	Death	MI	Composite events	Repeat CAG	Revascularization	SCAD status	SCAD events	Others
Canga et al 2018 (n=22)	-								
Özkan et al 2023 (n=49)	-	-	-	-	-	-	-	-	-

**4 cases were lost to follow up; the other 4 cases had no events at 1-year follow-up.*

***7 patients experienced recurrent SCAD during the first 30 days after the primary SCAD events. Median interval from the onset of SCAD to the second episode of SCAD was 42 (1 – 2968) days.*

Abbreviations: CAG, coronary angiography; CV, cardiovascular; MACCE, major adverse cardiovascular or cerebrovascular event; MI, myocardial infarction; MACE, major adverse cardiovascular event; MI, myocardial infarction; SCD, sudden cardiac death; SSMAD, spontaneous superior mesenteric artery dissection; TVR, target vessel revascularization; UA, unstable angina.

Table S21. Sensitivity analyses according to study size and confirmed SCAD diagnosis

21.1.Prevalence/incidence

Variable	Pooled variable	Sensitivity analysis (n > 50)	No. of studies	Sensitivity analysis based on confirmed diagnosis	No. of studies
Prevalence	1.0% (95% CI: 0.0; 3.0); I^2 = 99%	0.44% (95% CI: 0.06; 3.1); I^2 = 100%	5	1.2% (95% CI: 0.45; 3.2); I^2 = 99%	17
Prevalence without studies that enrolled only females	1.0% (95% CI: 0.0; 2.0); I^2 = 99%	0.44% (95% CI: 0.03; 5.4); I^2 = 100%	4	Same	Same
Prevalence in studies that enrolled only females	5.0% (95% CI: 1.0; 16.0); I^2 = 99%	-	1	7.5% (95% CI: 2.4; 21.3); I^2 = 98%	5

21.2. Baseline characteristics

Variable	Pooled variable	Sensitivity analysis according to study size (n > 50)	No. of studies	Sensitivity analysis based on confirmed diagnosis	No. of studies
Age	Mean 51.3 years (95% CI: 48.79; 53.82); I^2 = 94%	Mean 51.9 years (95% CI: 48.2; 55.6); I^2 = 94%	7	51.3 years (95% CI: 48.5; 54.1); I^2 = 94%	22
Females	54.0% (95% CI: 38.0; 70.0); I^2 = 89%	54.0% (95% CI: 23.6; 81.7); I^2 = 96%	6	Same	Same
Dyslipidemia	31.0% (95% CI: 26.0; 37.0); I^2 = 58%	35.4% (95% CI: 30.4; 40.8); I^2 = 53%	7	30.0% (95% CI: 24.0; 36.6); I^2 = 58%	19
Hypertension	43.0% (95% CI: 36.0; 51.0); I^2 = 74%	49.6% (95% CI: 40.4; 58.8); I^2 = 82%	7	42.0% (95% CI: 34.4; 50.0); I^2 = 70%	22
Diabetes	19.0% (95% CI: 13.0; 27.0); I^2 = 80%	17.5% (95% CI: 8.4; 32.9]; I^2 = 93%	6	22.4% (95% CI: 16.8; 29.1); I^2 = 58%	18
Smokers	30.0% (95% CI: 23.0; 39.0); I^2 = 79%	37.9% (95% CI: 24.8; 53.0); I^2 = 91%	6	Same	Same
Prior myocardial infarction	11.0% (95% CI: 5.0; 24.0); I^2 = 90%	12.1% (95% CI: 4.3; 29.8); I^2 = 95%	4	13.5 (95% CI: 5.8; 28.6); I^2 = 80%	7

FMD	22.0% (95% CI: 7.0; 50.0); I^2 = 88%	-	1	Same	Same
Physical stress	17.0% (95% CI: 7.0; 34.0); I^2 = 66%	10.3% (95% CI: 6.3; 16.4); I^2 = 0%	2	Same	Same
Emotional stress	29.0% (95% CI: 16.0; 46.0); I^2 = 66%	34.6% (95% CI: 24.5; 46.2); I^2 = 49%	2	Same	Same
LVEF	Mean 51.4% (95% CI: 47.44; 55.36); I^2 = 97%	Mean 52.3% (95% CI: 46.71; 57.85); I^2 = 98%	4	Same	Same

21.3. SCAD pooled variable – Presentation

Variable	Pooled variable	Sensitivity analysis (n > 50)	No. of studies	Sensitivity analysis based on confirmed diagnosis	No. of studies
STEMI	50.0% (95% CI: 38.0; 62.0); I^2 = 79%	50.3% (95% CI: 22,7; 77.7); I^2 = 92%	5	Same	Same
NSTEMI	38.0% (95% CI: 25.0; 52.0); I^2 = 86%	21.7% (95% CI: 10.2; 40.2); I^2 = 91%	4	Same	Same
Unstable angina	18.0% (95% CI: 8.0; 33.0); I^2 = 87%	22.0% (95% CI: 3.7; 67.0); I^2 = 95%	3	Same	Same

21.4. SCAD pooled variable – In-hospital management

Variable	Pooled variable	Sensitivity analysis (n > 50)	No. of studies	Sensitivity analysis based on confirmed diagnosis	No. of studies
Medical treatment	48.0% (95% CI: 32.0; 65.0); I^2 = 85%	47.0% (95% CI: 29.7; 65.1); I^2 = 91%	5	46.1% (95% CI: 28.8; 64.4); I^2 = 87%	12
CABG	14.0% (95% CI: 4.0; 37.0); I^2 = 88%	5.6% (95% CI: 2.9; 10.5); I^2 = 78%	6	17.0% (95% CI: 4.7; 45.7; I^2 = 85%	8
PCI	42.0% (95% CI: 31.0; 53.0); I^2 = 83%	42.6% (95% CI: 31.7; 54.3); I^2 = 87%	7	42.2% (95% CI: 30.9; 54.4); I^2 = 81%	20
IVUS	64.0% (95% CI: 46.0; 79.0); I^2 = 69%	82.0% (95% CI: 35.9; 97.4); I^2 = 88%	2	65.0% (95% CI: 41.8; 82.6; I^2 = 74%	6
OCT	53.0% (95% CI: 4.0; 97.0); I^2 = 88%	-	1	Same	Same

POBA	19.0% (95% CI: 11.0; 33.0); I^2 = 43%	-	1	Same	Same

21.5. Initial TIMI flow (SCAD lesion)

Variable	Pooled variable	Sensitivity analysis (n > 50)	No. of studies	Sensitivity analysis based on confirmed diagnosis	No. of studies
TIMI flow grade 0 or I	33.0% (95% CI: 23.0; 46.0); I^2 = 78%	30.0% (95% CI: 13.0; 55.0); I^2 = 92%	3	Same	Same
TIMI flow grade II or III	67.0% (95% CI: 55.0; 77.0); I^2 = 79%	72.2% (95% CI: 53.8; 85.2); I^2 = 90%	4	Same	Same

21.6. Stenosis severity (SCAD lesion)

Variable	Pooled variable	Sensitivity analysis (n > 50)	No. of studies	Sensitivity analysis based on confirmed diagnosis	No. of studies
Stenosis severity	73.98% (95% CI: 70.98; 76.98); I^2 = 42%	72.73% (95% CI: 65.12; 80.35); I^2 = 85%	2	Same	Same
Lesion length	Mean 28.98 mm (95% CI: 20.58; 37.38); I^2 = 98%	Mean 32.80 mm (95% CI: 18.99; 46.61); I^2 = 96%	2	Same	Same

21.7. Number of SCAD lesions

Variable	Pooled variable	Sensitivity analysis (n > 50)	No. of studies	Sensitivity analysis based on confirmed diagnosis	No. of studies
One lesion	89.0% (95% CI: 76.0; 95.0); I^2 = 41%	-	0	Same	Same
Two lesions	12.0% (95% CI: 7.0; 20.0); I^2 = 32%	8.5% (95% CI: 1.7; 33.7); I^2 = 85%	2	Same	Same
Three or more lesions	10.0% (95% CI: 5.0; 21.0); I^2 = 64%	7.2 (95% CI: 3.2; 15.5); I^2 = 63%	3	Same	Same

21.8. Affected coronary artery

Variable	Pooled variable	Sensitivity analysis (n > 50)	No. of studies	Sensitivity analysis based on confirmed diagnosis	No. of studies
Left main artery	9.0% (95% CI: 4.0; 20.0); I^2 = 72%	4.6% (95% CI: 2.1; 9.8); I^2 = 63%	5	Same	Same
LAD artery	50.0% (95% CI: 43.0; 57.0); I^2 = 67%	46.2% (95% CI: 35.3; 57.4); I^2 = 83%	7	Same	Same
LCX artery	13.0% (95% CI: 9.0; 19.0); I^2 = 66%	7.8 (95% CI: 5.4; 11.3); I^2 = 0%	4	Same	Same
RCA	32.0% (95% CI: 24.0; 42.0); I^2 = 82%	31.6% (95% CI: 15.0; 54.7); I^2 = 95%	6	Same	Same
Branches	28.0% (95% CI: 19.0; 38.0); I^2 = 59%	31.4% (95% CI: 20.6; 44.6); I^2 = 77%	3	Same	Same

21.9. SCAD type

Variable	Pooled variable	Sensitivity analysis (n > 50)	No. of studies	Sensitivity analysis based on confirmed diagnosis	No. of studies
Type 1 SCAD	35.0% (95% CI: 22.0; 51.0); I^2 = 81%	45.0% (95% CI: 32.0; 58.5); I^2 = 81%	4	Same	Same
Type 2 SCAD	49.0% (95% CI: 38.0; 60.0); I^2 = 75%	51.2% (95% CI: 36.5; 65.6); I^2 = 84%	4	Same	Same
Type 3 SCAD	11.0% (95% CI: 6.0; 19.0); I^2 = 70%	4.0% (95% CI: 2.0; 7.0); I^2 = 0%	4	Same	Same

21.10. Affected segment of coronary artery

Variable	Pooled variable	Sensitivity analysis (n > 50)	No. of studies	Sensitivity analysis based on confirmed diagnosis	No. of studies
Proximal segment	31.0% (95% CI: 16.0; 51.0); I^2 = 71%	48.2% (95% CI: 41.4; 55.2); I^2 = 0%	2	Same	Same
Middle segment	34.0% (95% CI: 28.0; 40.0); I^2 = 27%	34.0% (95% CI: 27.5; 40.6); I^2 = 0%	2	Same	Same
Distal segment	41.0% (95% CI: 21.0; 65.0); I^2 = 79%	20.0% (95% CI: 14.1 27.6); I^2 = 28%	2	Same	Same

21.11. SCAD pooled variable – Medications

Variable	Pooled variable	Sensitivity analysis (n > 50)	No. of studies	Sensitivity analysis based on confirmed diagnosis	No. of studies
Aspirin	91.0% (95% CI: 77.0; 97.0); I^2 = 74%	93.4 (95% CI: 84.4; 97.3); I^2 = 66%	3	91.8% (95% CI: 72.0; 98.0; I^2 = 80%	5
SAPT	36.0% (95% CI: 19.0; 58.0); I^2 = 54%	-	0	Same	Same
DAPT	68.0% (95% CI: 37.0; 89.0); I^2 = 90%	-	1	Same	Same
P_2Y_{12} inhibitors	84.0% (95% CI: 74.0; 91.0); I^2 = 80%	84.3% (95% CI: 67.7; 93.2); I^2 = 91%	3	88.6% (95% CI: 83.4; 92.3); I^2 = 0%	5
Beta-blockers	69.0% (95% CI: 55.0; 79.0); I^2 = 75%	74.6% (95% CI: 53.4; 88.3); I^2 = 90%	3	70.0% (95% CI: 53.6; 82.1; I^2 = 74%	6
RAAS inhibitors	67.0% (95% CI: 56.0; 76.0); I^2 = 77%	60.0% (95% CI: 47.9; 70.8); I^2 = 84%	3	69.0% (95% CI: 62.6; 74.7); I^2 = 6%	6
Calcium channel blockers	24.0% (95% CI: 14.0; 37.0); I^2 = 57%	-	1	Same	Same
Statins	78.0% (95% CI: 64.0; 87.0); I^2 = 91%	79.0% (95% CI: 47.8; 94.0); I^2 = 96%	3	83.2% (95% CI: 74.8; 89.2); I^2 = 39%	6

21.12. In-hospital outcomes

Variable	Pooled variable	Sensitivity analysis (n > 50)	No. of studies	Sensitivity analysis based on confirmed diagnosis	No. of studies
PCI-related complications (e.g., iatrogenic dissection, stent thrombosis)	7.0% (95% CI: 2.0; 18.0); I^2 = 9%	-	1	Same	Same
Extension of dissection	7.0% (95% CI: 2.0; 23.0); I^2 = 73%	Same	Same	Same	Same
In-hospital CV events (any)	9.0% (95% CI: 5.0; 16.0); I^2 = 0%	-	1	Same	Same

In-hospital death (any cause)	3.0% (95% CI: 2.0; 5.0); I^2 = 35%	2.1% (95% CI: 1.1; 3.8); I^2 = 0%	3	3.2% [0.79; 12.4; I^2 = 50%	4

21.13. Outcomes at follow-up

Variable	Pooled variable	Sensitivity analysis (n > 50)	No. of studies	Sensitivity analysis based on confirmed diagnosis	No. of studies
Follow-up duration	Mean 23.42 months (95% CI: 15.7; 31.1); I^2 = 100%	Mean 30.19 (95% CI: 12.4; 47.9); I^2 = 100%	5	Same	Same
All-cause death	3.0% (95% CI: 1.0; 7.0); I^2 = 37%	Same	Same	Same	Same
Myocardial infarction	13.0% (95% CI: 7.0; 23.0); I^2 = 79%	13.6% (95% CI: 6.7; 25.8); I^2 = 85%	5	Same	Same
Composite CV events	16.0% (95% CI: 8.0; 28.0); I^2 = 81%	16.1% (95% CI: 7.7; 30.6); I^2 = 86%	4	Same	Same
Any SCAD event (de novo, recurrent, propagation)	10.0% (95% CI: 4.0; 22.0); I^2 = 81%	9.4% (95% CI: 2.4; 30.7); I^2 = 90%	3	Same	Same

Table S22. Patient characteristics and outcomes in international registries and nationwide studies

Variable (%) or mean/median	Prospective follow-up	Prospective study	Registries & Health System			
	Saw et al 2017 [1]	Saw et al 2019 & 2022 [2,3] same references 8 & 46 in manuscript	Mortensen et al 2009 [4]	García-Guimarães et al 2021 & 2022 [5,6]	Wilander et al 2022 [7]	Sharma et al 2019 & Clare et al 2019 [8,9]
Study characteristics						
Country	Canada	North America	Denmark	Spain	Sweden	USA
Number of sites	VGH	Canada: 20<br>USA: 2	3 Danish counties	34	30	MGH [8]<br>KPSC Health system [9]
Enrolment period	April 2012 to December 2016	June 2014 to June 2018	January 1999 to December 2007	June 2015 to April 2019	December 2015 to December 2017	January 2006 to December 2016 [9]<br>July 2013 to October 2017 [8]
No. of SCAD patients	327	750	CAG report: 22	CAG report: 389 (441 lesions)	CAG report: 147	CAG report: 113 & 208
SCAD incidence/prevalence	-	-	22/11175 (2.0%)	318/216897 (0.15%) [6]	147/32601 (0.45%)	208/26390 (0.78%) [9]
Patient characteristics						
Age (year) mean/median	53.0	52.0	49.0	53.0	53.0	47.0 & 49.0
Female sex	90.8	88.5	81.8	88.0	75.5	87.0 & 88.9
Co-morbidities & precipitating factors						
Dyslipidemia	25.7	20.3	-	33.0	13.7	14.0 & 27.9
Hypertension	36.4	32.1	43.0	36.0	26.5	27.0 & 30.8
Diabetes mellitus	4.6	4.7	0	5.0	2.0	3.0 & 8.2
Smoker	9.8	11.6	73.0 (active & inactive)	26.0	38.1 (active & inactive)	22.0 [8]
Prior MI	0.9	8.4	0	-	10.9	6.7 [9]
FMD	62.7	32.9	-	25.0	-	-
Anxiety	13.5	19.7	-	18.0	-	27.0 [8]
Depression	22.6	19.5		20.0		18.0 & 13.9
Physical stress	28.1	38.7	-	12.0	-	-
Emotional stress	48.3	50.3	-	26.0		-
Presentation & Management						
STEMI	25.7	29.7	72.7	40.0	47.6	43.0 & 19.7
NSTEMI	74.3	69.9	18.2	54.0	-	57.0 [8]

Variable (%) or mean/median	Prospective follow-up	Prospective study	Registries & Health System			
	Saw et al 2017 [1]	Saw et al 2019 & 2022 [2,3] same references 8 & 46 in manuscript	Mortensen et al 2009 [4]	García-Guimarães et al 2021 & 2022 [5,6]	Wilander et al 2022 [7]	Sharma et al 2019 & Clare et al 2019 [8,9]
Conservative	83.2	84.3	31.8	78.0	59.9	66.0 & 84.6
PCI	16.5	14.1	59.1	22.0	40.1	32.0 & 11.1
IVUS/OCT	-	2.1/5.5*	36.0	10.0 – 14.0	24.5	-
Angiographic characteristics						
LAD	45.2#	52.1	86.4	44.0	-	70.0 & 42.2
LCX	31.8#	37.7	0	32.0	-	7.0 & 6.4
RCA	23.0#	23.2	14.0	21.0	-	13.0 & 12.3
Type 1	25.6#	29.0**	-	19.0	12.2	34.0 [8]
Type 2	69.8#	60.2**	-	61.4	72.8	75.0 [8]
Type 3	4.7#	10.8**	-	9.0	4.1	1.0 [8]
Medications upon discharge						
Aspirin	92.0 (n=288)	93.7	-	93.0	93.1	-
P_2Y_{12} inhibitors	62.2 (n=288)	67.4	-	60.0	85.4	70.2 [9]
Beta-blockers	83.0 (n=288)	84.8	-	79.0	81.9	83.2 [9]
ACEI/ARB	57.6 (n=288)	57.4	-	51.0	59.2	57.2 [9]
CCB	16.7 (n=288)	10.4	-	8.0	-	18.8 [9]
Statin	54.2 (n=288)	55.1	76.0	79.0	76.4	80.8 [9]
Clinical outcomes						
Follow-up period (months)	37	1 & 36	35.2	29.0 (17.0 - 38.0)	17.3 (longest)	1,3 & 12 [9]
Death	1.2	1-m: 0.1<br>36-m: 0.8	4.5	2.5	2.7	1-m: 1.4<br>3-m: 1.4<br>12-m: 2.4
Myocardial infarction	16.8	1-m: 6.1<br>36-m: 9.9	9.1	7.6	2.0	-
Composite of events	19.9	1-m: 8.8<br>36-m: 14.0	18.2	13.0	10.9	-
Recurrent SCAD	10.4	1-m: 1.2***<br>36-m: 5.6***	4.5	2.0		-
Healed SCAD lesion	-	-	-	91.0	-	-

#Of 387 dissections
*For IVUS/OCT confirmed SCAD.
**Of 1002 dissections.
***De novo or extension of previous dissections.
Abbreviations: ACEI, angiotensin-converting enzyme inhibitors; ARB, angiotensin receptor blockers; Cath, catheterization laboratory; CCB, calcium channel blockers; D1, diagonal branch 1; D2, diagonal branch 2; FMD, fibromuscular dysplasia disease; IMH, intramural hematoma; IQR, interquartile range; IVUS, intravascular ultrasound; KPSC, Kaiser Permanente Southern California; LAD, left anterior descending artery;

Variable (%) or mean/median	Prospective follow-up	Prospective study	Registries & Health System			
	Saw et al 2017 [1]	Saw et al 2019 & 2022 [2,3] same references 8 & 46 in manuscript	Mortensen et al 2009 [4]	García-Guimarães et al 2021 & 2022 [5,6]	Wilander et al 2022 [7]	Sharma et al 2019 & Clare et al 2019 [8,9]

LCx, Left circumflex; MGH, Massachusetts General Hospital; MI, myocardial infarction; NIS, Nationwide Inpatient Sample; NRD, Nationwide Readmissions Database; NSTEMI, non- ST segment elevation myocardial infarction; OCT, optical coherence tomography; OM1, obtuse marginal 1 artery; PDA, posterior descending artery; PCI, percutaneous coronary intervention; RCA, right coronary artery; STEMI, ST segment elevation myocardial infarction; SCAD, spontaneous coronary artery dissection; VGH, Vancouver General Hospital; WHO, The World Health Organization.

References

1. Saw J, Humphries K, Aymong E, et al. Spontaneous Coronary Artery Dissection: Clinical Outcomes and Risk of Recurrence. *J Am Coll Cardiol*. 2017;70(9):1148-1158.
2. Saw J, Starovoytov A, Humphries K, et al. Canadian spontaneous coronary artery dissection cohort study: in-hospital and 30-day outcomes. *Eur Heart J*. 2019;40(15):1188-1197.
3. Saw J, Starovoytov A, Aymong E, et al. Canadian Spontaneous Coronary Artery Dissection Cohort Study: 3-Year Outcomes. *J Am Coll Cardiol*. 2022;80(17):1585-1597.
4. Mortensen KH, Thuesen L, Kristensen IB, Christiansen EH. Spontaneous coronary artery dissection: a Western Denmark Heart Registry study. *Catheter Cardiovasc Interv*. 2009;74(5):710-717.
5. García-Guimaraes M, Bastante T, Macaya F, et al. Spontaneous coronary artery dissection in Spain: clinical and angiographic characteristics, management, and in-hospital events. *Rev Esp Cardiol (Engl Ed)*. 2021;74(1):15-23.
6. Garcia-Guimaraes M, Masotti M, Sanz-Ruiz R, et al. Clinical outcomes in spontaneous coronary artery dissection. *Heart*. 2022;108(19):1530-1538. Published 2022 Sep 12.
7. Wilander H, Pagonis C, Venetsanos D, et al. Nationwide observational study of incidence, management and outcome of spontaneous coronary artery dissection: a report from the Swedish Coronary Angiography and Angioplasty register. *BMJ Open*. 2022;12(6):e060949. Published 2022 Jun 1.
8. Sharma S, Kaadan MI, Duran JM, et al. Risk Factors, Imaging Findings, and Sex Differences in Spontaneous Coronary Artery Dissection. *Am J Cardiol*. 2019;123(11):1783-1787.
9. Clare R, Duan L, Phan D, et al. Characteristics and Clinical Outcomes of Patients With Spontaneous Coronary Artery Dissection. *J Am Heart Assoc*. 2019;8(10):e012570.

Supplementary figures

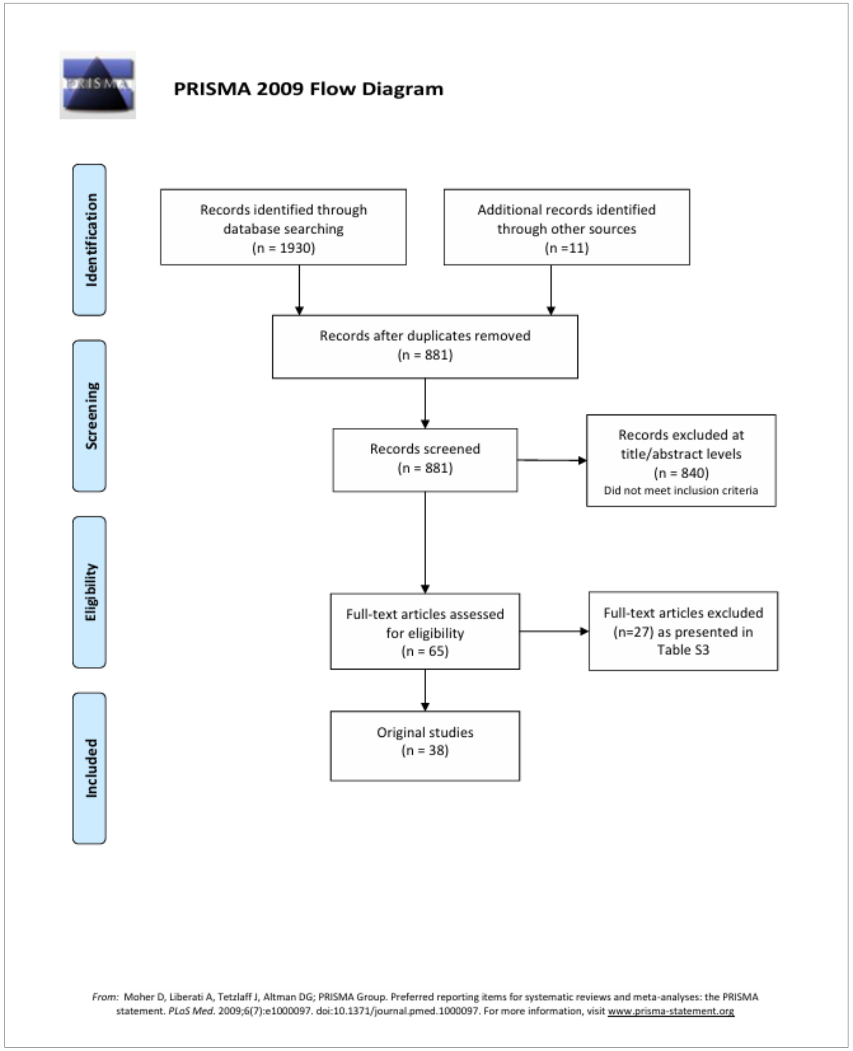

Figure S1. PRISMA flow diagram

Forest plots (single proportion meta-analysis)

SCAD pooled variable – Characteristics

Prevalence

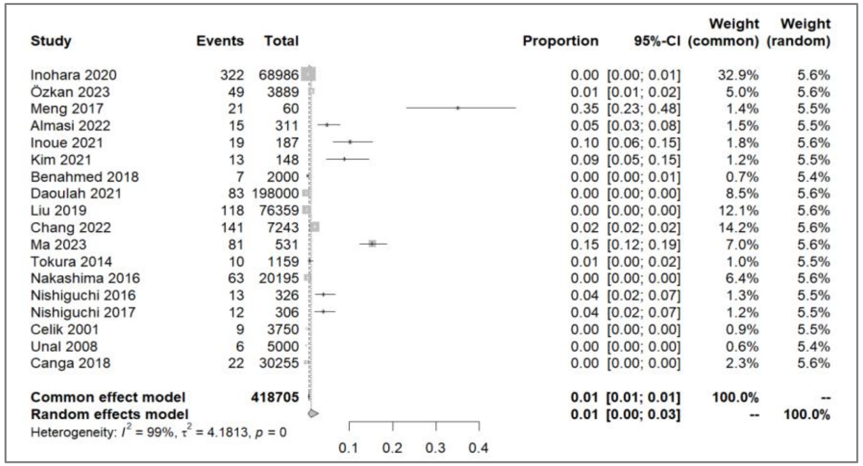

Figure S2. Pooled proportions of prevalence for all studies that reported it

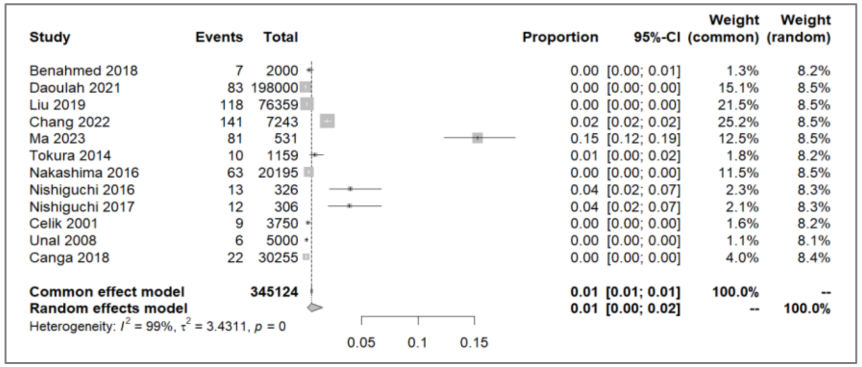

Figure S3. Pooled proportions of prevalence without studies recruited only females

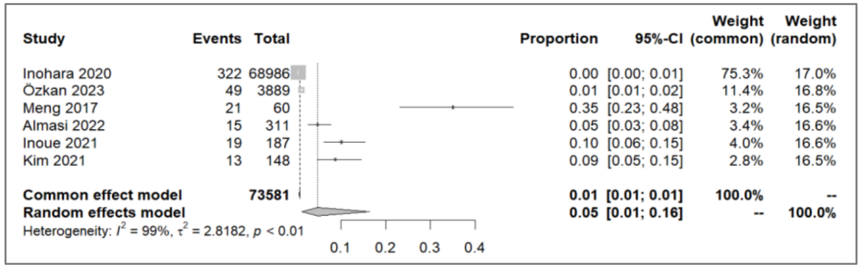

Figure S4. Pooled proportions of prevalence for studies that recruited only females

Baseline characteristics

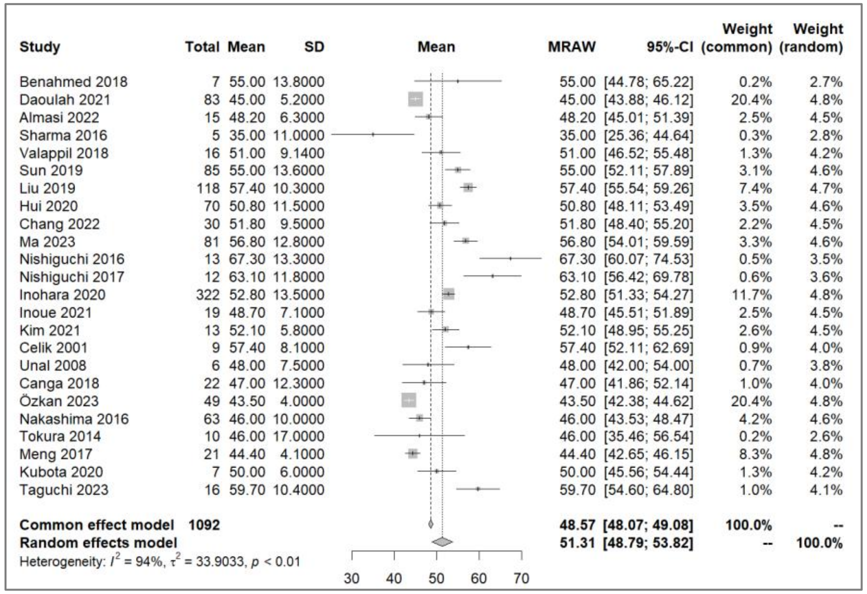

Figure S5. Pooled means of age

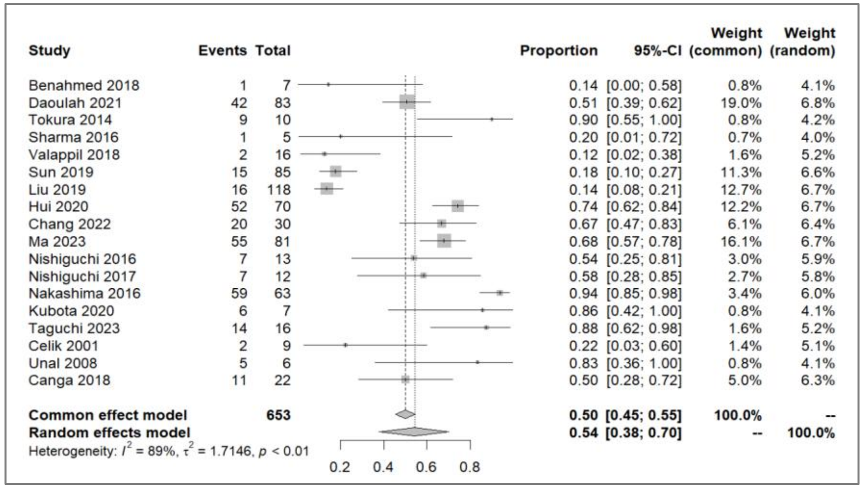

Figure S6. Pooled proportions of female variable (excluding studies that recruited only females)

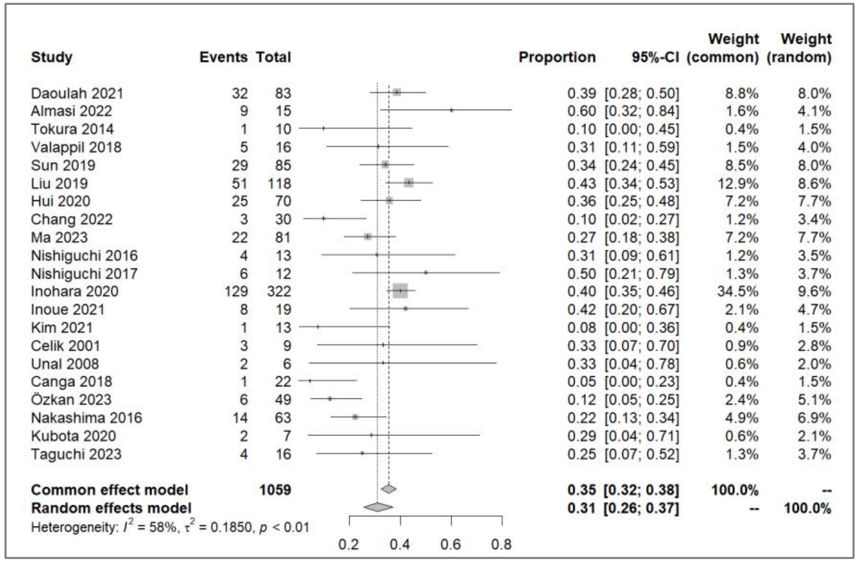

Figure S7. Pooled proportions of dyslipidemia

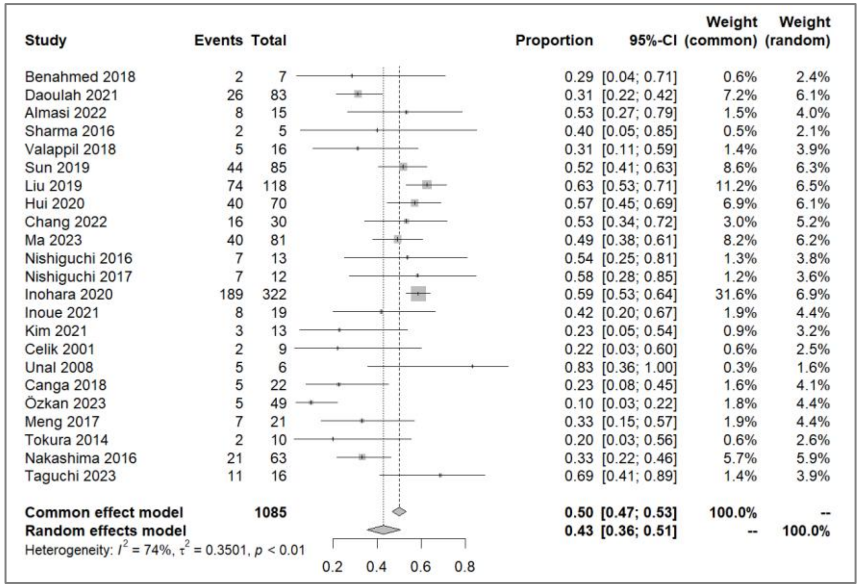

Figure S8. Pooled proportions of hypertension

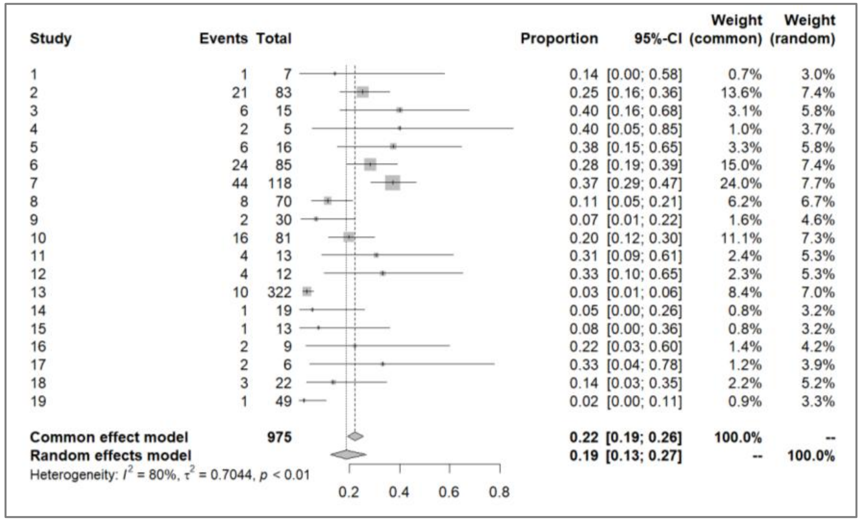

Figure S9. Pooled proportions of diabetes

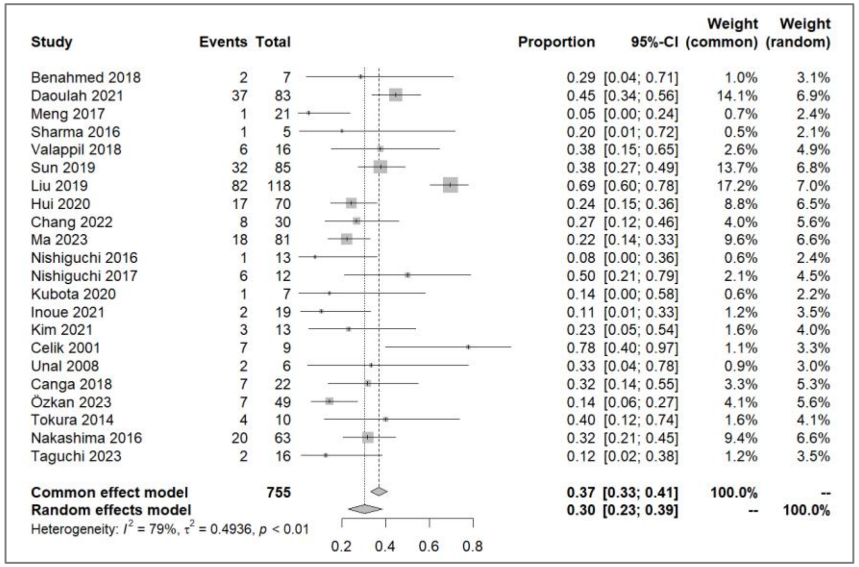

Figure S10. Pooled proportions of smokers

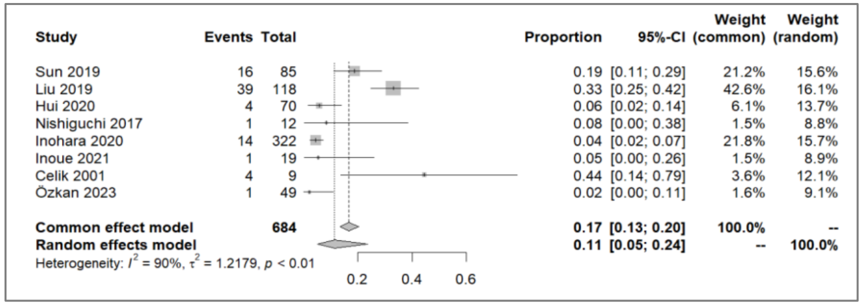

Figure S11. Pooled proportions of history of prior myocardial infarction

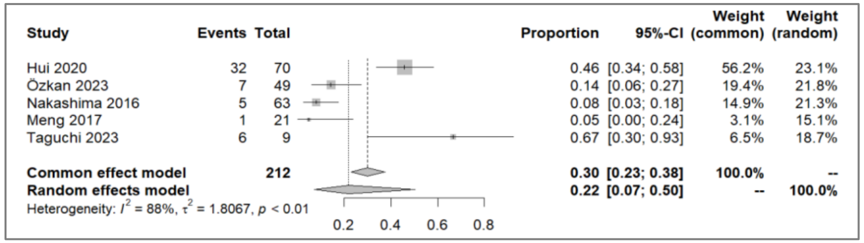

Figure S12. Pooled proportions of history of fibromuscular dysplasia

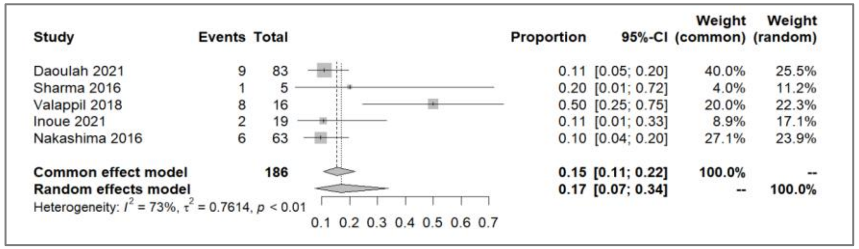

Figure S13. Pooled proportions of exposure to physical stress

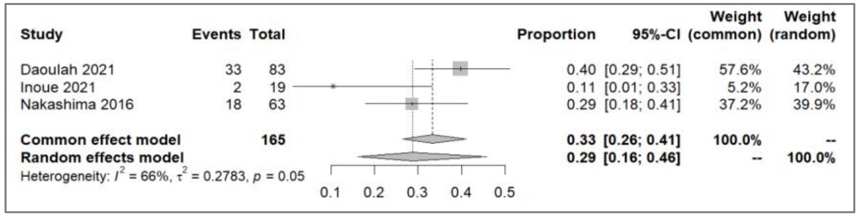

Figure S14. Pooled proportions of exposure to emotional stress

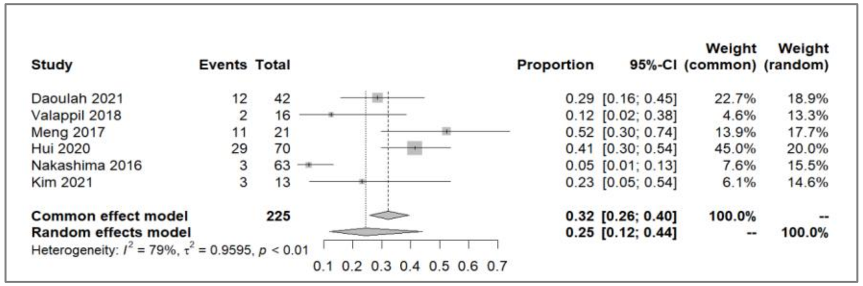

Figure S15. Pooled proportions of females in menopause

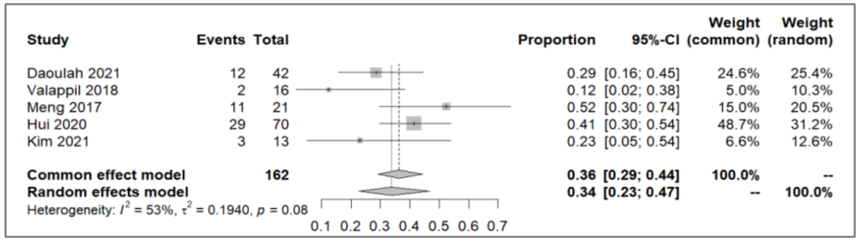

Figure S16. Pooled proportions of females in menopause without Nakashima et al study (early menopause)

Hospital presentation

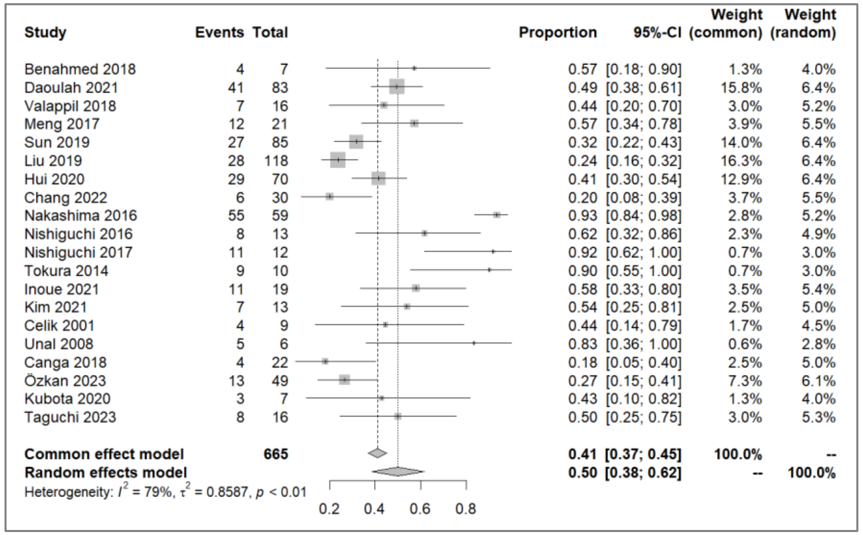

Figure S17. Pooled proportions of ST-segment elevation myocardial infarction

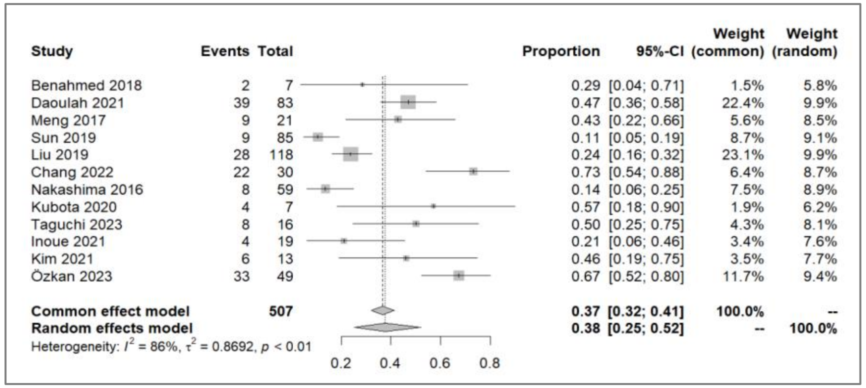

Figure S18. Pooled proportions of non-ST-segment elevation myocardial infarction

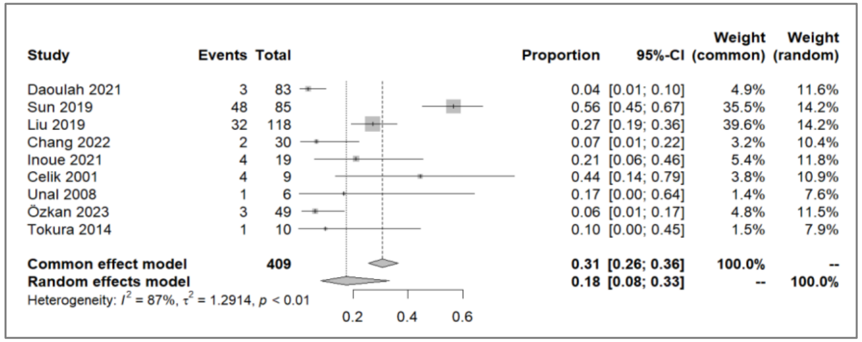

Figure S19. Pooled proportions of unstable angina

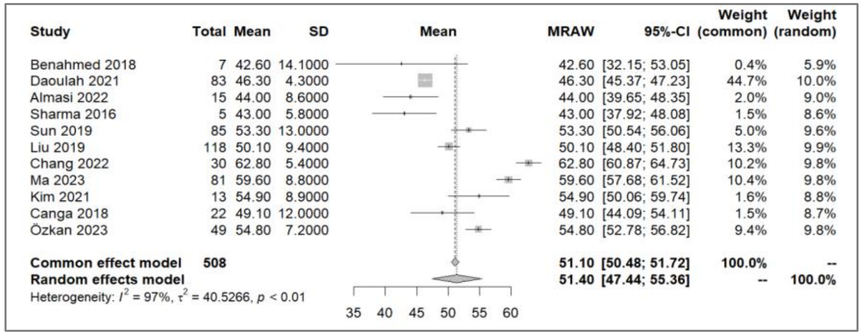

Figure S20. Pooled means of left ventricular ejection fraction

In-hospital management

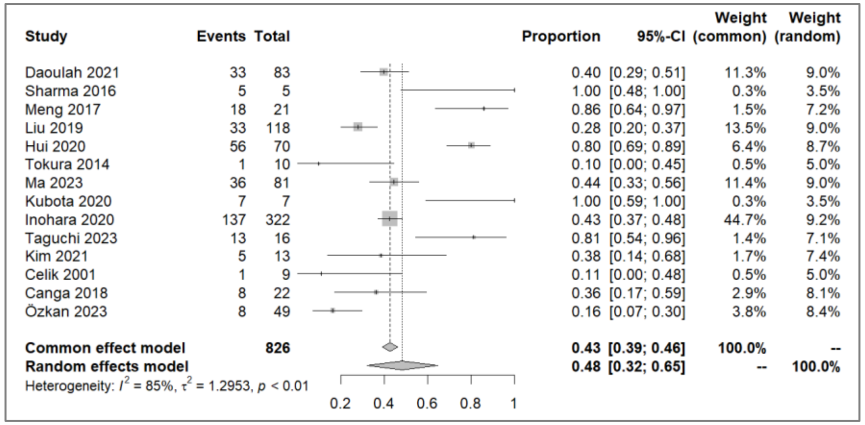

Figure S21. Pooled proportions of conservative revascularization management

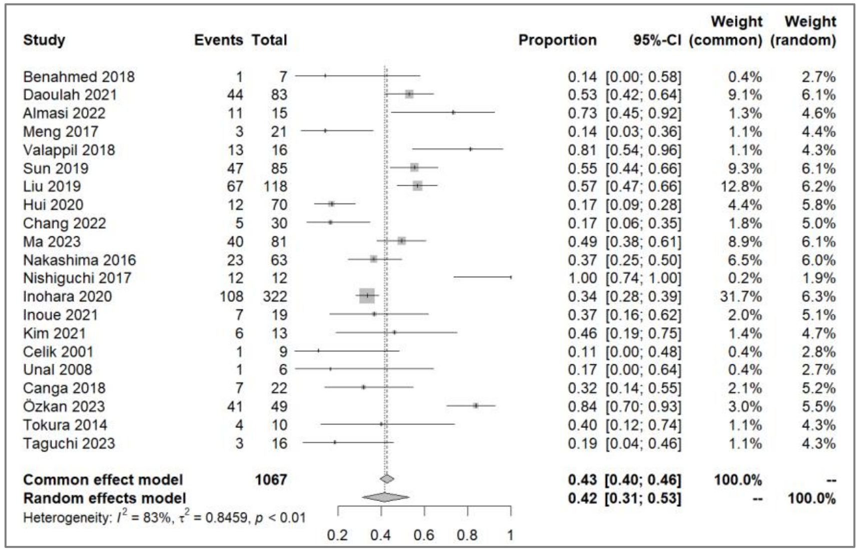

Figure S22. Pooled proportions of undergoing percutaneous coronary artery intervention

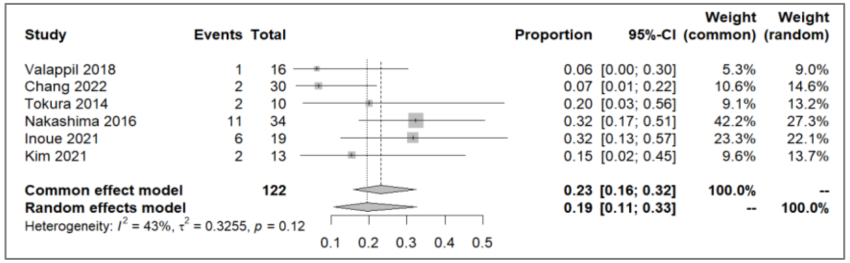

Figure S23. Pooled proportions of undergoing balloon angioplasty

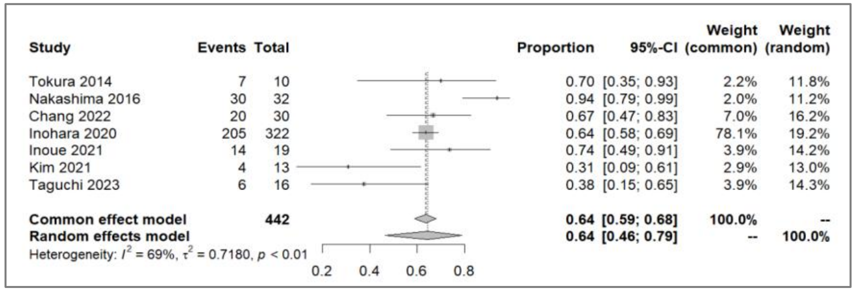

Figure S24. Pooled proportions of undergoing intravascular ultrasound

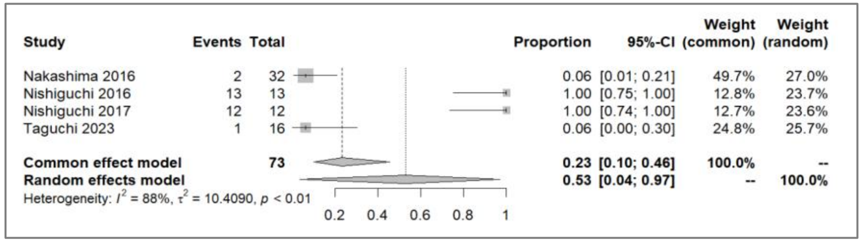

Figure S25. Pooled proportions of undergoing optical coherence tomography

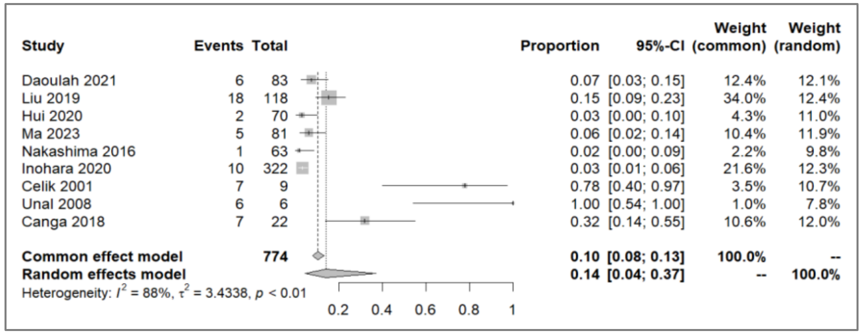

Figure S26. Pooled proportions of undergoing coronary artery bypass grafting

SCAD lesion characteristics

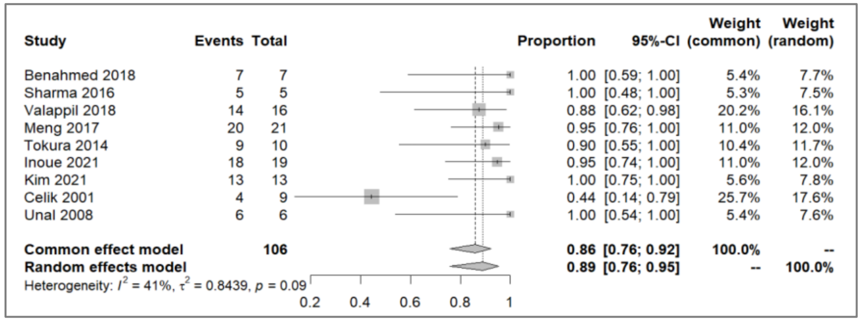

Figure S27. Pooled proportions of one-lesion SCAD

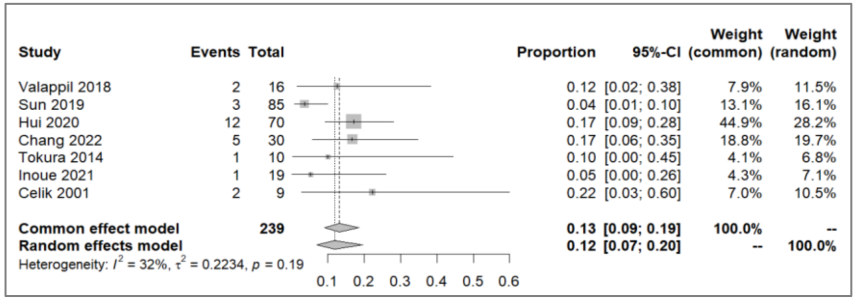

Figure S28. Pooled proportions of two-lesion SCAD

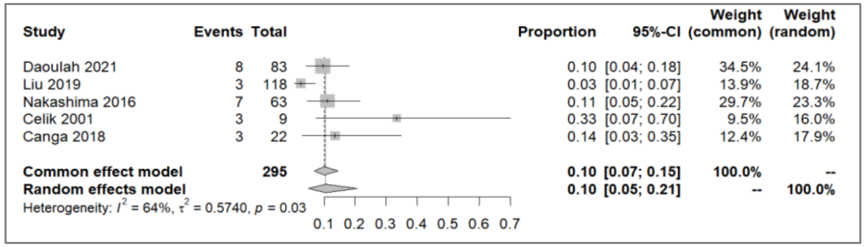

Figure S29. Pooled proportions of multivessel SCAD

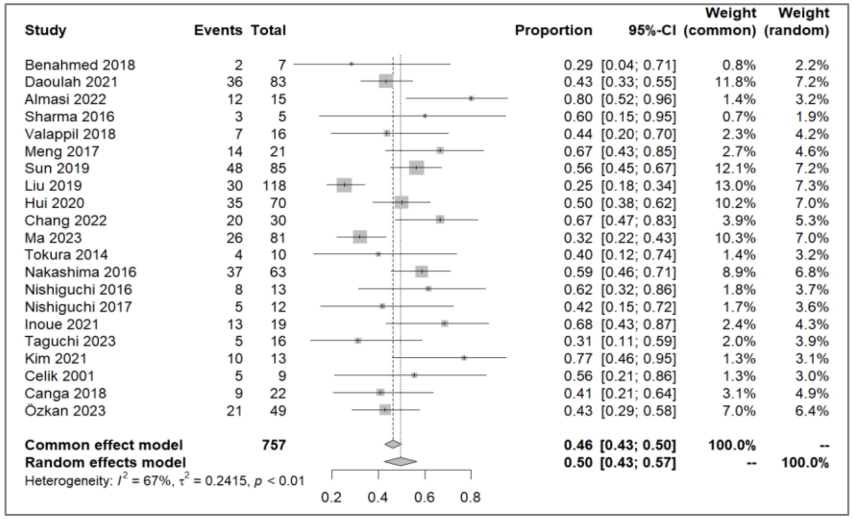

Figure S30. Pooled proportions of left anterior descending artery involvement

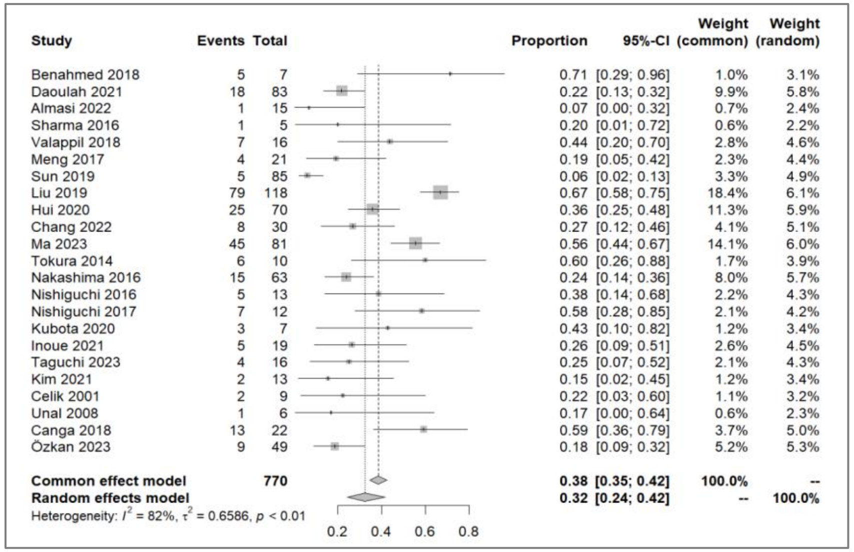

Figure S31. Pooled proportions of right coronary artery involvement

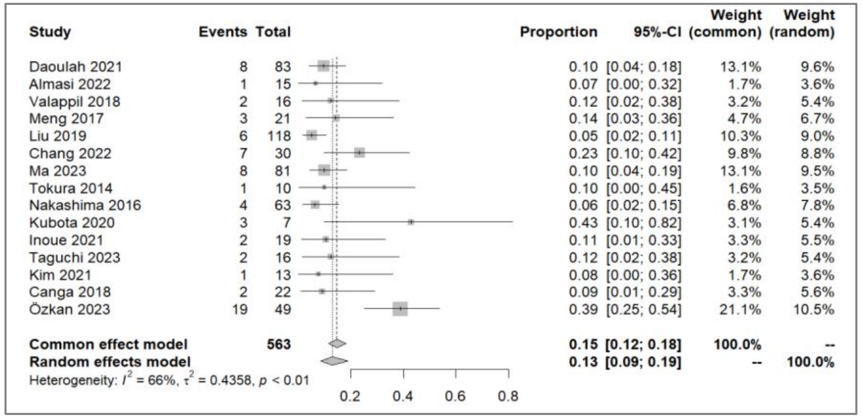

Figure S33. Pooled proportions of left circumflex artery involvement

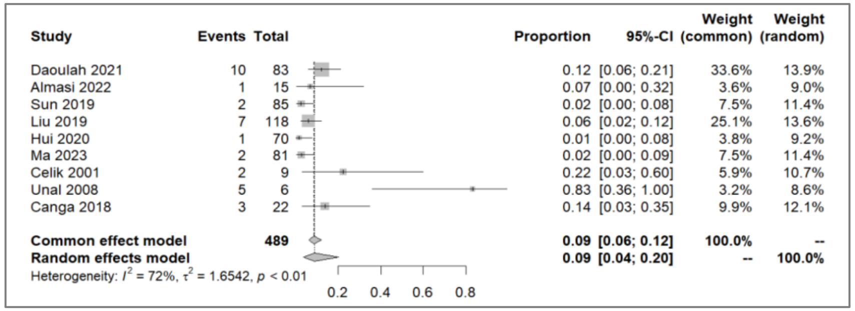

Figure S33. Pooled proportions of left main coronary artery involvement

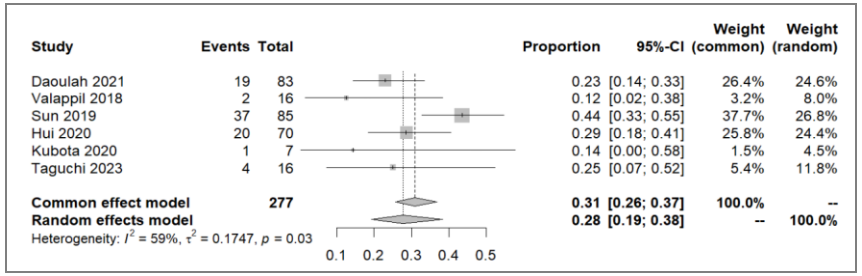

Figure S34. Pooled proportions of coronary arteries branches involvement

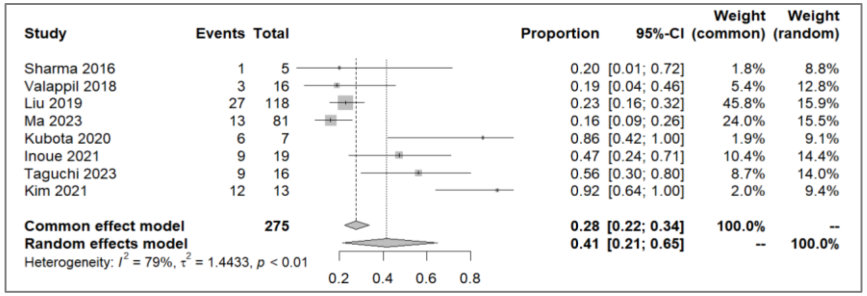

Figure S35. Pooled proportions of distal segments involvement

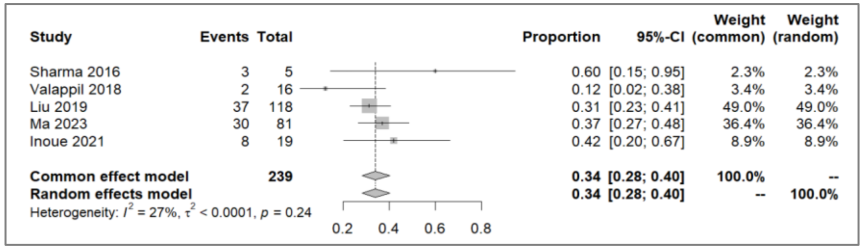

Figure S36. Pooled proportions of middle segments involvement

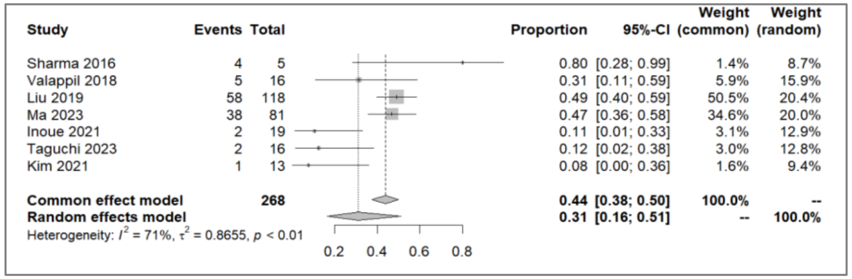

Figure S37. Pooled proportions of proximal segments involvement

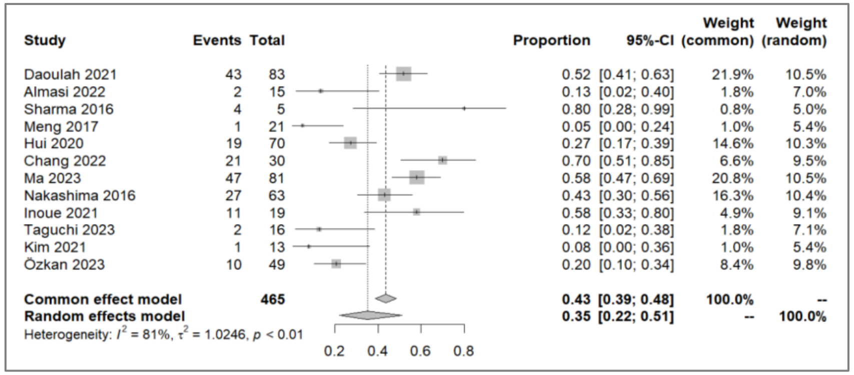

Figure S38. Pooled proportions of Type 1 SCAD

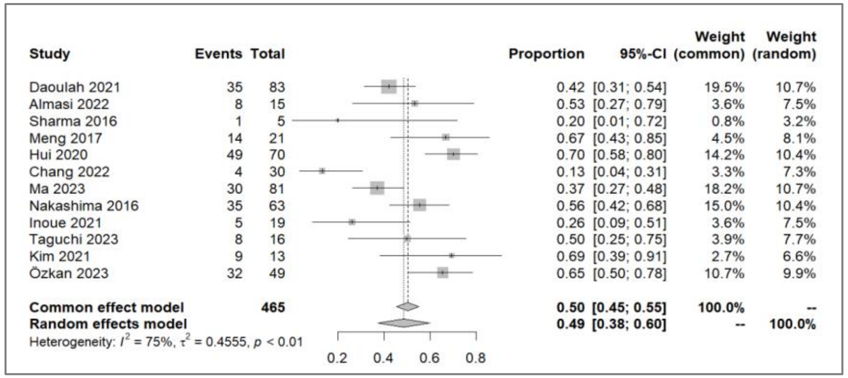

Figure S39. Pooled proportions of Type 2 SCAD

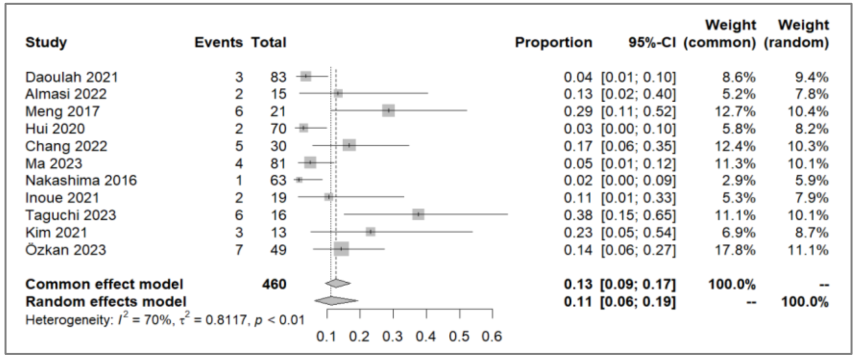

Figure S40. Pooled proportions of Type 3 SCAD

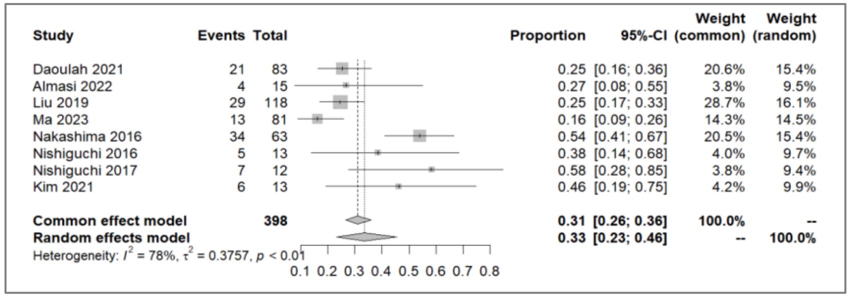

Figure S41. Pooled proportions of initial TIMI flow grade 0 or I

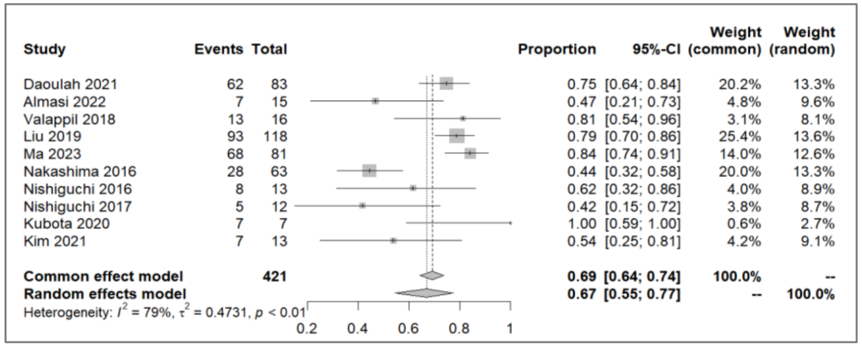

Figure S42. Pooled proportions of initial TIMI flow grade II or III

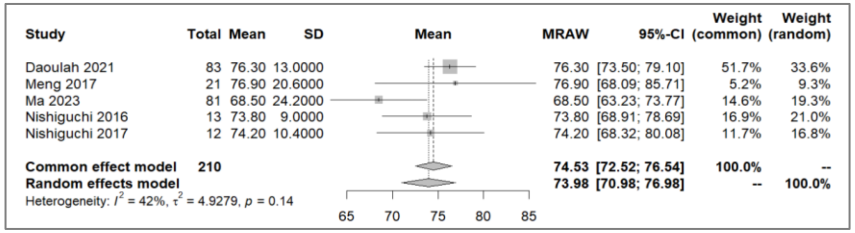

Figure S43. Pooled means of percentage of stenosis severity

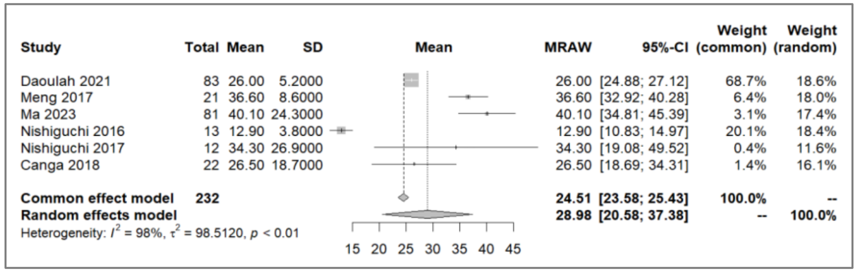

Figure S44. Pooled means SCAD lesion length (mm)

Medications upon discharge

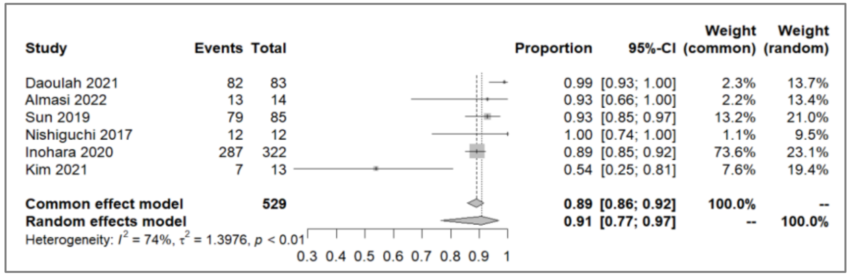

Figure S45. Pooled proportions of aspirin use

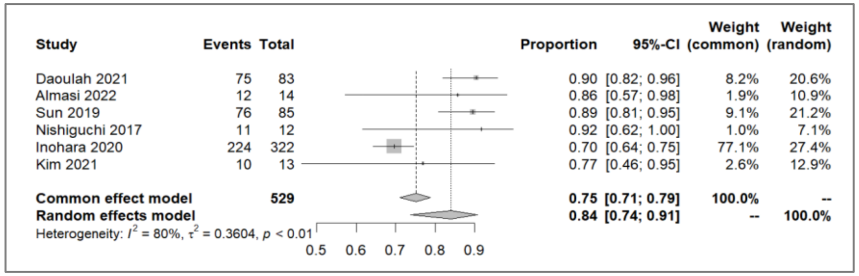

Figure S46. Pooled proportions of P_2Y_{12} inhibitors use

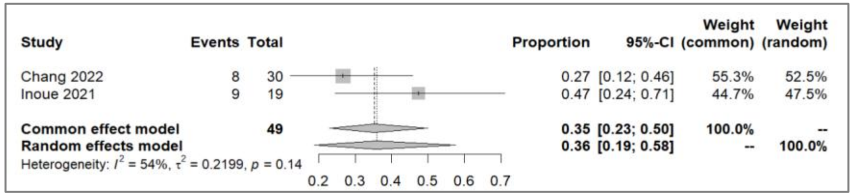

Figure S47. Pooled proportions of single antiplatelet therapy use

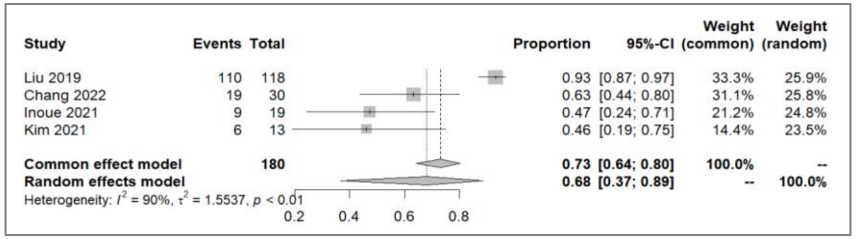

Figure S48. Pooled proportions of dual antiplatelet use

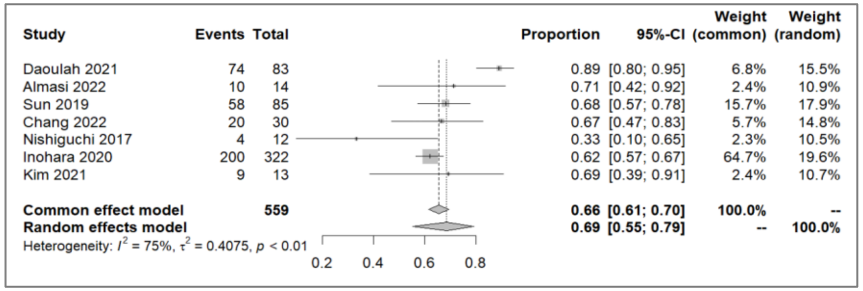

Figure S49. Pooled proportions of beta-blockers use

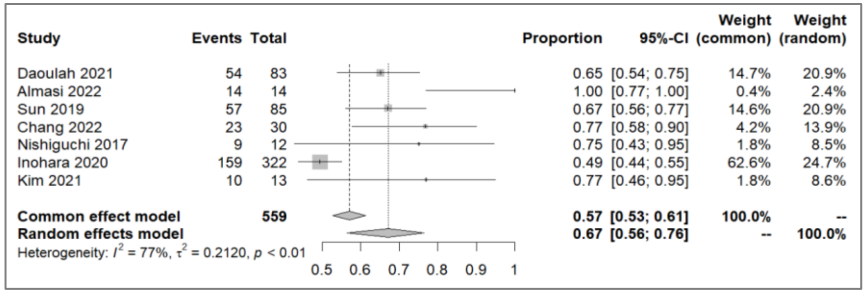

Figure S50. Pooled proportions of renin-angiotensin-aldosterone system inhibitors use

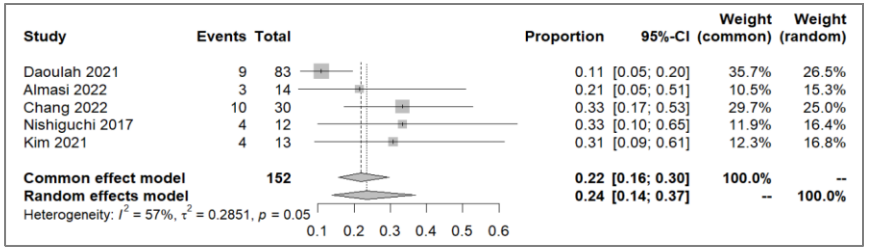

Figure S51. Pooled proportions of calcium channel blockers use

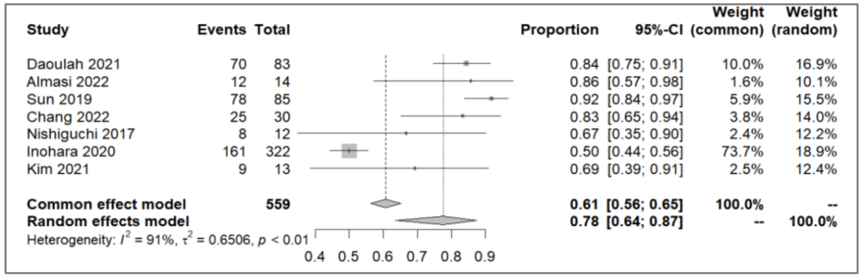

Figure S52. Pooled proportions of statin therapy use

In-hospital outcomes

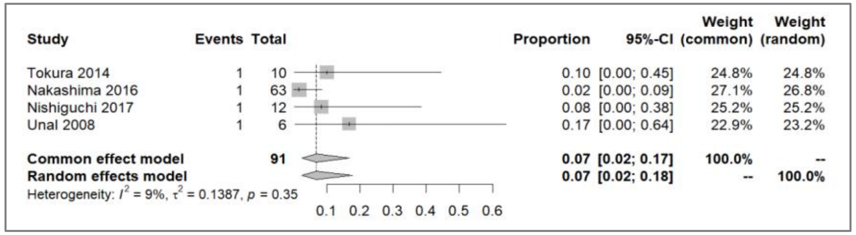

Figure S53. Pooled proportions of PCI-related complications (e.g., stent thrombosis, iatrogenic dissection)

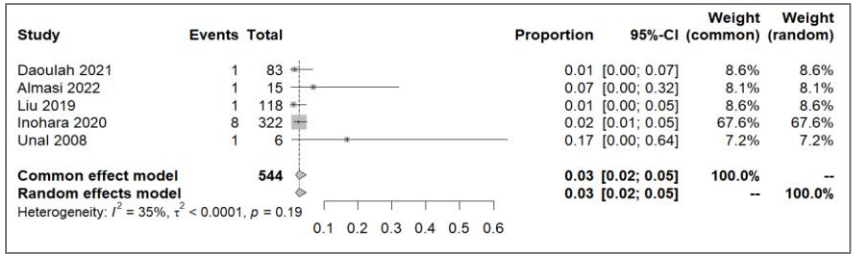

Figure S54. Pooled proportions of in-hospital death

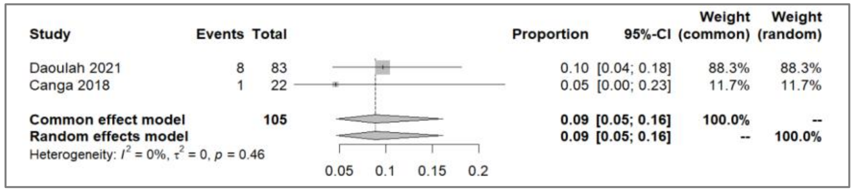

Figure S55. Pooled proportions of any in-hospital cardiovascular event

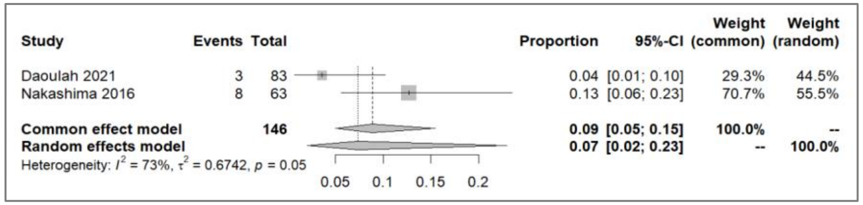

Figure S56. Pooled proportions of extension of dissection

Outcomes at follow-up

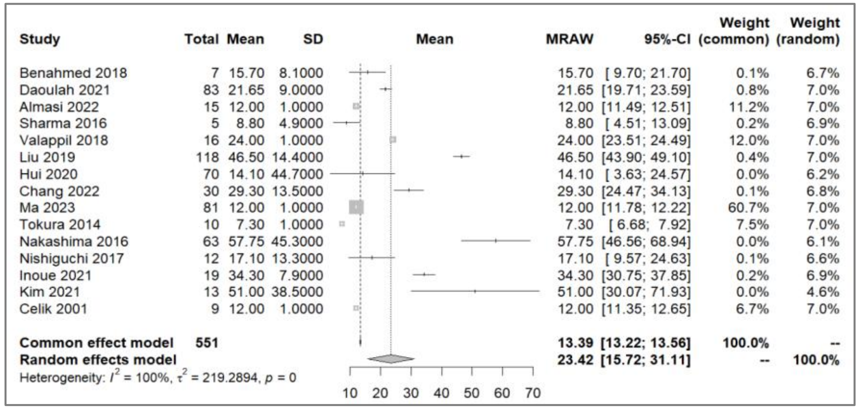

Figure S57. Pooled means of follow-up durations

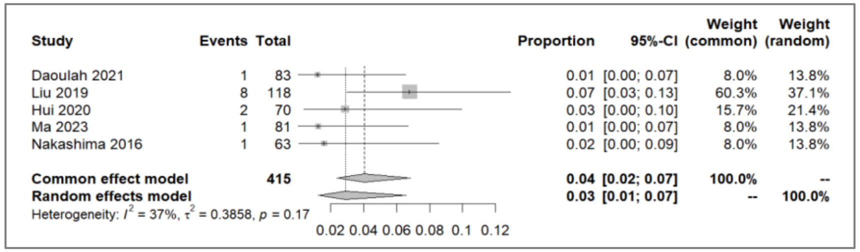

Figure S58. Pooled proportions of all-cause death

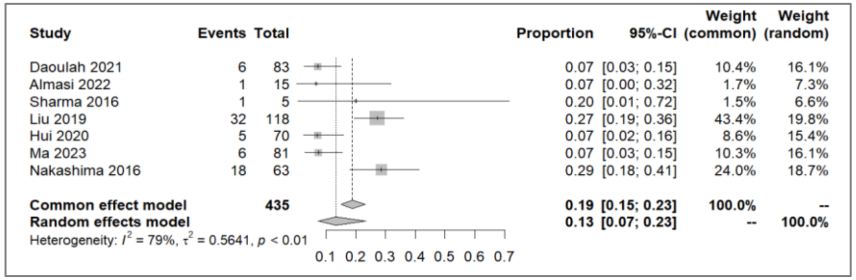

Figure S59. Pooled proportions of myocardial infarction events

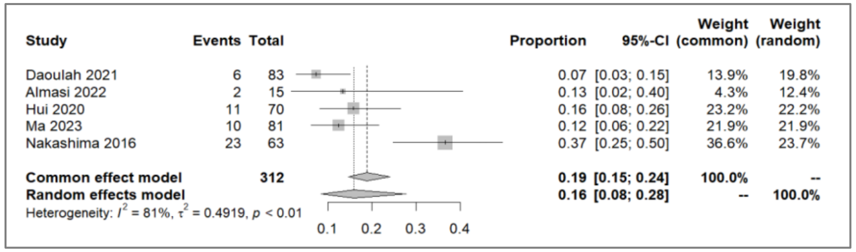

Figure S60. Pooled proportions of any cardiovascular event

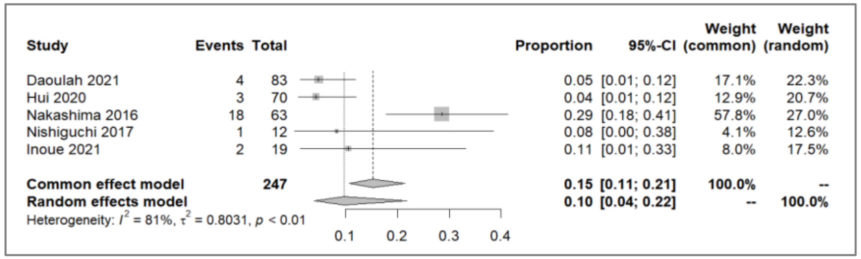

Figure S61. Pooled proportions of any SCAD event (de novo, recurrent, progressive)

References of studies in Tables S5 – S20
(including cohort studies)

WHO Eastern Mediterranean region

- Benahmed I, El Kasimi A, Laachach H, Ismaili N, Elouafi N. Spontaneous Coronary Artery Dissection (SCAD): A Series of 7 Cases, Experience of the University Hospital Center Mohammed VI, Oujda, Morocco. *Adv Med*. 2018;2018:1964394. Published 2018 Oct 2. doi:10.1155/2018/1964394
- Daoulah A, Al-Faifi SM, Alhamid S, et al. Spontaneous Coronary Artery Dissection in the Gulf: G-SCAD Registry. *Angiology*. 2021;72(1):32-43. doi:10.1177/0003319720946974
- Almasi A, Mansouri P, Jameie M, et al. Clinical Features and Prognoses of Middle-Aged Women With ST-Elevation Myocardial Infarction With a Focus on Spontaneous Coronary Artery Dissection [published correction appears in Crit Pathw Cardiol. 2022 Jun 1;21(2):103]. *Crit Pathw Cardiol*. 2022;21(1):18-23. doi:10.1097/HPC.0000000000000275

WHO South-East Asia region

- Sharma S, Raut N, Potdar A. Spontaneous coronary artery dissection: Case series and review of literature. *Indian Heart J*. 2016;68(4):480-485. doi:10.1016/j.ihj.2015.11.039
- Valappil SP, Iype M, Viswanathan S, Koshy AG, Gupta PN, Velayudhan RV. Coronary angioplasty in spontaneous coronary artery dissection-Strategy and outcomes. *Indian Heart J*. 2018;70(6):843-847. doi:10.1016/j.ihj.2018.01.004

WHO Western Pacific Region

- Rashid HN, Wong DT, Wijesekera H, et al. Incidence and characterisation of spontaneous coronary artery dissection as a cause of acute coronary syndrome--A single-centre Australian experience. *Int J Cardiol*. 2016;202:336-338. doi:10.1016/j.ijcard.2015.09.072
- McGrath-Cadell L, McKenzie P, Emmanuel S, Muller DW, Graham RM, Holloway CJ. Outcomes of patients with spontaneous coronary artery dissection. *Open Heart*. 2016;3(2):e000491. Published 2016 Aug 24. doi:10.1136/openhrt-2016-000491
- Adams H, Paratz E, Somaratne J, et al. Different patients, different outcomes: A case-control study of spontaneous coronary artery dissection versus acute coronary syndrome. *J Interv Cardiol*. 2018;31(1):41-47. doi:10.1111/joic.12447
- Yuvaraj J, Lin A, Nerlekar N, et al. Is spontaneous coronary artery dissection (SCAD) related to vascular inflammation and epicardial fat? -insights from computed tomography coronary angiography. *Cardiovasc Diagn Ther*. 2020;10(2):239-241. doi:10.21037/cdt.2020.01.09
- Fahey JK, Chew A, Ihdayhid AR, et al. Women With Spontaneous Coronary Artery Dissection Are at Increased Risk of Iatrogenic Coronary Artery Dissection. *Heart Lung Circ*. 2021;30(1):e23-e28. doi:10.1016/j.hlc.2020.06.028
- Murphy BM, Rogerson MC, Hesselson S, Iismaa SE, Graham RM, Jackson AC. Psychosocial impacts of spontaneous coronary artery dissection: A qualitative study. *PLoS One*. 2022;17(9):e0273978. Published 2022 Sep 6. doi:10.1371/journal.pone.0273978
- Murphy BM, Rogerson MC, Hesselson S, et al. Prevalence of Anxiety, Depression, and Distress in SCAD and Non-SCAD AMI Patients: A Comparative Study. *J Cardiopulm Rehabil Prev*. 2023;43(5):338-345. doi:10.1097/HCR.0000000000000782

- Murphy BM, Rogerson MC, Le Grande MR, et al. Psychosocial and lifestyle impacts of spontaneous coronary artery dissection: A quantitative study. *PLoS One*. 2024;19(1):e0296224. Published 2024 Jan 5. doi:10.1371/journal.pone.0296224
- Dang Q, Murphy B, Graham RM, et al. Patients' perspective of quality-of-care and its correlation to quality-of-life following spontaneous coronary artery dissection. *Eur J Cardiovasc Nurs*. Published online September 14, 2023. doi:10.1093/eurjcn/zvad096
- Tarr I, Hesselson S, Iismaa SE, et al. Exploring the Genetic Architecture of Spontaneous Coronary Artery Dissection Using Whole-Genome Sequencing. *Circ Genom Precis Med*. 2022;15(4):e003527. doi:10.1161/CIRCGEN.121.003527
- Tarr I, Hesselson S, Troup M, et al. Polygenic Risk in Families With Spontaneous Coronary Artery Dissection. *JAMA Cardiol*. 2024;9(3):254-261. doi:10.1001/jamacardio.2023.5194
- McAlister CP, Yi M, Adamson PD, et al. Trends in the Detection, Management and 30-Day Outcomes of Spontaneous Coronary Artery Dissection: A Six-Year, New Zealand Centre Experience. *Heart Lung Circ*. 2021;30(1):78-85. doi:10.1016/j.hlc.2020.06.020
- Wong B, To A, El-Jack S. Spontaneous coronary artery dissection: insights from computed tomography coronary angiography follow-up. *N Z Med J*. 2022;135(1555):41-47. Published 2022 May 20.
- Meng PN, Xu C, You W, et al. Spontaneous Coronary Artery Dissection as a Cause of Acute Myocardial Infarction in Young Female Population: A Single-center Study. *Chin Med J (Engl)*. 2017;130(13):1534-1539. doi:10.4103/0366-6999.208245
- Sun Y, Chen Y, Li Y, et al. Association of TSR1 Variants and Spontaneous Coronary Artery Dissection. *J Am Coll Cardiol*. 2019;74(2):167-176. doi:10.1016/j.jacc.2019.04.062
- Liu X, Xu C, Liu C, Su X. Clinical characteristics and long-term prognosis of spontaneous coronary artery dissection: A single-center Chinese experience. *Pak J Med Sci*. 2019;35(1):106-112. doi:10.12669/pjms.35.1.321
- Hui P, Bai Y, Su X, et al. The value of plasma fibrillin-1 level in patients with spontaneous coronary artery dissection. *Int J Cardiol*. 2020;302:150-156. doi:10.1016/j.ijcard.2019.12.015
- Chang S, Dai Y, Song X, et al. Characteristics, Management, and Prognosis of Spontaneous Coronary Intramural Hematoma. *Angiology*. 2022;73(4):374-379. doi:10.1177/00033197211036220
- Ma Y, Zhong X, Yin J, et al. Treatment strategy for spontaneous coronary artery dissection based on anatomical characteristics. *Eur J Med Res*. 2023;28(1):29. Published 2023 Jan 16. doi:10.1186/s40001-023-00986-y
- Tokura M, Taguchi I, Kageyama M, et al. Clinical features of spontaneous coronary artery dissection. *J Cardiol*. 2014;63(2):119-122. doi:10.1016/j.jjcc.2013.07.001
- Nakashima T, Noguchi T, Haruta S, et al. Prognostic impact of spontaneous coronary artery dissection in young female patients with acute myocardial infarction: A report from the Angina Pectoris-Myocardial Infarction Multicenter Investigators in Japan. *Int J Cardiol*. 2016;207:341-348. doi:10.1016/j.ijcard.2016.01.188
- Nishiguchi T, Tanaka A, Ozaki Y, et al. Prevalence of spontaneous coronary artery dissection in patients with acute coronary syndrome. *Eur Heart J Acute Cardiovasc Care*. 2016;5(3):263-270. doi:10.1177/2048872613504310

- Nishiguchi T, Tanaka A, Taruya A, et al. Prognosis of spontaneous coronary artery dissection treated by percutaneous coronary intervention with optical coherence tomography. *J Cardiol*. 2017;70(6):524-529. doi:10.1016/j.jjcc.2017.03.009
- Kubota N, Ozaki K, Tanabe Y, et al. Usefulness of Repeat Angiography to Establish Spontaneous Coronary Artery Dissection in Small Vessels. *Circ Rep*. 2020;2(12):739-743. Published 2020 Oct 29. doi:10.1253/circrep.CR-20-0100
- Inohara T, Saw J, Kohsaka S, Fukuda K, Fushimi K. Treatment pattern and outcome of spontaneous coronary artery dissection in Japan. *Int J Cardiol*. 2020;316:13-18. doi:10.1016/j.ijcard.2020.04.082
- Inoue Y, Tanaka A, Asano H, et al. Clinical characteristics and treatment of Spontaneous Coronary Artery Dissection in Young Women Undergoing Percutaneous Coronary Intervention. *J Cardiovasc Med (Hagerstown)*. 2021;22(1):14-19. doi:10.2459/JCM.0000000000001067
- Taguchi E, Toyofuku T, Fukuda T, et al. Fibromuscular dysplasia of the brachial artery in patients with spontaneous coronary artery dissection: a case series and literature review. *Heart Vessels*. 2023;38(10):1228-1234. doi:10.1007/s00380-023-02280-7
- Kim Y, Han X, Ahn Y, et al. Clinical characteristics of spontaneous coronary artery dissection in young female patients with acute myocardial infarction in Korea. *Korean J Intern Med*. 2021;36(1):106-113. doi:10.3904/kjim.2019.118
- Uribe CE, Ramirez-Barrera JD, Rubio C, et al. Spontaneous coronary artery dissection: Case series from two institutions with literature review. *Anatol J Cardiol*. 2015;15(5):409-415. doi:10.5152/akd.2015.5851

Turkey

- Celik SK, Sagcan A, Altintig A, Yuksel M, Akin M, Kultursay H. Primary spontaneous coronary artery dissections in atherosclerotic patients. Report of nine cases with review of the pertinent literature. *Eur J Cardiothorac Surg*. 2001;20(3):573-576. doi:10.1016/s1010-7940(01)00864-8
- Unal M, Korkut AK, Kosem M, Ertunc V, Ozcan M, Caglar N. Surgical management of spontaneous coronary artery dissection. *Tex Heart Inst J*. 2008;35(4):402-405.
- Canga Y, Guvenc TS, Calik AN, et al. Systemic inflammatory activation in patients with acute coronary syndrome secondary to nonatherosclerotic spontaneous coronary artery dissection. *North Clin Istanb*. 2018;5(3):186-194. doi:10.14744/nci.2017.59244
- Özkan U, Gürdoğan M. TyG index as a predictor of spontaneous coronary artery dissection in young women. *Postgrad Med*. 2023;135(7):669-675. doi:10.1080/00325481.2023.2242760
